# Supplementary material for: The ribotoxic stress response drives acute inflammation, cell death, and epidermal thickening in UV-irradiated skin in vivo
Source: Mol Cell. 2024 Dec 19;84(24):4774–4789.e9. doi: 10.1016/j.molcel.2024.10.044 (PMC11671030; doi:10.1016/j.molcel.2024.10.044)
Supplement: Document S1. Figures S1–S7 [file mmc1.pdf]

Molecular Cell, Volume *84*

Supplemental information

The ribotoxic stress response drives acute inflammation, cell death, and epidermal thickening in UV-irradiated skin *in vivo*

Anna Constance Vind, Zhenzhen Wu, Muhammad Jasrie Firdaus, Goda Snieckute, Gee Ann Toh, Malin Jessen, José Francisco Martínez, Peter Haahr, Thomas Levin Andersen, Melanie Blasius, Li Fang Koh, Nina Loeth Maartensson, John E.A. Common, Mads Gyrd-Hansen, Franklin L. Zhong, and Simon Bekker-Jensen

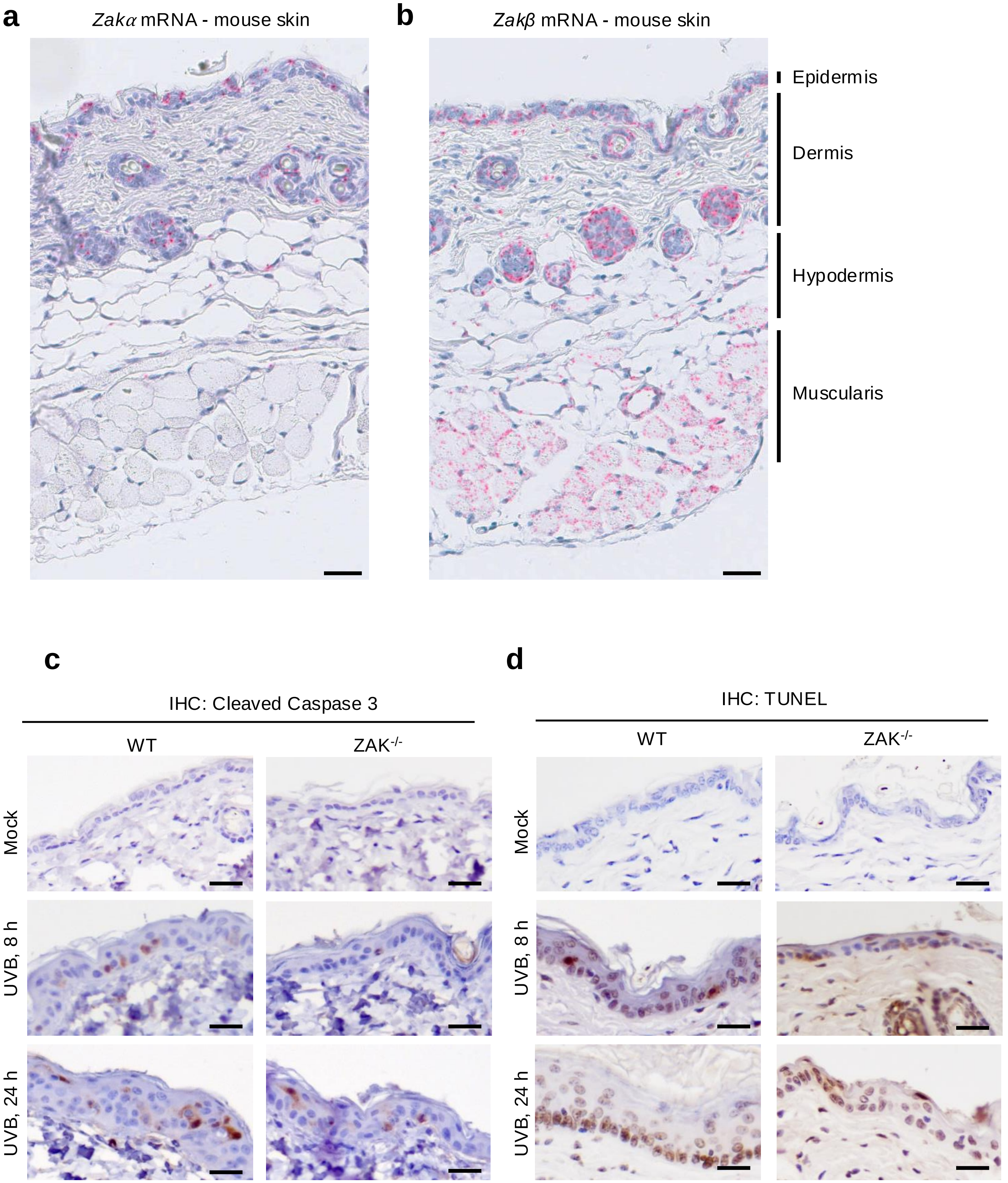
a
Zakα mRNA - mouse skin
b
Zakβ mRNA - mouse skin
Epidermis
Dermis
Hypodermis
Muscularis
c
IHC: Cleaved Caspase 3
WT
ZAK-/-
Mock
UVB, 8 h
UVB, 24 h
d
IHC: TUNEL
WT
ZAK-/-
Mock
UVB, 8 h
UVB, 24 h

Figure S1.

***Zakα* and *Zakβ* expression in mouse skin (related to Fig. 1).**

a) Paraffin-embedded mouse skin was stained for *Zakα* mRNA (pink dots) by *in situ* hybridization with a modified RNAScope protocol. **b)** As in (a), except that samples were stained for *Zakβ* mRNA. Notice the specific expression of the β isoform in muscle fibers (muscularis layer). **c)** Skins of mice from Fig. 1c were harvested 8 and 24 hours after irradiation and immunostained with an antibody recognizing cleaved caspase-3. **d)** As in (c), except that skin sections were stained with TUNEL kit for detection of dead and dying cells. All scale bars = 50 µm.

Vind et al, Figure S2

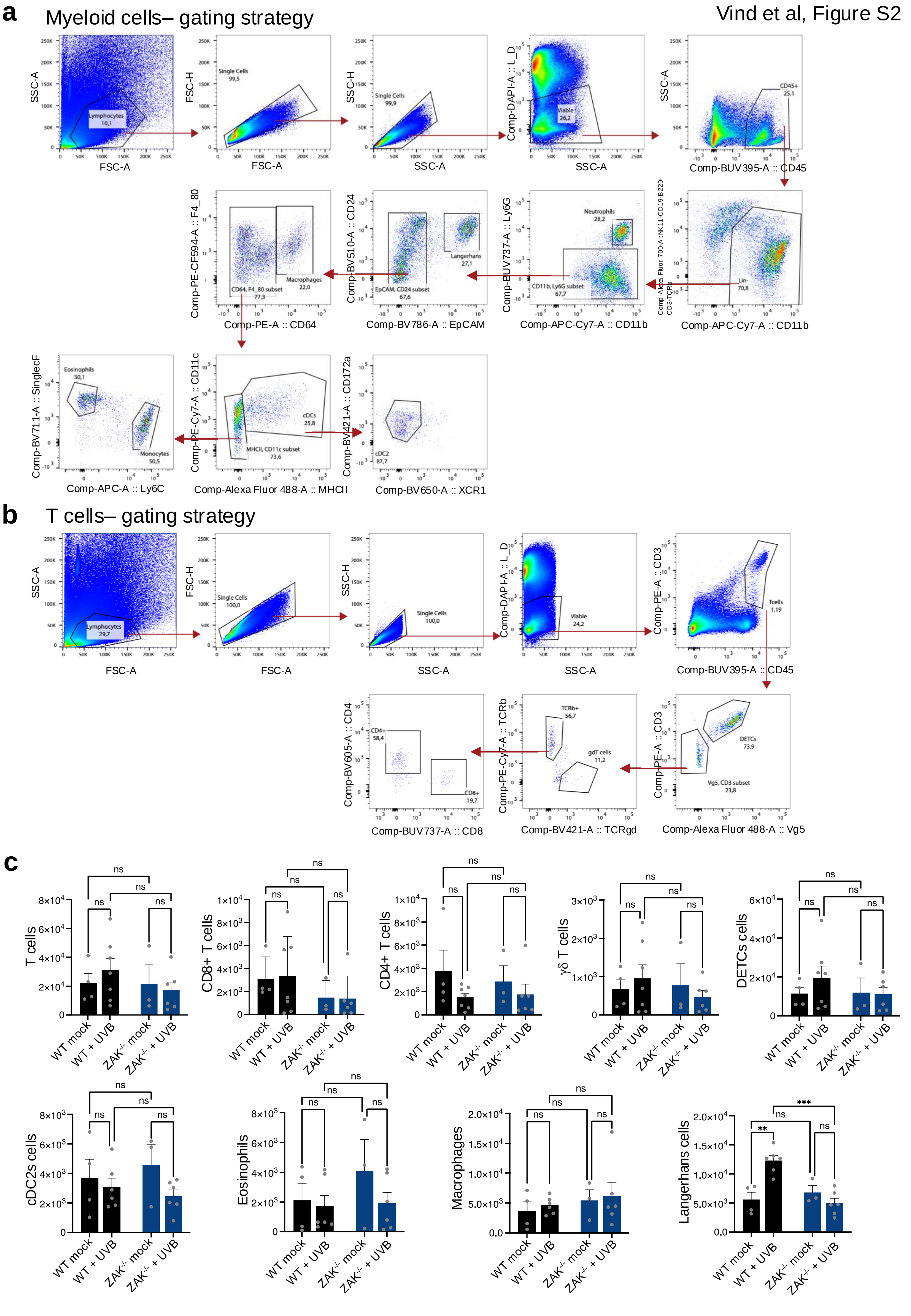

Figure S2.

Gating strategy for FACS-based analysis of dermal immune cell content (related to Fig. 2).

a-b) Schematic presentation of the gating strategy for flow cytometry analysis of **a**) myeloid and **b**) T cells in skin samples (Lineage: NK1.1, CD19, B220, CD3, TCRβ). **c**) Mouse skin samples were collected from control or UVB-exposed mice and immune infiltrates were analyzed by flow cytometry. Graphs represent the mean number of total T cells ($CD45^+$ $CD3^+$), $CD4^+$ T cells ($TCR\beta^+$ $CD4^+$), $CD8^+$ T cells ($TCR\beta^+$ $CD8^+$), γδ T cells ($CD3^+$, $TCR\gamma\delta^+$), dendritic epidermal T cells (DETCs; $CD3^+$, $V\gamma5^+$), cDC2s ($CD11c^+$ $MHCII^+$, $CD172\alpha^+$), eosinophils ($CD11b^+$ $SiglecF^+$), macrophages ($CD11b^+$, $F4/80^+$, $CD64^+$), Langerhans cells ($CD24^+$, $EpCAM^+$,). The data were obtained from two independent experiments with 3-7 mice per group. Data are presented as the mean, with error bars denoting the standard error of the mean (SEM). ns., non-significant; *, $p \leq 0.05$; **, $p \leq 0.01$; ***, $p < 0.001$; ****, $p \leq 0.0001$ in two-way ANOVA with the Sidak method.

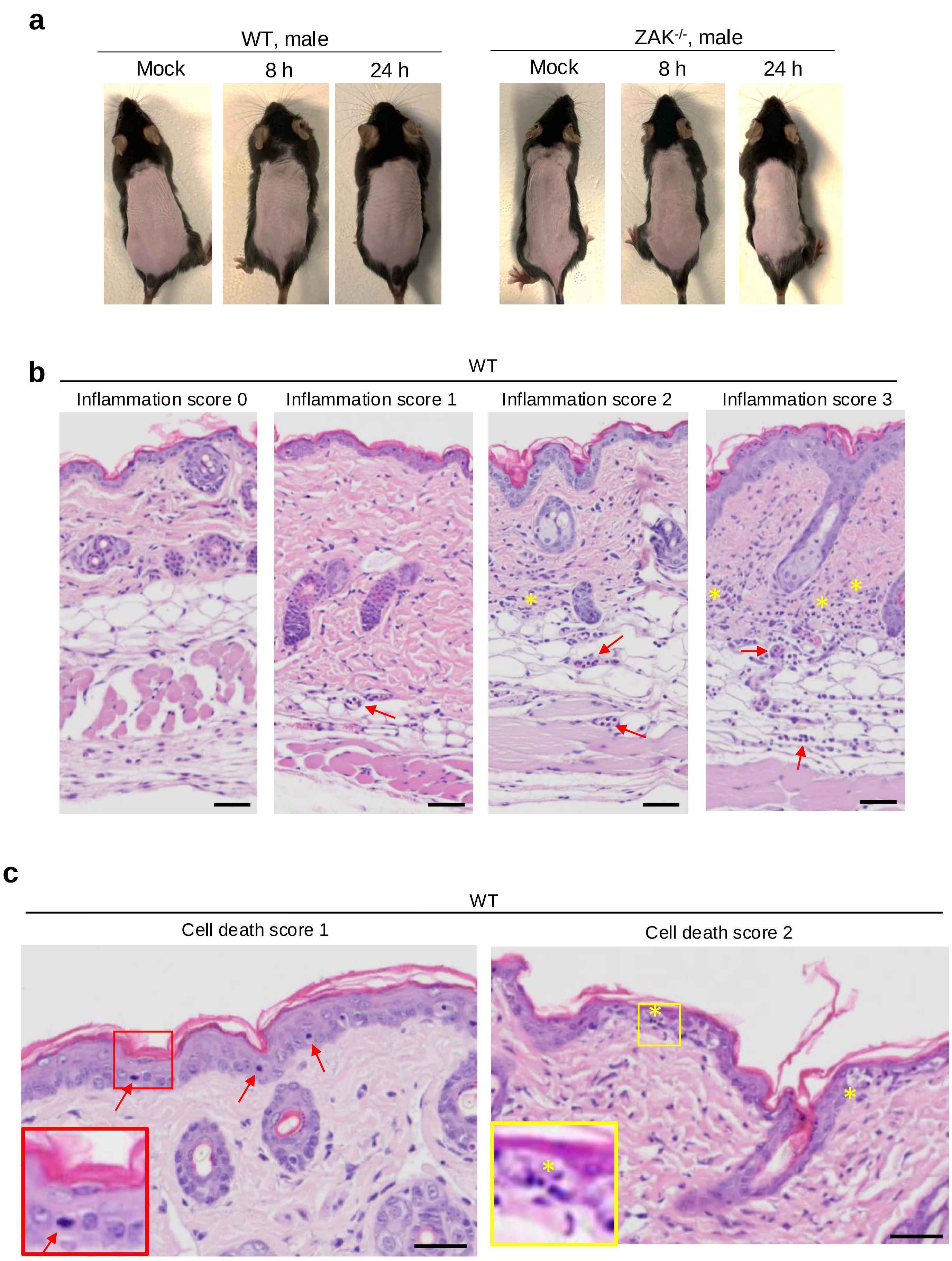
a
WT, male
Mock
8 h
24 h
ZAK-/-, male
Mock
8 h
24 h
b
WT
Inflammation score 0
Inflammation score 1
Inflammation score 2
Inflammation score 3
c
WT
Cell death score 1
Cell death score 2

Figure S3.

Scoring system for histological hallmarks of skin inflammation and cell death (related to Fig. 2).

a) Representative images of male mice 24 h and 48 h after UVB-irradiation (500 J/m^2). **b)** Hematoxylin and eosin (H/E)-stained mouse skin sections representing inflammatory scores 0-3. Red arrows indicate intraluminal infiltration and yellow asterisks indicate dermal infiltration of immune cells. **c)** Examples of discrete (upper panel, yellow arrows) and confluent (lower panel, yellow asterisks) cell death in UVB-irradiated and hematoxylin and eosin (H/E)-stained mouse skin. All scale bars = 50 µm.

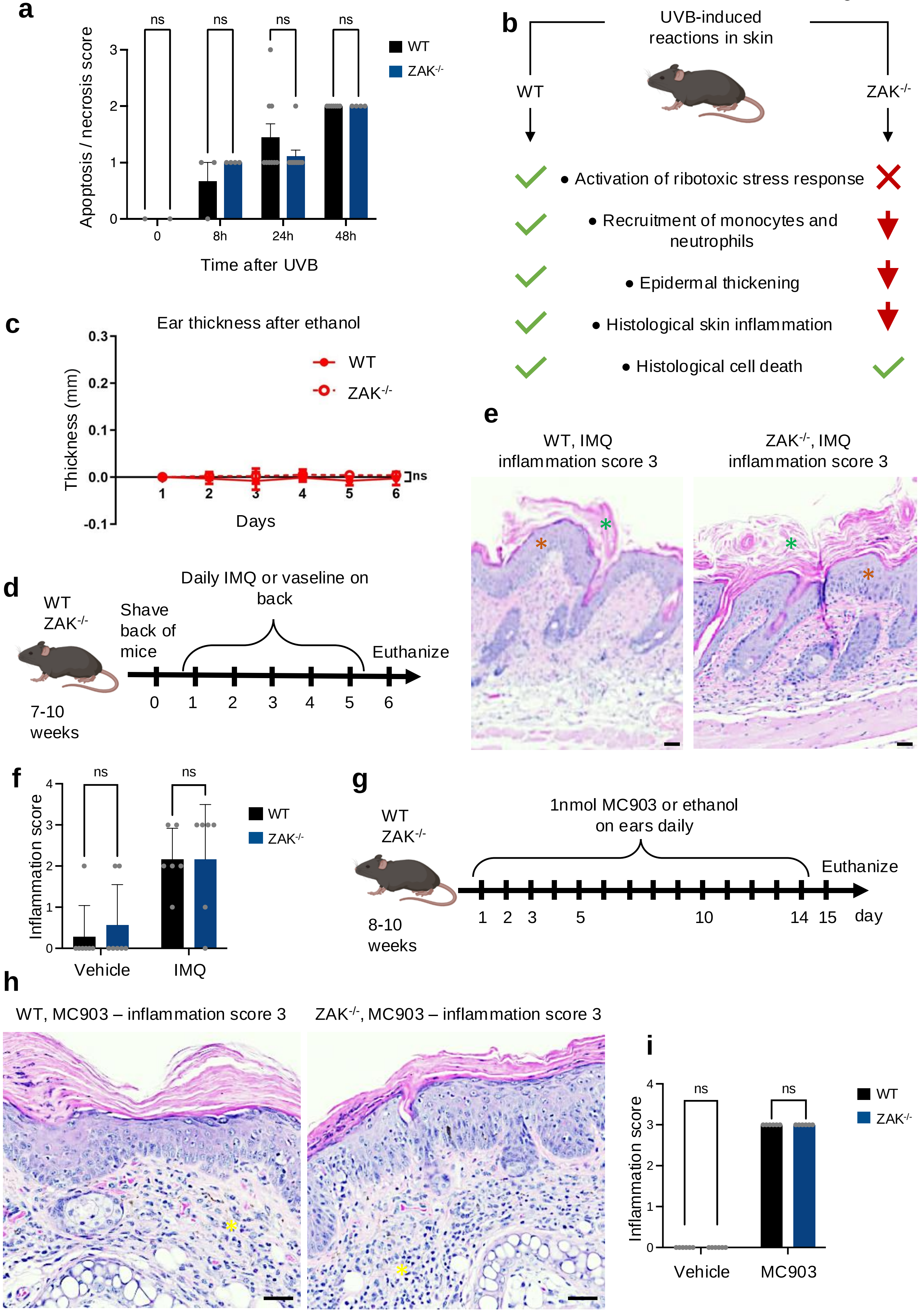
a
Apoptosis / necrosis score
ns
ns
ns
ns
WT
ZAK-/-
0
8h
24h
48h
Time after UVB
b
UVB-induced reactions in skin
WT
ZAK-/-
• Activation of ribotoxic stress response
• Recruitment of monocytes and neutrophils
• Epidermal thickening
• Histological skin inflammation
• Histological cell death
c
Ear thickness after ethanol
Thickness (mm)
WT
ZAK-/-
ns
Days
d
WT
ZAK-/-
7-10 weeks
Shave back of mice
Daily IMQ or vaseline on back
Euthanize
e
WT, IMQ inflammation score 3
ZAK-/-, IMQ inflammation score 3
f
Inflammation score
ns
ns
WT
ZAK-/-
Vehicle
IMQ
g
WT
ZAK-/-
8-10 weeks
1nmol MC903 or ethanol on ears daily
Euthanize
day
h
WT, MC903 – inflammation score 3
ZAK-/-, MC903 – inflammation score 3
i
Inflammation score
ns
ns
WT
ZAK-/-
Vehicle
MC903

Figure S4.

ZAK$^{-/-}$ mice are not protected against inflammation and epidermal thickening in standard models of inflammatory skin disease (related to Fig. 2).

a) Extent of cell death in 9-week-old female WT and ZAK$^{-/-}$ mice at 8, 24 and 48 hours after UVB-irradiation (500 J/m^2) was assessed histologically based on the following scoring system. Normal: 0. Discrete apoptotic and necrotic cells: 1. Presence of areas of confluent apoptotic and necrotic cells: 2. Representative images showed in Fig. S3c. **b)** The ribotoxic stress response is activated in mouse skin upon UVB-irradiation and mediates several of the well-known adaptive responses. UVB-irradiated ZAK$^{-/-}$ mouse skin is refractory to early inflammation and displays attenuated and delayed epidermal thickening. Keratinocyte cell death is equally pronounced in UVB-irradiated WT and ZAK$^{-/-}$ mouse skin. **c)** Growth in ear thickness of mice from Fig. 3c were measured with a caliper. Only data from vehicle-treated ears are shown. **d)** Schematic of *in vivo* mouse imiquimod (IMQ) exposure experiment. IMQ was dissolved in Vaseline (vehicle) and applied to the shaved backs of 7-10-week-old female WT and ZAK$^{-/-}$ mice for 5 consecutive days. Mice were euthanized on day 6. **e)** Representative H/E-stained images of back skin from (d). Green asterisks indicate hyperparakeratosis. Brown asterisks indicate epidermal hyperplasia. **f)** Severity of inflammation in back skins from (d) was assessed histologically based on infiltrating immune cells according to the scoring system described in the legend of Fig. 2g. **g)** Schematic of *in vivo* mouse MC903 exposure experiment. MC903 was dissolved in ethanol (vehicle) and applied to one ear of 8-10-week-old female WT and ZAK$^{-/-}$ mice for 14 consecutive days. Pure ethanol was similarly applied to the other ear and mice were euthanized on day 15. **h)** Representative H/E-stained images of ears from (c). Yellow asterisks indicate dermal infiltration of immune cells. **i)**. Ear skin inflammation of mice from (g) was scored as in (f). Data are plotted as mean and all error bars represent the standard error

of the mean (SEM) (n=3-9 biological replicates). ns., non-significant in two-way ANOVA with the Sidak method. All scale bars = 50 μm.

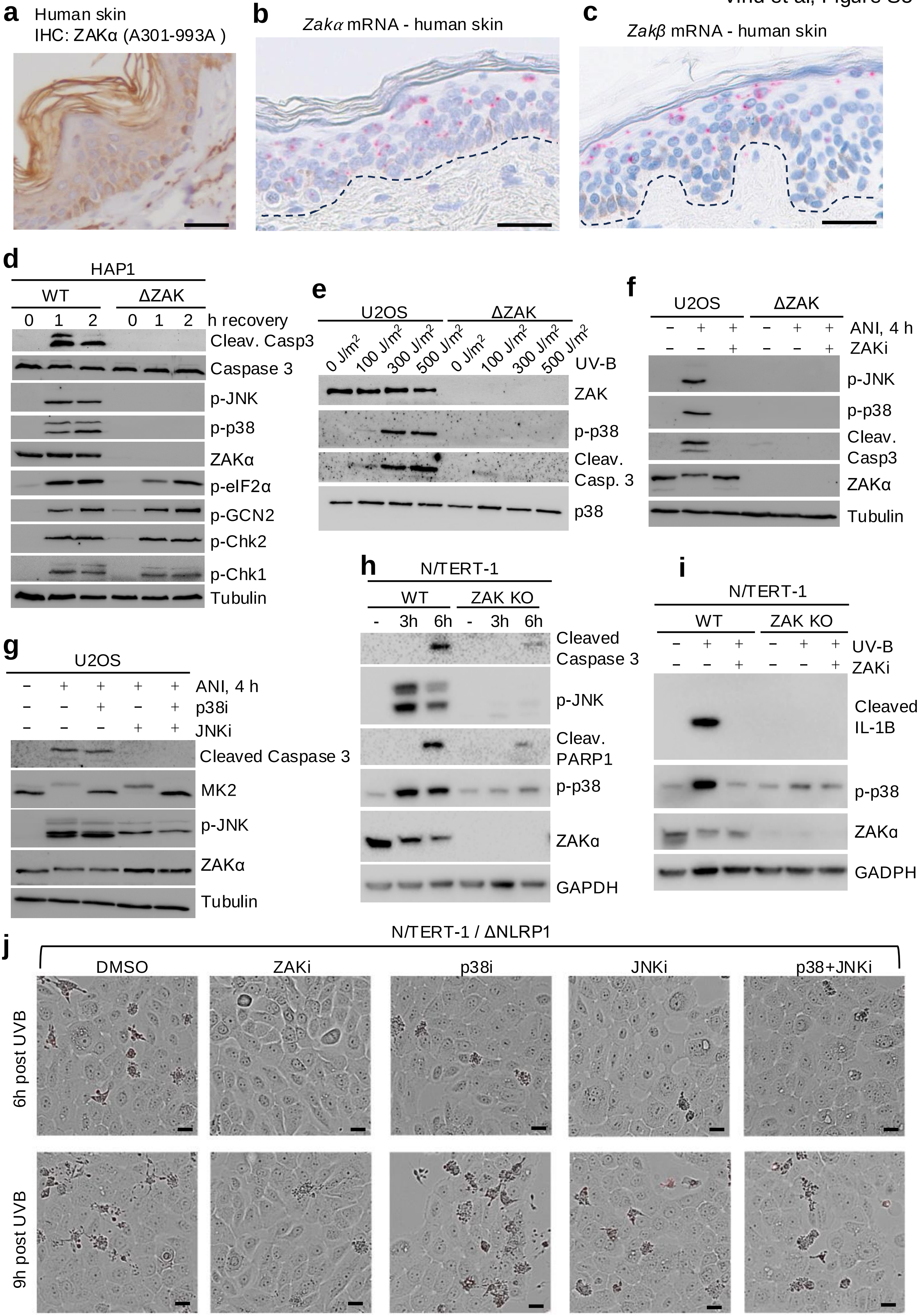
a
Human skin
IHC: ZAKα (A301-993A)
b
Zakα mRNA - human skin
c
Zakβ mRNA - human skin
d
HAP1
WT
ΔZAK
0 1 2 0 1 2 h recovery
Cleav. Casp3
Caspase 3
p-JNK
p-p38
ZAKα
p-eIF2α
p-GCN2
p-Chk2
p-Chk1
Tubulin
e
U2OS
ΔZAK
0 J/m2 100 J/m2 300 J/m2 500 J/m2 0 J/m2 100 J/m2 300 J/m2 500 J/m2
UV-B
ZAK
p-p38
Cleav. Casp. 3
p38
f
U2OS
ΔZAK
ANI, 4 h
ZAKi
p-JNK
p-p38
Cleav. Casp3
ZAKα
Tubulin
g
U2OS
ANI, 4 h
p38i
JNKi
Cleaved Caspase 3
MK2
p-JNK
ZAKα
Tubulin
h
N/TERT-1
WT
ZAK KO
- 3h 6h - 3h 6h
Cleaved Caspase 3
p-JNK
Cleav. PARP1
p-p38
ZAKα
GAPDH
i
N/TERT-1
WT
ZAK KO
UV-B
ZAKi
Cleaved IL-1B
p-p38
ZAKα
GADPH
j
N/TERT-1 / ΔNLRP1
DMSO
ZAKi
p38i
JNKi
p38+JNKi
6h post UVB
9h post UVB

Figure S5.

The ribotoxic stress response mediates UVB-induced and JNK-dependent apoptotic cell death (related to Fig. 4).

a) Paraffin-embedded human skin was analyzed for ZAKα expression (#A301-993A) by immunohistochemistry. Scale bars = 50 μm. **b)** Paraffin-embedded human skin was stained for *Zakα* mRNA (pink dots) by *in situ* hybridization with a modified RNAScope protocol. Dashed line marks the separation between epidermis and dermis. Scale bar = 10 μm. **c)** As in (b), except that *Zakβ* mRNA was detected. **d)** HAP1 WT and ΔZAK cells were UVB irradiated (500 J/m^2) and allowed to recover for the indicated times. Lysates were analyzed by immunoblotting with the indicated antibodies. **e)** U2OS WT and ΔZAK cells were irradiated with the indicated doses of UVB (8 hours recovery). Lysates were analyzed as in (d). **f)** Cells from (e) were treated with anisomycin (ani, 1 μg/ml - 4 hours) in the presence of ZAK inhibitor (i, 1 μM) as indicated. Lysates were analyzed as in (d). **g)** U2OS cells were treated with ani (1 μg/ml - 4 hours) in the presence of inhibitors (i) of p38 and JNK (2 μM). Lysates were analyzed as in (d). **h)** WT and ΔZAK N/TERT-1 cells were irradiated with UVB (100 J/m^2) and lysed at the indicated timepoints. Lysates were analyzed as in (d). **i)** As in (h), except that cells were pre-treated with ZAK inhibitor (i, 1 μM) and lysed 8 h after UVB irradiation. **j)** Representative bright-field microscopy images of NLRP1 KO N/TERT-1 cells in combination with the indicated inhibitor 6 hours and 9 hours post UVB to show apoptotic/necrotic cell death. Scale bars = 10 μm.

Vind et al, Figure S6

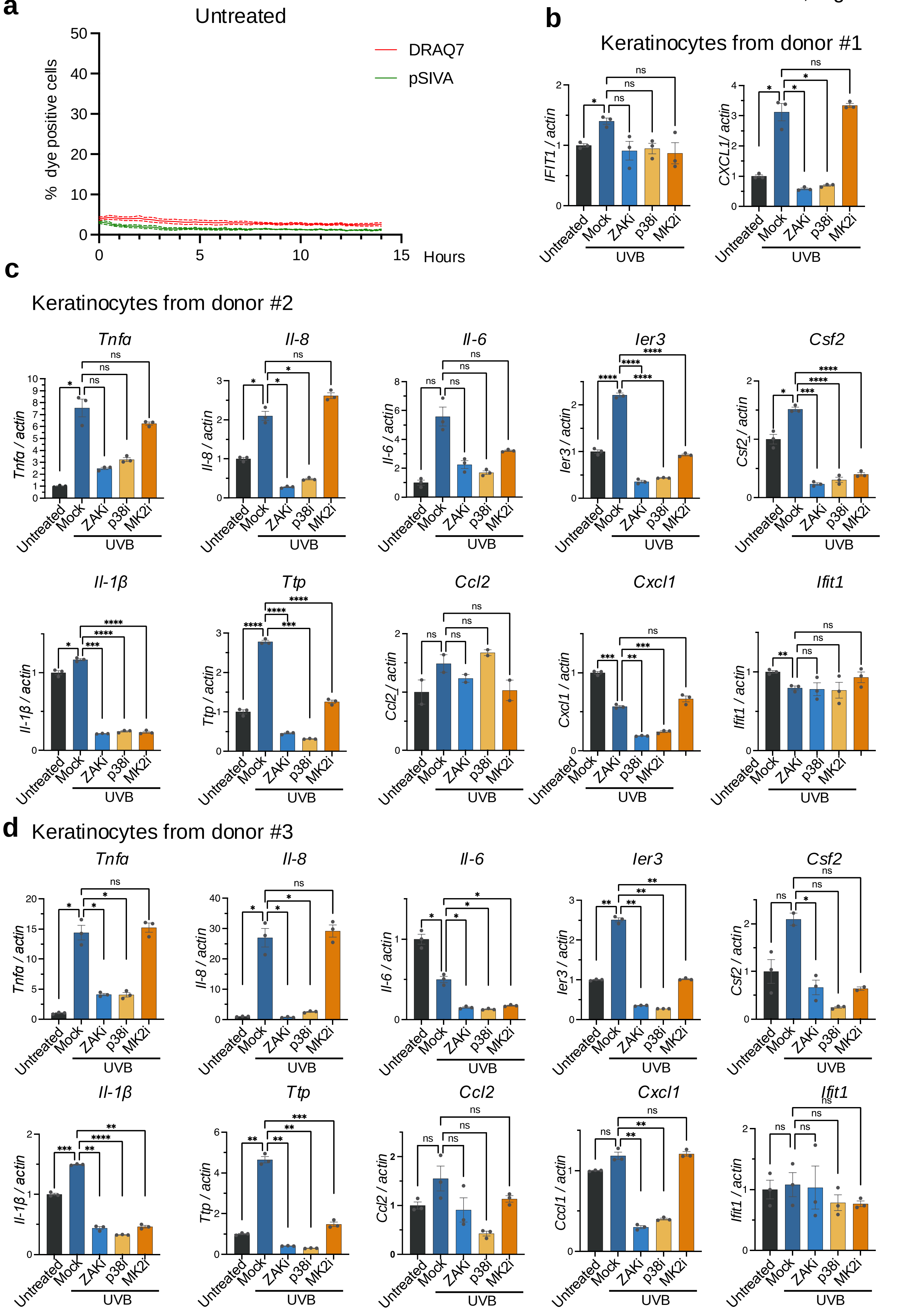

Figure S6.

Upregulation of inflammatory transcripts in UVB-irradiated primary human keratinocytes (related to Fig. 5).

a) N/TERT-1 cells were grown in the presence of the live cell staining dyes DRAQ7 (permeabilized cells) and pSIVA (apoptosis marker) and subjected to fluorescence time-lapse microscopy. Percentage of dye-including cells are plotted as mean and error bars represent the standard error of the mean (SEM). Error bars are from 3 technical replicates that are representative of 2 independent repeats. **b)** Primary human keratinocytes from donor in Fig. 5f were UVB irradiated (500 J/m^2 – 6 hours) in the presence of inhibitors (i, 2 μM) against the kinases ZAK, p38 and JNK as indicated. qPCR analysis of isolated RNA was performed using primers against the indicated transcripts. Values were normalized to actin expression and transformed into fold changes over the values from untreated cells. **c)** and **d)** as in (b), but with primary keratinocytes from two independent human donors. Fold-change data are plotted as mean and all error bars represent the standard deviation (SD) (n=3 technical replicates). ns., non-significant; *, $p \leq 0.05$; **, $p \leq 0.01$; ***, $p<0.001$; ****, $p \leq 0.0001$ in two-way ANOVA with the Sidak method.

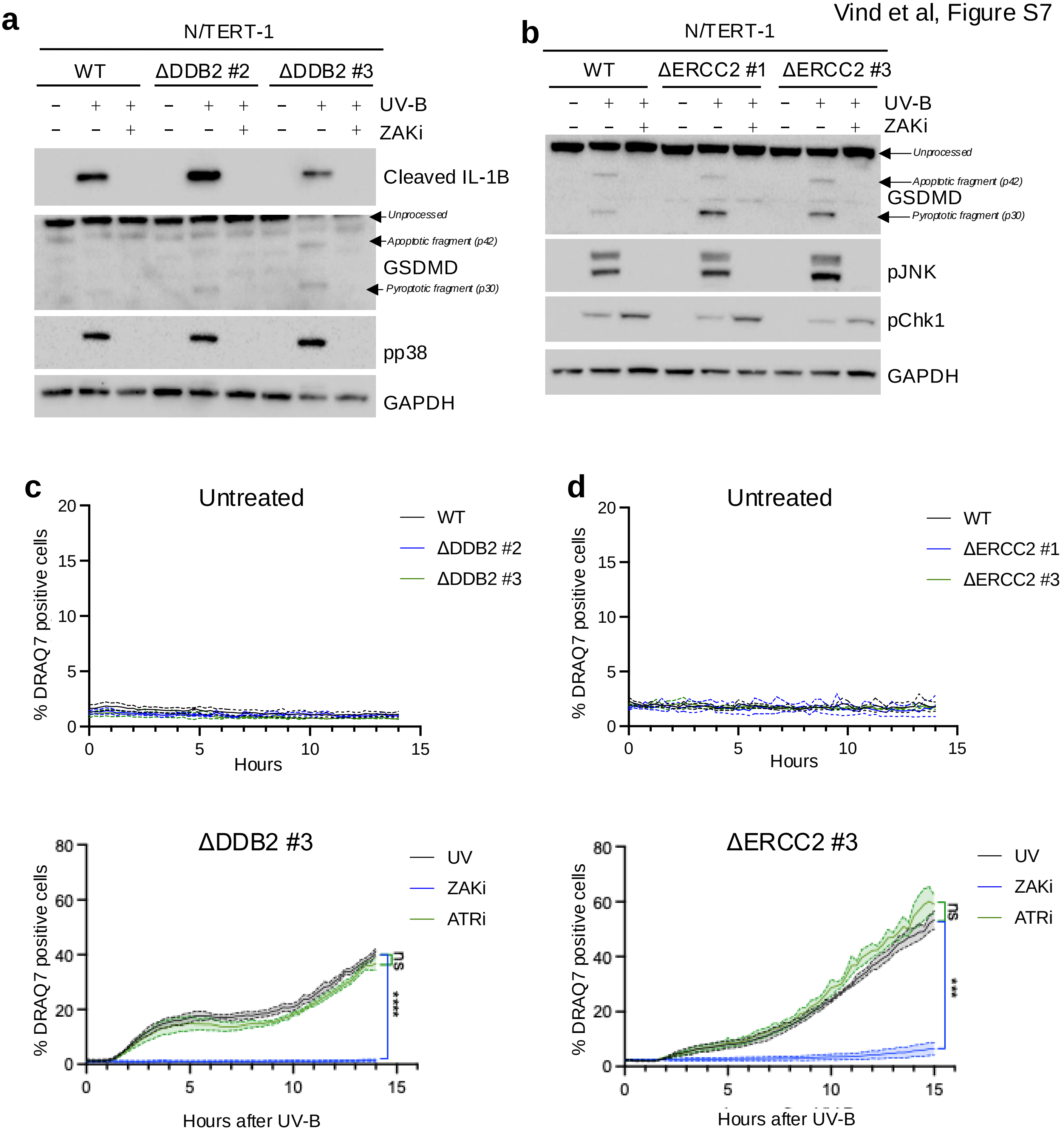

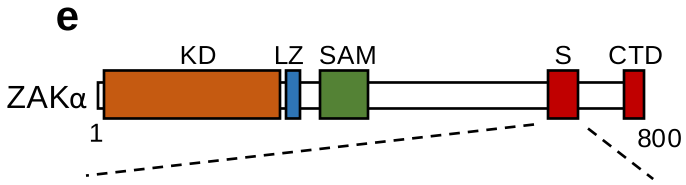

RGRYSD**R**SRNKYGRGSISLNSSP**RGRY**SG**K**SQ
HSTPS**RGRY**PG**K**

R/K→A: Arginine (R) and lysine (K) are mutated to alanine.

Figure S7.

The DNA damage response is dispensable for induction of early markers of UVB-induced pyroptosis and apoptosis (related to Fig. 6).

a) N/TERT-1 cells deleted for DDB2 (ΔDDB2) were treated with ZAK inhibitor (1 μM) and UVB-irradiated (100 J/m^2 – 8 h recovery). Lysates were analyzed by immunoblotting with the indicated antibodies. **b)** As in (a), except that cells were deleted for ERCC2 (ΔERCC2). **c)** WT and ΔDDB2 cells from (a) were grown in the presence of the live cell staining dye DRAQ7 and subjected to fluorescence time-lapse microscopy (top) or treated with indicated inhibitors (i, 1 μM), irradiated with UVB (100 J/m^2) and subjected to fluorescence time-lapse microscopy (bottom). **d)** As in (c), except that WT cells were compared to ΔERCC2 cells from (b). Statistical significance for pyroptosis was calculated by two-tailed Kolmogorov–Smirnov test at 95% confidence interval between 0-5 hrs. ns., non-significant; ***, $p<0.001$; ****, $p\leq0.0001$. **e)** Schematic of the ZAKα protein highlighting the three RGRYXXR/K motifs in the sensor (S) domain. These motifs are mutated to AGAYXXA in the R/K->A ZAKα mutants used in Fig. 6j. KD, Kinase Domain; LZ, Leucine Zipper; SAM, Sterile Alpha Motif; CTD, C-Terminal Domain. Percentage of dye-including cells are plotted as mean and error bars represent the standard error of the mean (SEM) for three technical replicates.
