# Supplementary material for: The ribotoxic stress response drives acute inflammation, cell death, and epidermal thickening in UV-irradiated skin in vivo
Source: Mol Cell. 2024 Dec 19;84(24):4774–4789.e9. doi: 10.1016/j.molcel.2024.10.044 (PMC11671030; doi:10.1016/j.molcel.2024.10.044)
Supplement: Document S2. Article plus supplemental information [file mmc4.pdf]

Molecular Cell

Article

The ribotoxic stress response drives acute inflammation, cell death, and epidermal thickening in UV-irradiated skin *in vivo*

Graphical abstract

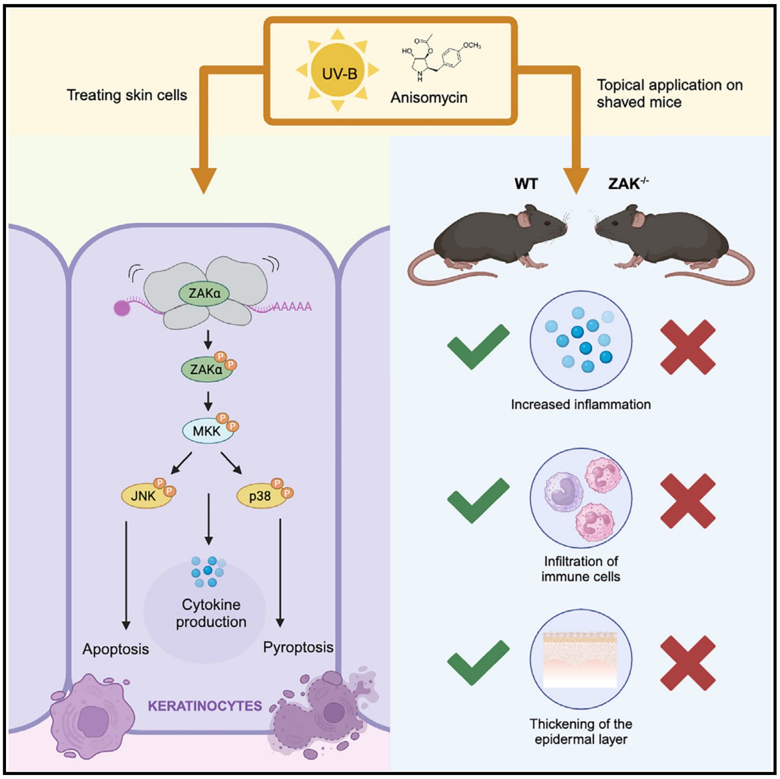

Highlights

- The ribotoxic stress response underlies acute outcomes of sunburn reactions in skin
- ZAKα and JNK mediate apoptosis in keratinocytes and common cell lines after UV
- ZAKα, p38, and NLRP1 mediate pyroptosis in human keratinocytes after UV
- Anisomycin on mouse ears triggers ZAKα-dependent swelling and inflammation

Authors

Anna Constance Vind, Zhenzhen Wu, Muhammad Jasrie Firdaus, ..., Mads Gyrd-Hansen, Franklin L. Zhong, Simon Bekker-Jensen

Correspondence

vind@sund.ku.dk (A.C.V.), franklin.zhong@ntu.edu.sg (F.L.Z.), sbj@sund.ku.dk (S.B.-J.)

In brief

The acute skin reaction to sunburn, encompassing keratinocyte cell death, inflammation, and epidermal thickening, has traditionally been ascribed to DNA damage responses. Using a mouse model deficient for the *Zak* gene, Vind et al. demonstrate that these reactions depend on sensing of cytoplasmic RNA damage rather than nuclear DNA damage.

Vind et al., 2024, Molecular Cell *84*, 4774–4789
December 19, 2024 © 2024 The Author(s). Published by Elsevier Inc.
https://doi.org/10.1016/j.molcel.2024.10.044

CellPress

CellPress
OPEN ACCESS

Molecular Cell

Article

The ribotoxic stress response drives acute inflammation, cell death, and epidermal thickening in UV-irradiated skin *in vivo*

Anna Constance Vind,[1,2,12,*] Zhenzhen Wu,[1,2,12] Muhammad Jasrie Firdaus,[3,12] Goda Snieckute,[1,2] Gee Ann Toh,[3] Malin Jessen,[4] José Francisco Martínez,[1,2] Peter Haahr,[2] Thomas Levin Andersen,[5,6] Melanie Blasius,[1,2] Li Fang Koh,[7] Nina Loeth Maartensson,[8,10] John E.A. Common,[7,9] Mads Gyrd-Hansen,[4] Franklin L. Zhong,[3,11,*] and Simon Bekker-Jensen[1,2,13,*]

[1]Center for Healthy Aging, Department of Cellular and Molecular Medicine, University of Copenhagen, Blegdamsvej 3, 2200 Copenhagen, Denmark
[2]Center for Gene Expression, Department of Cellular and Molecular Medicine, University of Copenhagen, Blegdamsvej 3, 2200 Copenhagen, Denmark
[3]Lee Kong Chian School of Medicine, Nanyang Technological University, 11 Mandalay Road, Singapore 308232, Singapore
[4]LEO Foundation Skin Immunology Research Center, Department of Immunology and Microbiology, University of Copenhagen, Blegdamsvej 3, 2200 Copenhagen, Denmark
[5]Clinical Cell Biology, Department of Pathology, Odense University Hospital, University of Southern Denmark, J.B.Winsløwsvej 25, 5000 Odense, Denmark
[6]Molecular Bone Histology (MBH) lab, Department of Clinical Research, University of Southern Denmark, J.B.Winsløwsvej 25, 5000 Odense, Denmark
[7]A*STAR Skin Research Labs (A*SRL), Agency for Science, Technology and Research (A*STAR), & Skin Research Institute of Singapore (SRIS), 8A Biomedical Grove, Singapore 138648, Singapore
[8]Department of Pathology, Diagnostic Center, Copenhagen University Hospital - Rigshospitalet, Copenhagen, Denmark
[9]Translational and Clinical Research Institute, Newcastle University, Newcastle upon Tyne, UK
[10]Department of Clinical Medicine, University of Copenhagen, Copenhagen, Denmark
[11]Skin Research Institute of Singapore (SRIS), #17-01 Clinical Sciences Building, 11 Mandalay Road, Singapore 308232, Singapore
[12]These authors contributed equally
[13]Lead contact
*Correspondence: vind@sund.ku.dk (A.C.V.), franklin.zhong@ntu.edu.sg (F.L.Z.), sbj@sund.ku.dk (S.B.-J.)
https://doi.org/10.1016/j.molcel.2024.10.044

SUMMARY

Solar UVB light causes damage to the outermost layer of skin. This insult induces rapid local responses, such as dermal inflammation, keratinocyte cell death, and epidermal thickening, all of which have traditionally been associated with DNA damage response signaling. Another stress response that is activated by UVB light is the ribotoxic stress response (RSR), which depends on the ribosome-associated mitogen-activated protein 3 kinases (MAP3K) ZAKα and culminates in p38 and JNK activation. Using ZAK knockout mice, we here show that it is the RSR that is responsible for the early manifestation of UVB-induced skin inflammation and keratinocyte death and subsequent proliferation *in vivo*. We also show that the RSR controls both p38-mediated pyroptotic and JNK-mediated apoptotic programmed cell death of human keratinocytes *in vitro*. In sum, our work highlights that skin cells rely on a cytoplasmic and ribosomal stress signal rather than a nuclear and DNA-templated signal for rapid inflammatory responses to UV exposure.

INTRODUCTION

Skin separates the organism from its surroundings and provides a barrier against the external environment, trauma, and infection. In this capacity, the skin is also the most exposed part of our body and is constantly challenged with radiation, chemical irritants, and thermal fluctuations. Of these, solar ultraviolet (UV) radiation is an especially prevalent threat to the integrity of skin cells, given its well-established DNA damaging and consequently carcinogenic effects.[1] A range of cellular stress insults, including osmotic shock, heat, reactive oxygen species (ROS), mechanical perturbation, UV irradiation, and inflammatory stimuli, activate the stress-associated mitogen-activated protein (MAP) kinases (MAPKs) p38 and JNK.[2] These reactions typically require signaling through upstream MAP2 kinases (MAP2Ks) and MAP3 kinases (MAP3Ks), with the latter being the proximal stress-sensing or ligand-binding component.[3,4] p38 signaling mediates inflammatory signaling through the production of

 © 2024 The Author(s). Published by Elsevier Inc.
This is an open access article under the CC BY license (http://creativecommons.org/licenses/by/4.0/).

CellPress
OPEN ACCESS

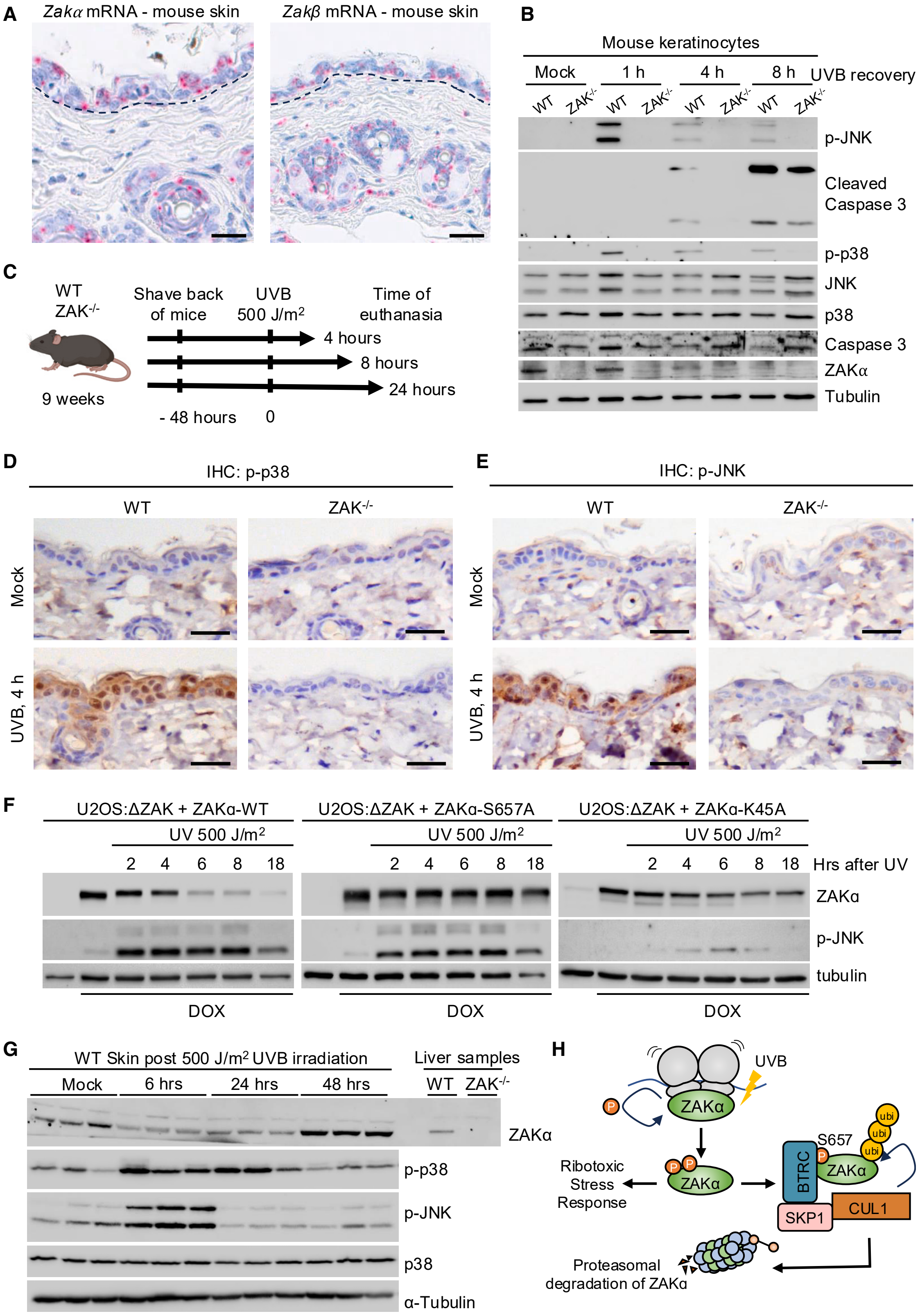

(legend on next page)

cytokines and cell fate decisions such as cell death, cell-cycle arrest and cell differentiation.[5] JNK signaling on the other hand has largely been linked to the induction of apoptotic cell death.[3] Among the diverse group of 21 human p38 and JNK-directed MAP3Ks, ZAKα (MAP3K20) has recently attracted attention as the central regulator of the ribotoxic stress response (RSR). ZAKα senses translational aberrations by virtue of its two C-terminal ribosome-binding domains[6] and triggers p38 and JNK signaling.[7] Known ZAKα-activating molecules include ribotoxin enzymes (including ricin, Shiga toxin, and α-sarcin), bacterial/fungal metabolites (including anisomycin and cycloheximide), and UV irradiation, which either damage the ribosomal RNA, chemically inhibit the ribosome, or damage messenger RNA (mRNA) templates.[8,9] ZAKα activation is elicited by both collision[10] (strong) and stalling[6] (weak) of ribosomes through as-of-yet poorly understood mechanisms. More recently, amino acid starvation and nitric oxide have also been shown to activate the RSR by promoting the formation of ribosome stalling and/or collisions.[11,12]

UV irradiation crosslinks mRNA nucleotides[13] as well as DNA nucleotides, and this is associated with stalling and eventual collisions of ribosomes on the damaged mRNA templates.[10,14] Consequently, UV irradiation has been shown to activate ZAKα and RSR signaling *in vitro*[9,15,16] but with unknown physiological ramifications. Recently, the human inflammasome sensor NLRP1 was shown to be a target for direct phosphorylation and activation by ZAKα as well as p38.[17,18] In human keratinocytes *in vitro*, a pathway leading from ZAKα to NLRP1-dependent pyroptosis was shown to underlie rapid pyroptotic cell death induced by UV light and other ribotoxic insults.[17,19] Curiously, the murine NLRP1 homologs (a–c) are refractory to p38-mediated activation.[17] Thus, we previously proposed that NLRP1-driven pyroptosis has evolved to form a special, ancillary arm of the RSR specifically in human NLRP1-expressing cell types such as keratinocytes, whereas the core RSR response, including apoptosis induction and the upregulation of pro-inflammatory transcripts, is common to most cell types and conserved in mice.[19] However, the importance of ZAKα in defending against physiologically relevant, ubiquitously present ribotoxic insults, such as UVB, has not been rigorously characterized *in vivo*, especially in the most relevant organ and cell type, i.e., the skin and keratinocytes.

Acute sun exposure is associated with sunburn, a skin reaction that manifests locally as pain, itching, blistering, and wounding.[20] The underlying causes of these reactions are epidermal cell death and dermal inflammation, resulting from either cytokines and chemokines or immune reactions against cellular contents released by dead cells. Epidermal damage caused by UV irradiation is also associated with hyperproliferation of keratinocytes.[21] This is a compensatory reaction to keratinocyte cell death but can produce adverse effects on tissue homeostasis, resulting in a marked thickening of the keratinocyte cell layer. The vast majority of scientific and lay literature on the subject points to UV-induced DNA damage signaling as the cause of the detrimental consequences of UV exposure.[22–25] However, a direct link between DNA damage signaling and the acute inflammatory and cell death responses in the skin have not been firmly established *in vivo*. Here, we show that it is the ZAKα-driven RSR rather than the DNA damage response (DDR) that governs immediate skin reactions to UVB irradiation *in vivo*, including programmed cell death and manifestation of inflammation. Consequently, *Zak* deletion provides marked protection against both p38-induced pyroptosis and JNK-dependent apoptosis in human keratinocytes *in vitro* and early skin inflammation and epidermal thickening in mice. As further evidence, we demonstrate that skin-specific RSR induction via topical application of a tool compound, anisomycin, is sufficient to elicit similar dermal inflammation and epidermal thickening in wild-type (WT) but not ZAK$^{-/-}$ mice. Our work offers important insight into the mechanisms underlying acute skin responses to UV exposure and highlights RSR as a critical physiological regulator of skin inflammation *in vivo*.

RESULTS

The ribotoxic stress response underlies UVB-induced p38 and JNK activation in mouse skin

Using RNAScope-based *in situ* hybridization,[26] we demonstrated that both α and β splice forms of the *Zak* gene are enriched in the epidermal layer of mouse skin (keratinocytes) (Figure 1A), whereas only the β isoform was detected in the muscle fibers beneath the hypodermis (Figures S1A and S1B). The latter is consistent with previous expression analyses for the splice variants of this gene in humans and mice.[11,27] We proceeded to isolate and cultivate primary tail keratinocytes from WT and ZAK$^{-/-}$ mice that we exposed to 500 J/m^2 of UVB irradiation. In these cells, p38 and JNK activation was completely dependent on the *Zak* gene and was

Figure 1. The ribotoxic stress response is activated in mouse skin by UVB irradiation

(A) Paraffin-embedded mouse skin was stained for *Zakα* (left) and *Zakβ* (right) mRNA (pink dots) by *in situ* hybridization with a modified RNAScope protocol. The dashed line demarcates the epidermis from the dermis.

(B) Keratinocytes isolated from tail skin of WT and ZAK$^{-/-}$ mice were UVB irradiated (500 J/m^2) and left to recover for the indicated times. Lysates were analyzed by immunoblotting with the indicated antibodies.

(C) Schematic of *in vivo* mouse UVB-irradiation experiments. 9-week-old female WT and ZAK$^{-/-}$ mice were shaved on their backs and their skin was depilated 48 h prior to UVB exposure (500 J/m^2). Different times of euthanasia and skin harvesting are indicated.

(D) Skins of mice from (C) were harvested 4 h after irradiation and analyzed by immunohistochemistry for activated p38 (p-p38).

(E) As in (D), except that p-JNK antibody was used.

(F) U2OS cells deleted for ZAK (ΔZAK) were stably rescued with doxycycline (Dox)-inducible WT and mutated forms of ZAKα. Cells were UVB irradiated (500 J/m^2) and left to recover for the indicated times. Lysates were analyzed as in (B).

(G) Full-thickness skin of WT mice irradiated as in (C) was lysed and analyzed as in (B). Liver lysates from WT and ZAK$^{-/-}$ mice served as a control for antibody specificity.

(H) Model of activation-associated ZAKα degradation by the SCF$^{\beta\text{-Trcp}}$ ubiquitin ligase and the proteasome. All scale bars, 50 μm. See also Figure S1.

CellPress
OPEN ACCESS

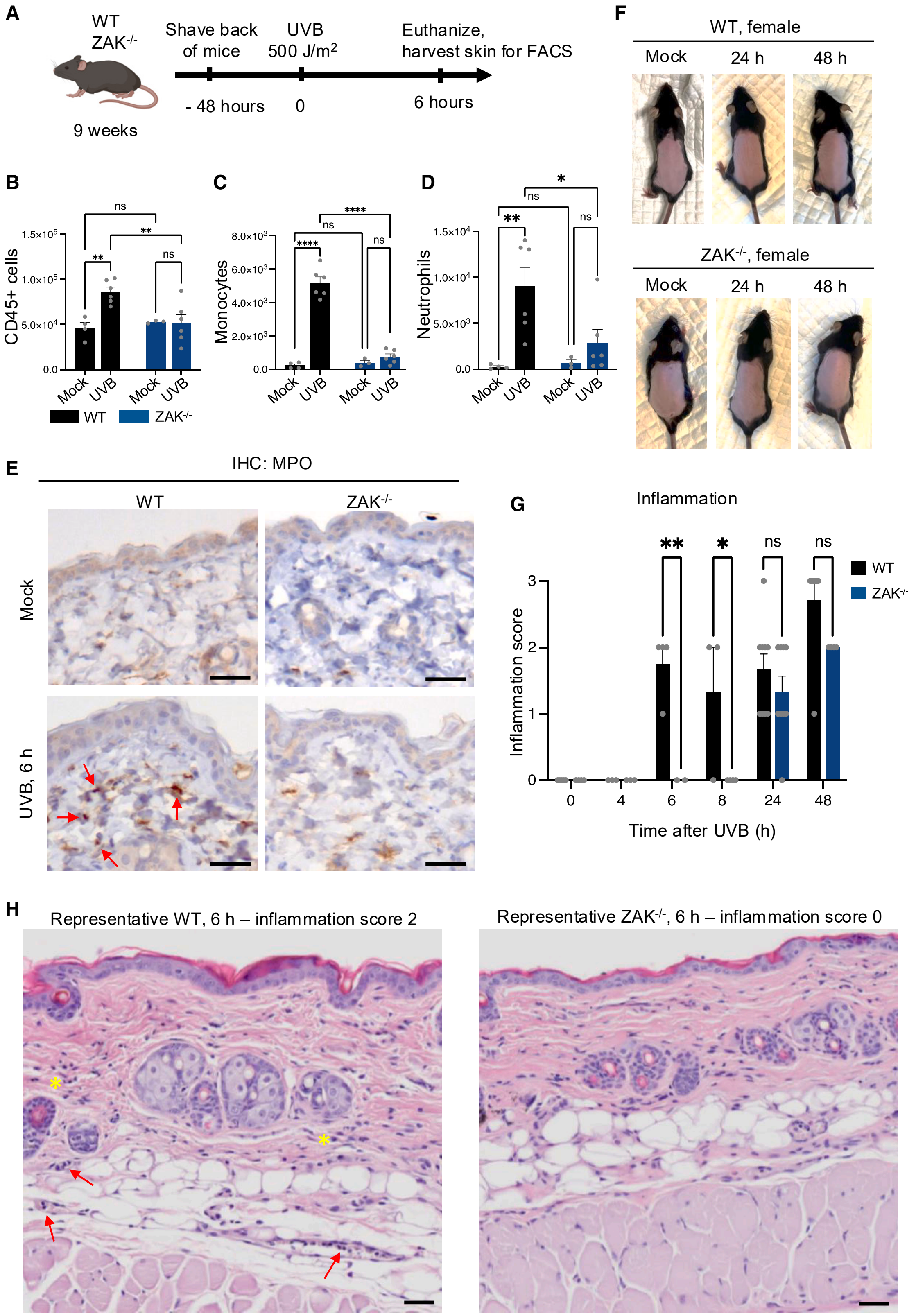

(legend on next page)

accompanied by early ZAKα-dependent apoptosis, as indicated by cleavage of caspase-3 (Figure 1B). We next subjected shaved and depilated back skin of WT and ZAK$^{-/-}$ mice to a similar regimen of UVB irradiation *in vivo* (Figure 1C). Immunohistochemical (IHC) analysis for the activated forms of p38 and JNK (p-p38, p-JNK) confirmed our *in vitro* observations with primary keratinocytes and indicated that the ZAKα kinase is obligatory for the early UVB-induced activation of these kinases (4-h time point) (Figures 1D and 1E). IHC analysis for cleaved caspase-3 indicated reduced keratinocyte apoptosis at the 8-h time point but not at the 24-h time point (Figure S1C). However, the number of TUNEL-positive cells was indistinguishable between genotypes and much higher than for cells positive for cleaved caspase-3 at both time points (Figure S1D). Thus, *in vivo*, only a subset of dying cells display a classical marker of apoptosis (cleaved caspase-3), whereas cells positive for a marker of digested or degraded DNA (TUNEL) are rampant irrespective of genotype. Our results suggest that cell death, regardless of the underlying mechanism, is not generally repressed by *Zak* deletion in UVB-irradiated mouse skin.

In primary mouse keratinocytes, the activation of JNK and p38 was transient, peaking at 1 h and gradually disappearing at later time points. This coincided with the apparent degradation of the ZAKα kinase (Figure 1B). We previously described an autophosphorylation-dependent establishment of a β-TrCP degron in ZAKα,[6,16] and we considered that this event could be responsible for destabilization of the RSR-activating kinase upon UVB irradiation. Indeed, whereas WT S-hemagglutinin (HA)-tagged ZAKα was rapidly degraded in UVB-irradiated U2OS cells, kinase-dead as well as a single-point mutant of ZAKα that is refractory to auto-phosphorylation of the β-TrCP degron remained stable (Figure 1F). Importantly, we observed a similar UVB-induced destabilization of ZAKα and transiency of p38 and JNK activity *in vivo* (full thickness skin), albeit with somewhat delayed kinetics (Figure 1G). We posit that UVB-induced ZAKα degradation, governed by autophosphorylation and β-TrCP recognition, provides for a negative feedback loop that both acts to terminate stress signaling and likely render the skin refractory for consecutive rounds of RSR activation (Figure 1H). Similar conclusions were recently reached by others.[16]

ZAK$^{-/-}$ mice are protected against early skin inflammation after UVB-irradiation

Next, we performed fluorescence-activated cell sorting (FACS)-based "immunophenotyping" of isolated cells from the skin of mice euthanized 6 h after UVB irradiation (Figure 2A). Our gating strategy allowed us to isolate and quantify populations of relevant myeloid cells and lymphocytes (Figures S2A and S2B). This approach revealed a UVB-induced and completely *Zak*-dependent recruitment of CD45-positive myeloid cells (Figure 2B). A deeper analysis of the underlying cell populations highlighted that this increase could be largely ascribed to monocytes and neutrophils (Figures 2C and 2D), the immune cells that have previously been shown to mediate early inflammatory responses to acute sunburn.[28,29] Langerhans cells were also modestly increased in the skin, but no other types of immune effector cells had significantly infiltrated at this early time point (Figure S2C). We also analyzed dermal neutrophilic inflammation by IHC detection of cells positive for myeloperoxidase (MPO). MPO staining clearly increased in UVB-irradiated skin from WT mice but not from ZAK$^{-/-}$ mice (Figure 2E). Importantly, these effects, i.e., cell death and immune cell infiltration in the skin, occur rapidly—within hours following UVB irradiation—and are not associated with any gross macroscopic destruction of the tissue, as indicated by the complete lack of wounding, edema and/or scaling at least up till 48 h after irradiation (Figures 2F; Figure S3A). Finally, we subjected all of our hematoxylin and eosin (H&E) stained skin sections to a detailed histopathological analysis and used clinical-pathology-based criteria to assess the severity of inflammation (Figures 2G and 2H; Figure S3B). Strikingly, ZAK$^{-/-}$ mice, in contrast to WT mice, did not display any histological signs of dermal or intraluminal inflammation at early time points (6 and 8 h) (Figures 2G and 2H) but did show increased skin inflammation at 24- and 48-h time points (Figure 2G). We also analyzed the same H&E-stained slides for morphological characteristics associated with cell death processes in skin keratinocytes. In both WT and ZAK$^{-/-}$ mice, we readily noticed dyskeratotic keratinocyte nuclei and acanthosis consistent with apoptotic and necrotic cell death (Figures S3C and S4A). Thus, in mouse skin where NLRP1 orthologs do not support rapid ZAKα-p38-dependent pyroptotic cell death,[17] ablation of the RSR limits acute inflammation but is not sufficient to prevent delayed UVB-induced cell death.

Figure 2. ZAK$^{-/-}$ mice are protected against early immune cell infiltration in UVB-irradiated skin

(A) Schematic of *in vivo* mouse UVB-irradiation experiment for fluorescence-activated cell sorting (FACS)-based analysis of skin-infiltrating immune cells. 9-week-old female WT and ZAK$^{-/-}$ mice were shaved on their backs and their skin was depilated 48 h prior to UVB exposure (500 J/m^2). Mice were euthanized and back skin was harvested 6 h after irradiation.

(B–D) Mouse skin samples were collected from control or UVB-irradiated mice and immune infiltrates were analyzed by flow cytometry. Graphs represent the mean number of (B) total immune cells (CD45$^+$), (C) monocytes (CD11b$^+$ Ly-6C$^+$), and (D) neutrophils (CD11b$^+$ Ly-6G$^+$). The data were obtained from two independent experiments with 3–7 mice per group. Data are presented as mean, with error bars denoting the standard error of the mean (SEM). ns., non-significant; *$p \leq 0.05$; **$p \leq 0.01$; ***$p < 0.001$; ****$p \leq 0.0001$ using two-way ANOVA with the Sidak method.

(E) Skin sections from (A) were analyzed by immunohistochemistry for myeloperoxidase (MPO) expression. Red arrows indicate MPO-positive immune cells.

(F) Representative images of female mice 24 and 48 h after UVB irradiation (500 J/m^2).

(G) Severity of skin inflammation of 9-week-old female WT and ZAK$^{-/-}$ mice at 6, 8, 24, and 48 h after UVB irradiation (500 J/m^2) was assessed histologically based on infiltrating immune cells according to the following scoring system: normal, 0; intraluminal infiltration, 1; intraluminal + light dermal infiltration, 2; intraluminal + moderate dermal infiltration, 3. Data are plotted as mean and all error bars represent the standard error of the mean (SEM) (n = 3–9 biological replicates). ns., non-significant; *$p \leq 0.05$; **$p \leq 0.01$; in two-way ANOVA with the Sidak method.

(H) Hematoxylin and eosin (H&E)-stained skin sections from (A). Red arrows indicate intraluminal infiltration and yellow asterisks indicate dermal infiltration of immune cells. All scale bars, 50 μm. See also Figures S1–S3.

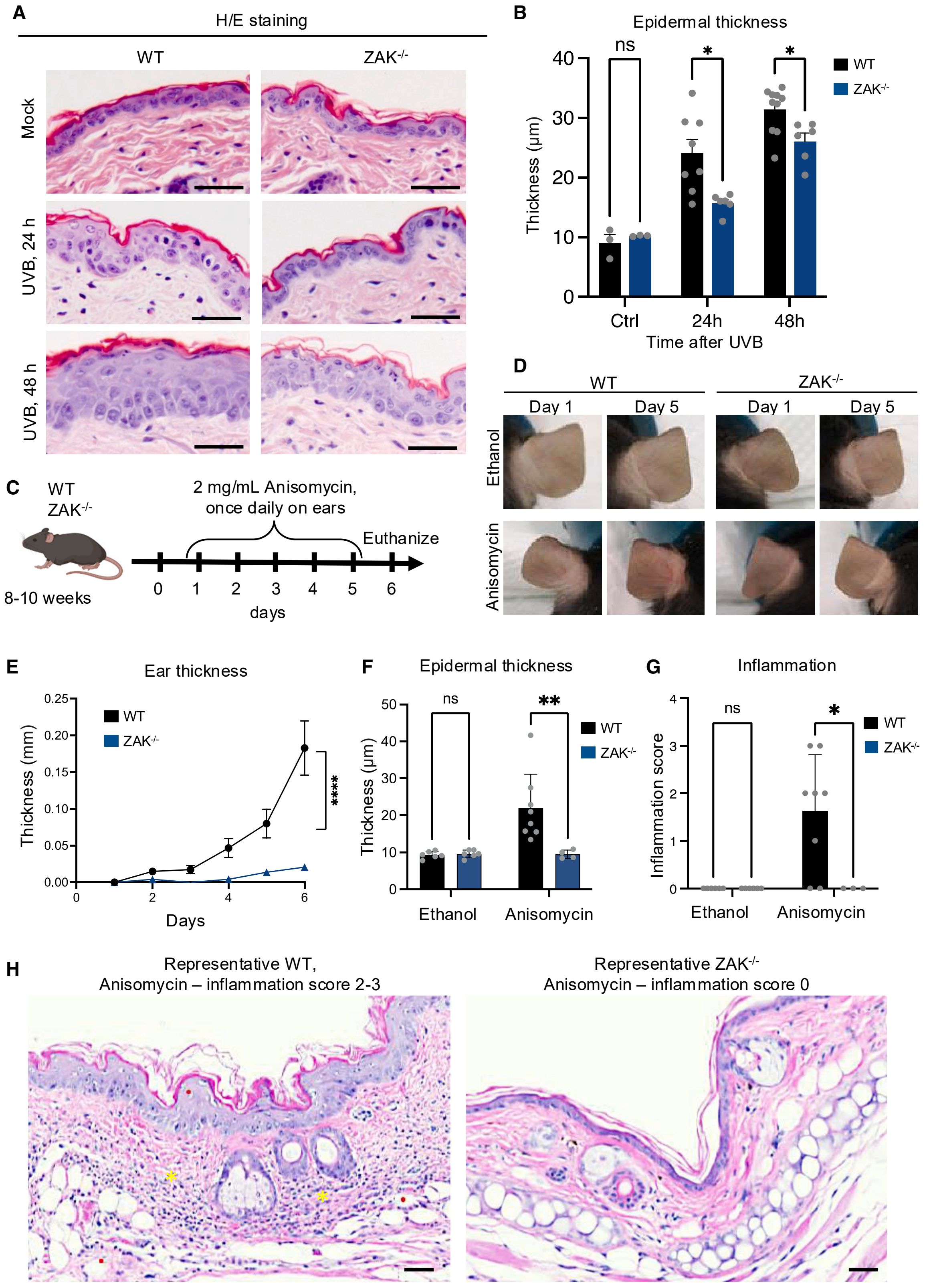

(legend on next page)

UVB-induced epidermal thickening is fueled by the ribotoxic stress response

In vivo, keratinocyte cell death is compensated for by increased proliferation of intact keratinocytes, a process which over time can result in a gradual thickening of the epidermal cell layer.[21] Although WT mice presented with a more-than-3-fold thicker epidermis at 24 and 48 h after UVB exposure, this response was significantly blunted in the skin of ZAK$^{-/-}$ mice (Figures 3A and 3B). The underlying mechanisms that support this increased proliferation likely involve a network of paracrine factors (such as epidermal growth factor [EGF], HBEGF, KGF, and interleukin [IL]-22[30]) among multiple cell types as shown previously.[21] Overall, our *in vivo* studies highlight that ZAK$^{-/-}$ mouse skin is deficient in acute reactions toward a single dose of UVB irradiation. These include activation of stress-associated MAP kinases (SAPKs), inflammatory responses, and thickening of the epidermal cell compartment (Figure S4B).

These results, although confirming a role of ZAK in UV response *in vivo*, cannot distinguish the effects of DNA damage vs. mRNA damage upstream of ZAK activation. Thus, we asked whether triggering RSR by itself, in the absence of direct DNA damage, is sufficient to induce any of these histological features. To this end, we topically applied anisomycin dissolved in ethanol to the ears of mice for 5 consecutive days (Figure 3C). In WT mice, this procedure induced a marked swelling of the ear (Figure 3D) and thickening of the skin compartment (measured with a caliper) (Figure 3E; Figure S4C). Histological examination of the tissues also revealed both epidermal thickening and marked inflammation (Figures 3F–3H), however, in the complete absence of cell death. Strikingly, these pathological features were suppressed in the skin of ZAK$^{-/-}$ mice (Figures 3D–3H), echoing our findings in UVB-irradiated skin. Topical application of anisomycin has previously been used as a chemically induced murine model of dermatitis that resembles human psoriasis,[31] and previous work showed that a p38 kinase inhibitor could suppress psoriatic features in this model.[32]

Next, we employed two additional models to investigate whether ZAK controls skin inflammation specifically downstream of RSR triggers or is more generally involved in other types of skin inflammation. In the first model, we applied imiquimod (IMQ) in Vaseline cream to shaved back skin of mice for 5 consecutive days (Figure S4D) to induce psoriasis-like dermatitis. IMQ is thought to act as an agonist for Toll-like receptor (TLR) 7 in dermal immune cells and lead to T helper (Th)17-driven skin inflammation.[31] As expected, we observed marked histological changes in IMQ-treated mice relative to Vaseline-treated control mice, as evidenced by massive skin inflammation and epidermal thickening (Figures S4E and S4F). In a second model, we applied another chemical, MC903, to mouse ears for 15 consecutive days (Figure S4G). MC903 is a vitamin D3 analog that induces atopic, dermatitis-like disease features via Th2-dominant inflammation.[33] MC903 induced significant scaling and barrier disruption, accompanied by marked histological signs of inflammation (Figures S4H and S4I). Contrary to the anisomycin and UVB model, ZAK$^{-/-}$ mice did not display any protection against pathological features in these two models of common inflammatory skin disease (Figures S4E, S4F, S4H, and S4I). As neither IMQ nor MC903 is known to induce RSR signaling, these results suggest that ZAKα is not a nonselective stress sensor but rather is highly specific to bona fide RSR inducers such as UVB and anisomycin in the context of skin inflammation.

ZAKα-driven pyroptosis and apoptosis occur via distinct pathways and can be genetically uncoupled in human keratinocytes

Similar to mice (Figure 1A; Figures S1A and S1B), the *Zak* gene is expressed in human skin. In fact, isoform-resolved human expression data from gtexportal.org highlight that skin is the human organ with the highest expression of *Zakα* and one of very few organs where *Zakα* is more abundant than the *Zakβ* splice form. IHC analysis of a human skin biopsy with two different antibodies confirmed the presence of ZAKα protein in both keratinocytes and skin fibroblasts (Figure 4A; Figure S5A) (of note, these antibodies do not react with mouse ZAKα). Similarly, RNAScope analysis revealed the presence of *Zakα* and *Zakβ* transcripts in human epidermis (Figures S5B and S5C). In primary human keratinocytes, UVB-induced activation of p38 and JNK, as well as an apparent phosphorylation-induced gel mobility shift and partial degradation of ZAKα, were completely abrogated by a ZAK kinase inhibitor (Figure 4B). However, in stark contrast to mouse keratinocytes, UVB irradiation of human keratinocytes also causes an inflammatory mode of programmed cell death, known as pyroptosis, which requires both ZAKα and the non-conserved NLRP1 inflammasome.[17] To understand this further, we dissected the genetic requirements for ZAKα and

Figure 3. The ribotoxic stress response drives epidermal thickening in mouse skin

(A) Representative H&E-stained images of UVB-irradiated skin (500 J/m^2) harvested from 9-week-old female WT and ZAK$^{-/-}$ mice after 24 and 48 h of recovery. Notice the attenuated thickening of the epithelial cell layer in ZAK$^{-/-}$ mice.

(B) Quantification of (A)—average thickness of the epidermal cell layer.

(C) Schematic of *in vivo* mouse anisomycin exposure experiment. Anisomycin was dissolved in ethanol (vehicle—2 mg/mL) and applied to one ear of 8- to 10-week-old female WT and ZAK$^{-/-}$ mice for 5 consecutive days. Pure ethanol was similarly applied to the other ear and mice were euthanized on day 6.

(D) Appearance of mouse ears subjected to the protocol in (C) before euthanasia. Notice the inflamed and irritated appearance of the WT ear after 5 days of anisomycin application.

(E) Growth in ear thickness of mice from (C) was measured with a caliper. Only data from anisomycin-treated ears are shown. Data are plotted as mean and all error bars represent the standard error of the mean (SEM). ****$p \leq 0.0001$; in one-way ANOVA at the 6-day time point.

(F) Epidermal cell layer thickness of ears from (C) were measured as in (B).

(G) Severity of skin inflammation in ears from (B) was assessed histologically based on infiltrating immune cells according to the scoring system described in the legend of Figure 2G.

(H) Representative H&E-stained images of ears from (C). All data are plotted as mean and all error bars represent the standard error of the mean (SEM) (n = 3–10 biological replicates). ns., non-significant; *$p \leq 0.05$; in two-way ANOVA with the Sidak method. All scale bars, 50 μm. See also Figure S4.

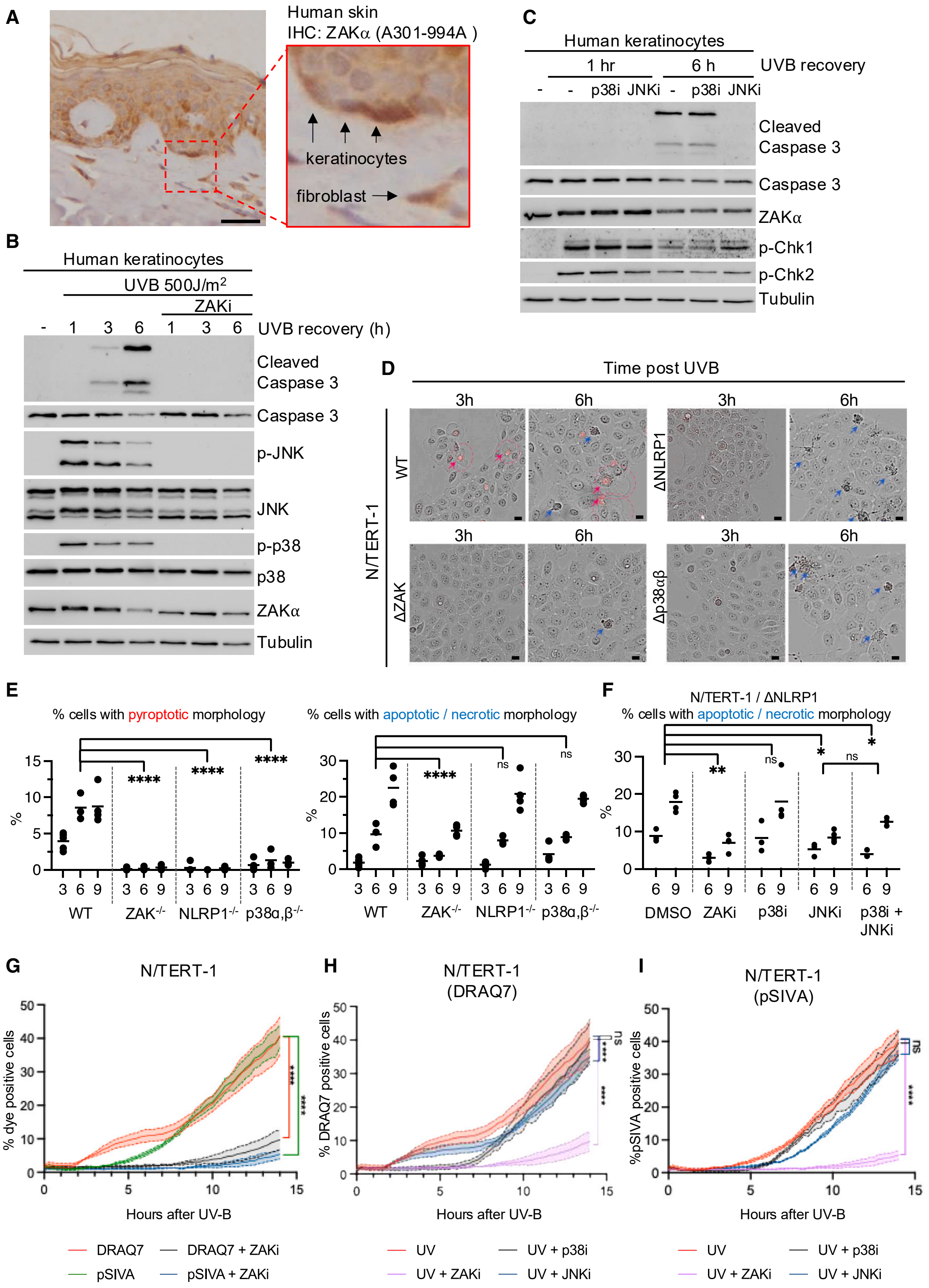

(legend on next page)

downstream SAPKs in each cell death pathway downstream of UVB. In primary human keratinocytes, UVB not only caused pyroptosis, as shown before,[17] but also classical signs of apoptosis, such as caspase-3 cleavage, which was completely abrogated by ZAK inhibition (Figures 4B and 4C). However, in contrast to pyroptosis, which required p38 signaling,[17,18] UVB-triggered apoptosis was dependent on JNK signaling and largely unaffected by p38 inhibition (Figure 4C). To rule out any potential crosstalk between the two modes of cell death, we examined common cell lines, HAP1 and U2OS, which do not express components of the NLRP1 inflammasome and are therefore naturally unable to undergo UVB-induced pyroptosis (Figures S5D–S5F). In both cell types, *Zak* deletion also abrogated UVB-induced caspase-3 cleavage (Figures S5D–S5G). Thus, the RSR is responsible for apoptosis execution in response to UVB in cell types that are naturally devoid of the pyroptosis machinery.

Next, we used a panel of isogenic immortalized keratinocyte cells (N/TERT-1) to investigate how UVB triggers apoptosis vs. pyroptosis at the single-cell level. As reported previously, N/TERT-1 cells display highly distinguishable morphological changes during the course of cell death: pyroptotic cells show ballooning of the membranes, with a >2- to 3-fold increase in volume, whereas apoptotic cells shrivel and become "darker," likely due to degradation of intracellular content. Therefore, the modes of cell death can be easily tracked via live-cell imaging. Similar to previous findings, we found that pyroptosis occurs with faster kinetics and is the most obvious mode of cell death at early time points after UV irradiation (~3 h) (Figure 4D), which was absent in ΔZAK, Δp38αβ, and ΔNLRP1 cells. This was supported by the loss of membrane ballooning, DRAQ7 incorporation, and IL-1B secretion (Figures 4D, 4E, red arrows, S5H, and S5I). Apoptotic cells became readily detectable 6 h post UVB irradiation in WT, ΔNLRP1, and Δp38αβ N/TERT-1 cells (Figure 4D, upper panels, blue arrows) and their relative frequencies were similar (Figure 4E). In contrast, ΔZAK cells displayed significantly reduced frequencies of cells with apoptotic morphologies at all time points (Figures 4D and 4E). JNK inhibition, unlike p38 inhibition, also reduced the frequencies of apoptotic cells (Figures 4F; Figure S5J).

We also complemented the morphological characterization of cell death using a marker-based imaging assay: DRAQ7 influx was used to indicate rapidly permeabilized cells (pyroptosis) and pSIVA, an annexin-based probe that detects phosphatidylserine externalization, was used to detect apoptotic cells.[34] This direct comparison revealed a kinetic difference between UV-triggered pyroptosis and apoptosis, with the former process occurring as early as 2 h following UVB irradiation and dominating over apoptosis (for which we see the first signs after 4 h) until the 9-h time point (Figure 4G). Both cell death pathways were highly ZAKα dependent, whereas p38 inhibition only impeded pyroptosis and JNK inhibition only impeded apoptosis, respectively, albeit to a lower extent than ZAK inhibition (Figures 4H and 4I). Taken together, these results provide further proof that ZAKα is the central regulator that controls both pyroptosis and apoptosis following UVB irradiation.

The inflammatory signature of UVB-irradiated human keratinocytes is dependent on ZAKα and p38

UVB-induced skin inflammation is associated with upregulation of inflammatory and stress responsive transcripts in keratinocytes.[28,29] These include cytokines and chemokines, which act to stimulate the extravasation and activation of immune effector cells from the bloodstream. p38 kinases play an established and critical role in the transcriptional response to UVB.[5] To study the role for the RSR in UVB-induced changes to the transcriptome, we performed total RNA sequencing of WT and ΔZAK N/TERT-1 keratinocytes 6 h after irradiation (Figure 5A). Using a stringent cutoff (log fold change [logFC] > 2), we found 321 significantly upregulated transcripts in WT keratinocytes compared with 293 in ΔZAK keratinocytes (Figures 5B and 5C; Table S1). Remarkably, most of the UVB-inducible transcripts in WT cells demonstrated a much smaller increase in ΔZAK keratinocytes (change in logFC < 1; Figure 5D). For instance, the known RSR response genes *Dusp1* and *Pmaip1* were found to be highly induced in irradiated WT cells but not upregulated above the cutoff in ΔZAK cells (Figure 5D). Among the transcripts that were upregulated more than an order of magnitude ($\log_2$-transformed) in WT over ΔZAK keratinocytes, we spotted well-known cytokine and chemokine mRNAs such as the ones encoding IL-6, CXCL2, CXCL7, and IL-23A (Figure 5D). A Gene Ontology (GO) enrichment analysis highlighted ZAK-dependent terms relevant to induction of immune responses and programmed cell death pathways (Figure 5E). To validate our results and further study these transcriptional changes in the context of regulation downstream of the ZAKα kinase, we proceeded to study primary human keratinocyte cultures obtained from skin biopsies of three healthy volunteers. We pre-treated these cells with relevant kinase inhibitors and

Figure 4. UVB-induced apoptosis and pyroptosis is ZAK dependent in human keratinocytes

(A) Paraffin-embedded human skin was analyzed for ZAKα expression by immunohistochemistry. Scale bar, 50 μm.

(B) Primary human keratinocytes were UVB irradiated (500 J/m^2) in the presence of ZAK inhibitor (ZAKi, 1 μM) and harvested at indicated time points. Lysates were analyzed by immunoblotting with the indicated antibodies.

(C) as in (B), except that cells were treated with inhibitors (i, 2 μM) of p38 and JNK kinases.

(D) Representative bright-field microscopy images of morphology to distinguish pyroptosis (pink arrows) and apoptosis (blue arrows) in N/TERT-1 cells following UVB exposure (100 J/m^2) at the indicated time points. Scale bars, 10 μm.

(E) Quantification of the different types of cell death seen in (D). ns., non-significant; ****$p \leq 0.0001$; in one-way ANOVA.

(F) Quantification of apoptotic ΔNLRP1 N/TERT-1 cells treated with indicated inhibitors (i, 0.5 μM) and UVB irradiation (100 J/m^2). ns., non-significant; *$p \leq 0.05$; **$p \leq 0.01$; in one-way ANOVA.

(G–I) N/TERT-1 cells were grown in the presence of the live-cell-staining dyes DRAQ7 (permeabilized cells) and pSIVA (apoptosis marker). Cells were treated with indicated inhibitors (i, 1 μM), irradiated with UVB (100 J/m^2), and subjected to fluorescence time-lapse microscopy. Error bars are from 3 technical replicates that are representative of 2 independent repeats. Statistical significance was calculated by two-tailed Kolmogorov-Smirnov test. Pyroptosis by DRAQ7 curve between 0 and 5 h, and apoptosis by pSIVA curve between 5 and 14 h. ns., nonsignificant; ****$p \leq 0.0001$. See also Figure S5.

CellPress
OPEN ACCESS

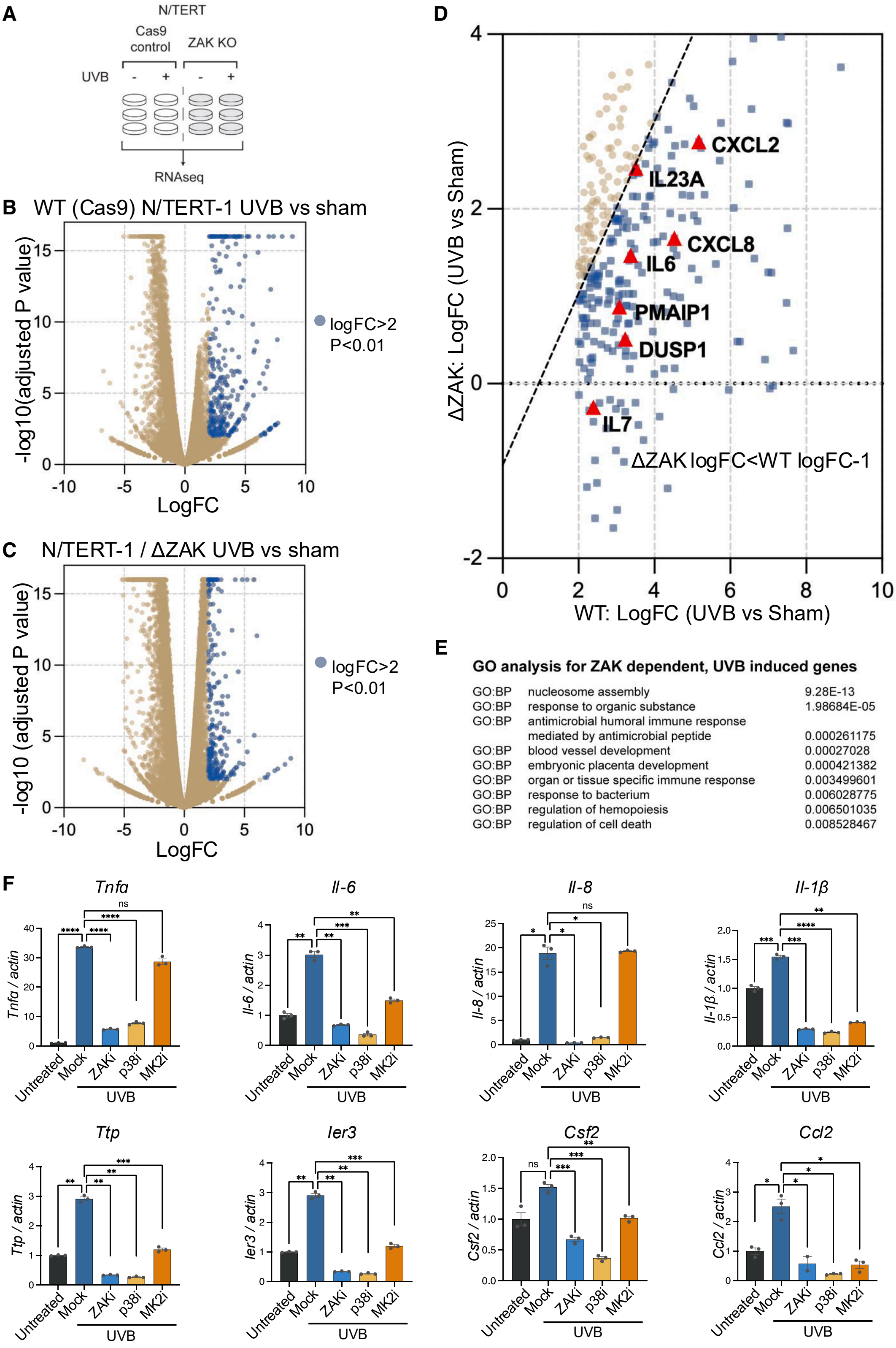

(legend on next page)

performed qPCR for selected transcripts 6 h after UVB irradiation. Indeed, transcripts encoding key immune modulators such as tumor necrosis factor alpha (TNF-α), IL-6, IL-8, IL-1β, CSF2, CCL2 (all cytokines and/or chemokines), and inflammation-associated transcripts for tristetraprolin (TTP) and IER3 were all consistently induced in both a ZAK- and p38-dependent manner (Figure 5F; Figures S5B–S5D). These responses were partly MK2 dependent as well. The RSR-dependent upregulation of *Ttp* is noteworthy, given its pervasive role in regulating the stability of inflammatory transcripts.[5,35] These results confirm that the ZAKα-driven RSR is responsible for most of the UVB-induced changes to the transcriptome and the induction of an inflammation-associated gene expression program.

UVB-induced inflammatory and cell-death signaling is independent of the DNA damage response *in vitro*

Given the prevalent notion in the field of genome integrity that UVB-induced skin inflammation and keratinocyte cell death are related to DNA crosslinks and other DNA photoproducts, we also investigated the role of the DDR in these processes. To this end, we first undertook a precise analysis of the kinetics of RSR and DDR signaling in N/TERT-1 cells upon UVB exposure. Both p38/JNK activation (strictly ZAKα dependent) and Chk1 activation (strictly ATR dependent) occurred very fast and with similar kinetics (Figure 6A). Second, we quantitatively measured the induction of the pro-inflammatory transcripts encoding IL-8, TNF-α, and TTP after UVB in primary human keratinocytes and found them all to be completely abrogated by ZAK inhibitor but not markedly changed by inclusion of an ATR inhibitor (Figure 6B). Third, we determined that biochemical markers of both pyroptosis (GSDMD1 pyroptotic cleavage fragment) and apoptosis (cleaved PARP1 and caspase-3) were present in both untreated and ATR-inhibited UVB-irradiated N/TERT-1 cells (Figure 6C). This stands in contrast to ZAKα-inhibited counterparts (Figure 6C), which showed neither apoptosis nor pyroptosis induction. Fourth, we employed our DRAQ7- and pSIVA-based live-cell-imaging system to assay the kinetics of pyroptotic and apoptotic cell death. Neither of these outcomes were affected by ATR signaling (Figure 6D), although we did observe a slight reduction in pyroptotic processing of GSDMD (Figure 6C) and a slight negative impact on the initial speed of pyroptotic cell death (Figure 6D), which we ascribe to potentially pleiotropic effects of the ATR inhibitor.

We also queried whether and how nucleotide excision repair (NER) regulates the choice of cell death mode in human skin keratinocytes. To do so, we used CRISPR-Cas9 to inactivate the most proximal sensors in two established sub-branches of NER: DDB2/XPE for global genome-NER (GG-NER) and ERCC2/XPD for transcription-coupled NER (TC-NER) in N/TERT-1 cells. It is worth noting that germline mutations in both genes cause xeroderma pigmentosum (XP), a group of congenital disorders characterized by extreme skin sensitivity to UV light.[36] Both ΔDDB2 and ΔERCC2 cells displayed biochemical signs of pyroptotic and apoptotic processing of the respective marker proteins GSDMD1/IL-1β and caspase-3/PARP1 (Figures 6E and 6F; Figures S7A and S7B). ΔDDB2 N/TERT-1 cells displayed accelerated DRAQ7 inclusion at early time points (<5 h) but no significant overall increase in pyroptosis or apoptosis at later time points. ΔERCC2 knockout (KO) cells did not differ appreciably from Cas9 control cells in either cell death mode after UVB irradiation. Most importantly, ZAK inhibition, but not ATR inhibition, ablated both pyroptosis and apoptosis in all cell types, irrespective of NER proficiency (Figures 6G–6I; Figures S7C and S7D). Together, these results demonstrate that it is the ZAKα-driven RSR rather than ATR-driven DDR or NER that drives UVB-induced cell death. Similar results were recently reported by another group.[16]

To exclude the possibility that ZAKα, an established cytoplasmic and ribosome-binding kinase,[37] could have a moonlighting function outside the RSR, we studied ΔZAK U2OS cells rescued with mutants of ZAKα that are deficient for ribosome interaction. We previously showed that this is mediated by two partially redundant C-terminal regions (S and CTD) that together are essential for ZAKα activation induced by ribosome inhibition[6,38] (Figure S7E). This was also the case for UVB irradiation, where individual mutation of the S and CTD domains had little or no effect on p38 and JNK activation but where the doubly mutated kinase was completely refractory for rescuing of the response (Figure 6J). We conclude from this that ZAKα requires a physical connection to stalled and/or collided ribosomes for UVB-induced activation.

In a recent publication, the Brummelkamp lab reported that the RNase SLFN11 is activated upon a range of genotoxic stressors, including UVB, and cleaves $tRNA^{Leu}$ (UAA). The depletion of the essential tRNA causes ribosomal arrest at UUA codons, RSR activation, and ribosome-templated ZAKα-JNK-dependent apoptosis.[39] These results suggest that the regulation *upstream* of RSR is more complex and nuanced than anticipated. We speculate that the SLFN11 pathway provides an alternative entry point to initiate ZAKα-driven RSR signaling (likely following sensing of single-stranded DNA [ssDNA] by SLFN11[40]) in addition to direct mRNA photo-damage that blocks translation. In support of the cited paper, we did find that ΔSLFN11 HAP1 cells were slightly protected against UVB-induced cell death at later time points but much less than

Figure 5. RNA sequencing reveals ZAK-dependent and UVB-induced transcriptional signatures in human keratinocytes
(A) Design of RNA sequencing (RNA-seq) experiment using indicated genotypes of N/TERT-1 cells.
(B) Significantly upregulated transcripts by UVB in WT/Cas9 control cells shown in blue.
(C) Significantly upregulated transcripts by UVB in ZAK KO/Cas9 control cells shown in blue.
(D) Correlation plot of UVB-induced transcripts in WT vs. ZAK KO cells. We selected transcripts that underwent a smaller $\log_2$-transformed fold change (logFC) in ZAK KO cells than in WT cells, as indicated by the diagonal at $y = x - 1$. Well-known chemokines and cytokines and cell-death-related genes are highlighted.
(E) Gene Ontology (GO) analysis of ZAK-dependent and UVB-induced transcripts.
(F) Primary human keratinocytes from one donor were UVB irradiated (500 J/m^2, 6 h) in the presence of inhibitors (i, 2 μM) against the kinases ZAK, p38, and JNK, as indicated. qPCR analysis of isolated RNA was performed using primers against the indicated transcripts. Fold changes are plotted as mean and all error bars represent the standard deviation (SD) (n = 3 technical replicates). ns., non-significant; *$p \leq 0.05$; **$p \leq 0.01$; ***$p < 0.001$; ****$p \leq 0.0001$; in two-way ANOVA with the Sidak method. See also Figure S6.

CellPress
OPEN ACCESS

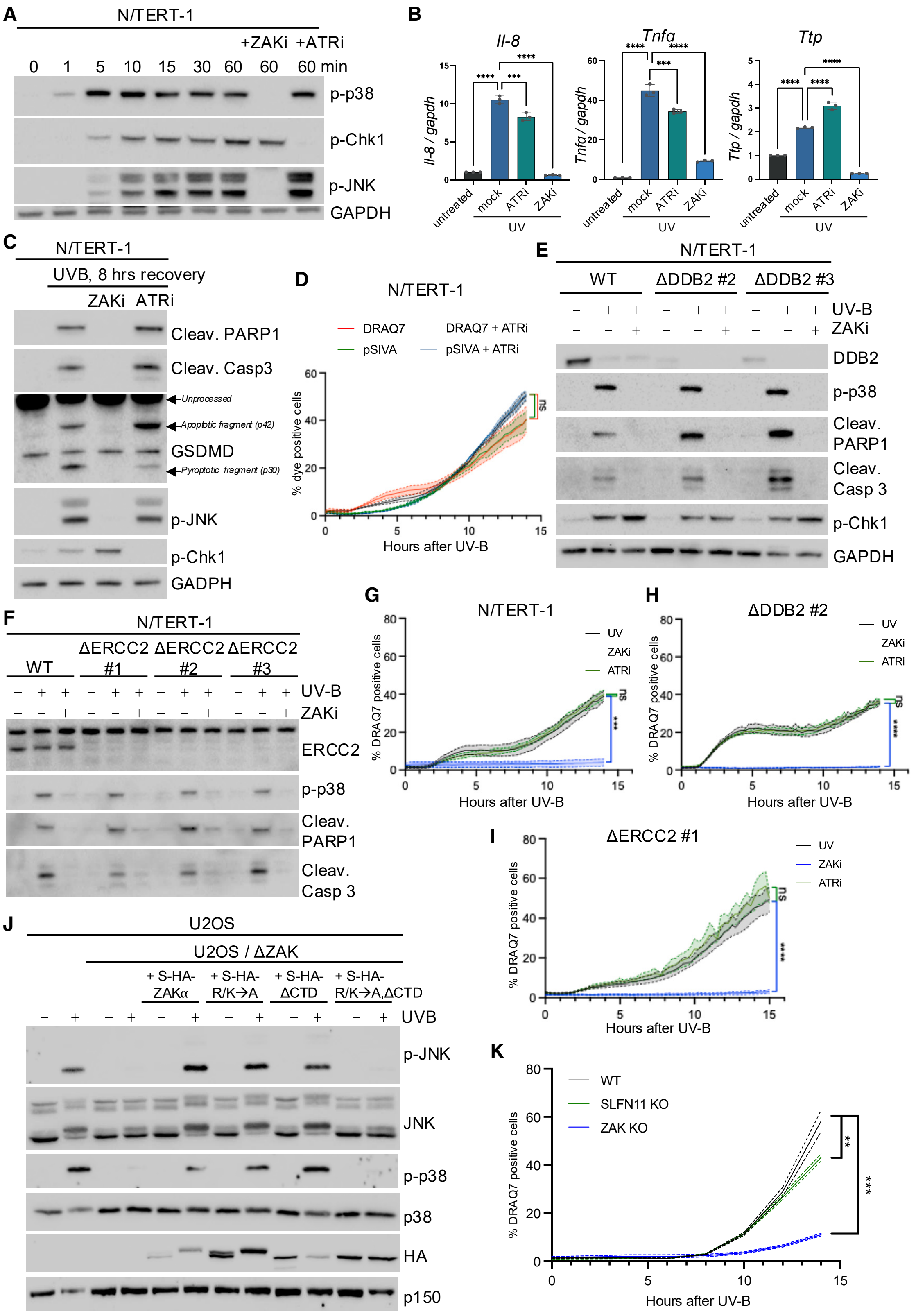

(legend on next page)

ΔZAK cells (Figure 6K). These findings reinforce the conclusion that the immediate cell death and inflammatory response triggered by UVB is predominantly driven by sensing of ribotoxic stress by ZAKα. Furthermore, the source of the ribotoxic stress mainly originates from ribosomes encountering cross-linked mRNA and, to a minor extent, DNA-damage-induced and SLFN11-mediated tRNA cleavage.

DISCUSSION

Here, we show that the RSR underlies acute responses to UVB irradiation in skin *in vivo*. These responses include the dermal infiltration of immune cells, programmed cell death responses, and epidermal thickening (Figure 7A). Another recent paper corroborates some of these findings and also establishes the RSR, and not the DDR, as the most important determinant for the induction of UV-induced apoptosis.[16] The surprising implications of our findings are that DDR is less important for these well-known responses than currently appreciated. When the pyroptotic machinery is absent, as in the case of mouse keratinocytes or NLRP1-deficient human keratinocytes, UVB-damaged cells die by ZAKα and JNK-dependent apoptosis *in vitro*. To clarify, we do not suggest that the ZAKα-driven pyroptosis and apoptosis are the only means by which cells can die. Even in the absence of both programmed cell death mechanisms, ZAKα-deficient human keratinocytes do eventually succumb to the damage accrued to their DNA and other macromolecular constituents. In mouse skin, where the pyroptosis inducer NLRP1 is not under the regulation of RSR, we did not observe an overall protection against UV-induced keratinocyte cell death (note that we cannot confidently distinguish the mode of cell death by IHC analysis). We speculate that the delayed inflammatory responses of ZAK$^{-/-}$ mice are caused by alternative innate immune reactions to debris and leaked content from dying cells.[41] This ZAKα-independent cell death may be the result of passive necrosis or rely on programmed pathways, potentially orchestrated by DDR signaling. It remains to be seen whether RSR inhibition would protect against acute cell death in human skin in a clinical setting. Contrary to apoptosis, which is considered to be noninflammatory,[42] pyroptotic cell death is associated with a nonspecific leakage of cellular content through gasdermin pores[43] as well as secretion of highly pyrogenic cytokines. The proinflammatory effect of UVB-induced ZAKα activation is thus likely to be even more pronounced in human skin, where the RSR can be assumed to underlie both proinflammatory pyroptotic cell death and production of cytokines and chemokines.

A further but delayed reaction to UVB irradiation and other stresses to the epidermis is the induced hyperproliferation of keratinocytes. The consequence is a thickening of the epidermal cell layer, and this event is also a hallmark of hyperproliferative and inflammatory skin conditions such as psoriasis.[44,45] The underlying mechanisms are unclear but likely involve keratinocyte-autonomous effects as well as paracrine signaling from immune cells. In this regard, it is worth noting that patients with gain-of-function mutations in NLRP1 and lowered threshold of pyroptosis suffer from nodular palmoplantar hyperplasia.[30] Although we did observe a gradual thickening of the epidermis in ZAK$^{-/-}$ mice after UV treatment, this reaction was clearly delayed and attenuated compared with WT mice (Figures 3A and 3B). Furthermore, our experiment using a direct RSR inducer, anisomycin, establishes that RSR signaling on its own is sufficient to induce marked dermal inflammation and epidermal thickening (Figures 3C–3H). In this light, it is intriguing that the majority of recently described human patients expressing *Zak* variants manifest with skin conditions of variable etiology, albeit among a number of unrelated phenotypes.[46] We only observed protection of ZAK$^{-/-}$ mice in the anisomycin mouse model of dermatitis, not two other models using IMQ and MC903 (Figures S4D–S4I), which are not known to induce RSR. It remains to be seen whether the RSR is activated in common human inflammatory skin diseases.

In summary, our work highlights ribosomes and ZAKα as the most proximal stress-signal-sensor pair that kickstarts the well-established acute skin reactions to acute UVB irradiation.

Figure 6. The DNA damage response does not impact on early UVB-induced keratinocyte cell death

(A) N/TERT-1 cells were treated with ZAK and ATR inhibitors (i, 1 μM), UVB irradiated (100 J/m^2), and lysed at the indicated time points. Lysates were analyzed by immunoblotting with the indicated antibodies.

(B) Primary human keratinocytes from one donor were UVB irradiated (500 J/m^2, 6 h) in the presence of ZAK and ATR inhibitors (i, 2 μM), as indicated. qPCR analysis of isolated RNA was performed using primers against the indicated transcripts. Fold changes are plotted as mean and all error bars represent the standard deviation (SD) (n = 3 technical replicates). ***p < 0.001; ****p ≤ 0.0001; in two-way ANOVA with the Sidak method.

(C) N/TERT-1 cells were treated as in (A) and lysed after 8 h. Lysates were analyzed by immunoblotting with the indicated antibodies.

(D) N/TERT-1 cells were grown in the presence of the live-cell-staining dyes DRAQ7 (permeabilized cells) and pSIVA (apoptosis marker). Cells were treated with ATR inhibitor (i, 1 μM), irradiated with UVB (100 J/m^2), and subjected to fluorescence time-lapse microscopy. Statistical significance was calculated by two-tailed Kolmogorov-Smirnov test at 95% confidence interval. Pyroptosis by DRAQ7 curve between 0 and 5 h, and apoptosis by pSIVA curve between 5 and 14 h. ns., nonsignificant.

(E) N/TERT-1 cells deleted for DDB2 (ΔDDB2) were treated with ZAK inhibitor (i, 1 μM) and UVB irradiated (100 J/m^2—8 h recovery). Lysates were analyzed as in (A).

(F) As in (E), except that cells were deleted for ERCC2 (ΔERCC2).

(G–I) Cells from (E) and (F) were grown in the presence of the live-cell-staining dye DRAQ7, treated with indicated inhibitors (i, 1 μM), irradiated with UVB (100 J/m^2), and subjected to fluorescence time-lapse microscopy. Statistical significance for pyroptosis was calculated by two-tailed Kolmogorov-Smirnov test between 0 and 5 h. ns., non-significant; ***p < 0.001; ****p ≤ 0.0001.

(J) Stable rescue of U2OS/ΔZAK cells with WT and mutated forms of ZAKα (protein domains and mutations are annotated in Figure S7F). Cells were irradiated with UVB (500 J/m^2—1 h) and lysates were analyzed as in (A).

(K) WT, ΔZAK, and ΔSLFN11 HAP1 cells were irradiated with UVB (500 J/m^2) and analyzed as in (G)–(I). Data are plotted as mean and all error bars represent the standard error of the mean (SEM). **p ≤ 0.01; ***p < 0.001; in two-way ANOVA at the 14-h time point.

(D, G–I, and K) Error bars are from 3 technical replicates that are representative of 1–3 independent repeats. See also Figure S7.

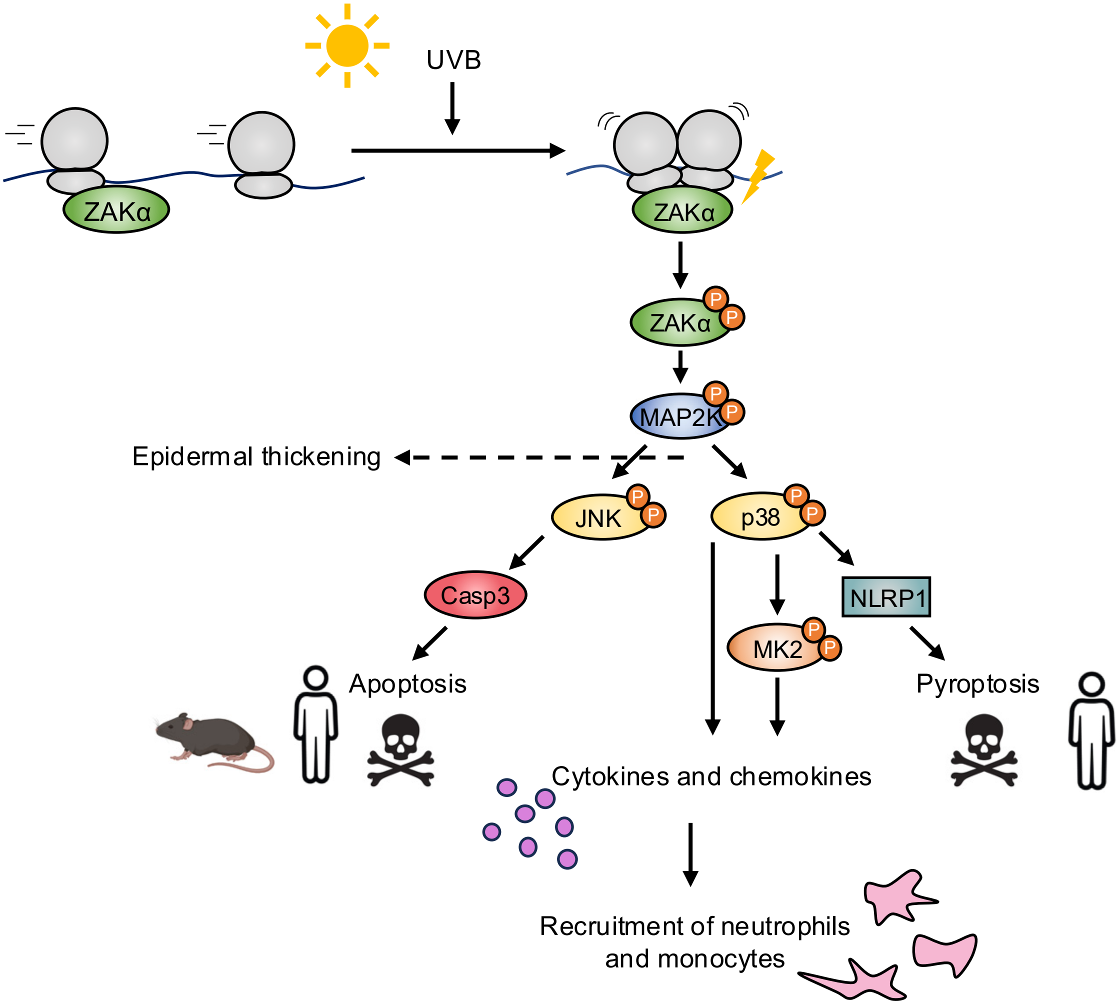

Figure 7. The ribotoxic stress response orchestrates early skin responses to UVB irradiation
In skin keratinocytes, UVB-induced mRNA damage causes ribosome stalling and ensuing collision, providing an activation platform for ZAKα. Downstream, ribotoxic stress response (RSR) signaling mediates rapid p38-dependent pyroptotic cell death in human keratinocytes (right arm) and slower, JNK-dependent apoptotic cell death in mouse and human keratinocytes (left arm). RSR signaling also mediates p38 and MK2-dependent secretion of inflammatory cytokines and chemokines. These events underlie UVB-induced skin infiltration of immune cells and thickening of the epidermal cell layer.

These reactions include skin inflammation, epidermal thickening, and programmed cell death. Our findings also indicate that there are different, or at least temporally and phenotypically distinct, roles for the RSR and DNA damage signaling in sun-exposed skin, which likely has implications for skin immunity and skin carcinogenesis.

Limitations of the study

The insensitivity of the murine inflammasome sensor NLRP1 to regulation by p38[17] did not allow us to capture the full effects of UVB-induced RSR signaling in human skin. The contribution of SLFN11-associated tRNA cleavage to UVB-induced ZAKα activation and cell death is small in HAP1 cells (Figure 6K). Although these cells appear to express higher SLFN11 levels than primary keratinocytes, this effect may be negligible in skin keratinocytes.

RESOURCE AVAILABILITY

Lead contact

Further information and requests for resources and reagents should be directed to and will be fulfilled by the lead contact, Simon Bekker-Jensen (sbj@sund.ku.dk).

Materials availability

Plasmids, cell lines, and other materials generated in this study are available upon reasonable request to the lead contacts.

Data and code availability

- RNA sequencing raw data files have been deposited in NCBI's Gene Expression Omnibus (GEO) archive, with the accession code GEO: GSE251957.
- This paper does not report original code.
- Any additional information required to reanalyze the data reported in this paper is available from the lead contact upon request.

ACKNOWLEDGMENTS

We thank Drs. Claus Johansen (Aarhus University Hospital, Denmark) for supplying primary human keratinocytes, Thijn Brummelkamp (Netherlands Cancer Institute, the Netherlands) for the kind gift of SLFN11 KO HAP1 cells, James Rheinwald (Harvard Medical School, USA) for N/TERT-1 cells, Katrine Baumann (RefLab) for human skin samples, Xiaoyun Lu (Jinan University, China) for the kind gift of ZAK inhibitors, Lone Christiansen (Odense University Hospital, Denmark) for laboratory assistance, and Ryan Lim Yi Da (Skin Research Institute of Singapore, A*STAR, Singapore) for helping us establish and optimize the IMQ mouse model of psoriasis-like dermatitis. Work in the Bekker-Jensen lab was supported by the LEO Foundation (LF-OC-23-001458) and the European Research Council (ERC) under the European Union's Horizon 2020 research and innovation program (grant agreement 863911—PHYRIST). Center for Gene Expression (CGEN) is a Center of Excellence funded by The National Danish Research Foundation (grant no. DNRF166). Work in the Zhong lab is supported by the National Research Foundation (NRF-NRFF11-2019-0006); the Ministry of Education (MOE-T2EP30222-0008); and the National Medical Research Council (MOH-001499), Singapore. Work in the Gyrd-Hansen lab was supported by the LEO Foundation and the Novo Nordisk Foundation (NNF200C0059392). L.F.K. and J.E.A.C. are supported by funding from the Agency for Science, Technology and Research and Agency for Science, Technology and Research BMRC EDB IAF-PP grants—H17/01/a0/004 Skin Research Institute of Singapore, BMRC Central Research Funds (ATR), and NMRC OF-LCG grant (ADEPT: atopic dermatitis research program for patients, OFLCG23-may-0040).

AUTHOR CONTRIBUTIONS

A.C.V., G.S., F.L.Z., and S.B.-J. conceived and designed the study. A.C.V., Z.W., M.J.F., G.S., T.G.A., M.J., J.F.M., T.L.A., M.B., L.F.K., and N.L.M. performed experiments and analyzed data. P.H., J.E.A.C., and M.G.-H. supervised experiments. F.L.Z. and S.B.-J. wrote the manuscript. All authors commented on and edited the manuscript.

DECLARATION OF INTERESTS

The authors declare no competing interests.

STAR★METHODS

Detailed methods are provided in the online version of this paper and include the following:

- KEY RESOURCES TABLE
- EXPERIMENTAL MODEL AND STUDY PARTICIPANT DETAILS
  - Cell lines and primary cells
  - Mice
- METHOD DETAILS
  - Cell culture and reagents
  - Animal experiments
  - Histology
  - Immunohistochemistry
  - *In situ* hybridization
  - Flow cytometry
  - SDS-PAGE and immunoblotting
  - RT-qPCR
  - Live cell imaging and cell death quantification
  - RNAseq sample preparation and analysis
- QUANTIFICATION AND STATISTICAL ANALYSIS

SUPPLEMENTAL INFORMATION

Supplemental information can be found online at https://doi.org/10.1016/j.molcel.2024.10.044.

Received: November 27, 2023
Revised: July 3, 2024
Accepted: October 30, 2024
Published: November 25, 2024

REFERENCES

1. Shain, A.H., and Bastian, B.C. (2016). From melanocytes to melanomas. Nat. Rev. Cancer *16*, 345–358. https://doi.org/10.1038/nrc.2016.37.
2. Wagner, E.F., and Nebreda, A.R. (2009). Signal integration by JNK and p38 MAPK pathways in cancer development. Nat. Rev. Cancer *9*, 537–549. https://doi.org/10.1038/nrc2694.
3. Davis, R.J. (2000). Signal transduction by the JNK group of MAP kinases. Cell *103*, 239–252. https://doi.org/10.1016/s0092-8674(00)00116-1.
4. Gallo, K.A., and Johnson, G.L. (2002). Mixed-lineage kinase control of JNK and p38 MAPK pathways. Nat. Rev. Mol. Cell Biol. *3*, 663–672. https://doi.org/10.1038/nrm906.
5. Ronkina, N., and Gaestel, M. (2022). MAPK-Activated Protein Kinases: Servant or Partner? Annu. Rev. Biochem. *91*, 505–540. https://doi.org/10.1146/annurev-biochem-081720-114505.
6. Vind, A.C., Snieckute, G., Blasius, M., Tiedje, C., Krogh, N., Bekker-Jensen, D.B., Andersen, K.L., Nordgaard, C., Tollenaere, M.A.X., Lund, A.H., et al. (2020). ZAKalpha Recognizes Stalled Ribosomes through Partially Redundant Sensor Domains. Mol. Cell *78*, 700–713.e7. https://doi.org/10.1016/j.molcel.2020.03.021.
7. Vind, A.C., Snieckute, G., Bekker-Jensen, S., and Blasius, M. (2023). Run, Ribosome, Run: From Compromised Translation to Human Health. Antioxid. Redox Signal. *39*, 336–350. https://doi.org/10.1089/ars.2022.0157.
8. Ouyang, D.Y., Wang, Y.Y., and Zheng, Y.T. (2005). Activation of c-Jun N-terminal kinases by ribotoxic stresses. Cell. Mol. Immunol. *2*, 419–425.
9. Wang, X., Mader, M.M., Toth, J.E., Yu, X., Jin, N., Campbell, R.M., Smallwood, J.K., Christe, M.E., Chatterjee, A., Goodson, T., Jr., et al. (2005). Complete inhibition of anisomycin and UV radiation but not cytokine induced JNK and p38 activation by an aryl-substituted dihydropyrrolopyrazole quinoline and mixed lineage kinase 7 small interfering RNA. J. Biol. Chem. *280*, 19298–19305. https://doi.org/10.1074/jbc.M413059200.
10. Wu, C.C.C., Peterson, A., Zinshteyn, B., Regot, S., and Green, R. (2020). Ribosome Collisions Trigger General Stress Responses to Regulate Cell Fate. Cell *182*, 404–416.e14. https://doi.org/10.1016/j.cell.2020.06.006.
11. Snieckute, G., Genzor, A.V., Vind, A.C., Ryder, L., Stoneley, M., Chamois, S., Dreos, R., Nordgaard, C., Sass, F., Blasius, M., et al. (2022). Ribosome stalling is a signal for metabolic regulation by the ribotoxic stress response. Cell Metab. *34*, 2036–2046.e8. https://doi.org/10.1016/j.cmet.2022.10.011.
12. Ryder, L., Arendrup, F.S., Martínez, J.F., Snieckute, G., Pecorari, C., Shah, R.A., Lund, A.H., Blasius, M., and Bekker-Jensen, S. (2023). Nitric oxide-induced ribosome collision activates ribosomal surveillance mechanisms. Cell Death Dis. *14*, 467. https://doi.org/10.1038/s41419-023-05997-5.
13. Wurtmann, E.J., and Wolin, S.L. (2009). RNA under attack: cellular handling of RNA damage. Crit. Rev. Biochem. Mol. Biol. *44*, 34–49. https://doi.org/10.1080/10409230802594043.
14. Stoneley, M., Harvey, R.F., Mulroney, T.E., Mordue, R., Jukes-Jones, R., Cain, K., Lilley, K.S., Sawarkar, R., and Willis, A.E. (2022). Unresolved stalled ribosome complexes restrict cell-cycle progression after genotoxic stress. Mol. Cell *82*, 1557–1572.e7. https://doi.org/10.1016/j.molcel.2022.01.019.
15. Iordanov, M.S., Pribnow, D., Magun, J.L., Dinh, T.H., Pearson, J.A., and Magun, B.E. (1998). Ultraviolet radiation triggers the ribotoxic stress response in mammalian cells. J. Biol. Chem. *273*, 15794–15803. https://doi.org/10.1074/jbc.273.25.15794.
16. Sinha, N.K., McKenney, C., Yeow, Z.Y., Li, J.J., Nam, K.H., Yaron-Barir, T.M., Johnson, J.L., Huntsman, E.M., Cantley, L.C., Ordureau, A., et al. (2024). The ribotoxic stress response drives UV-mediated cell death. Cell *187*, 3652–3670.e40. https://doi.org/10.1016/j.cell.2024.05.018.

CellPress
OPEN ACCESS

17. Robinson, K.S., Toh, G.A., Rozario, P., Chua, R., Bauernfried, S., Sun, Z., Firdaus, M.J., Bayat, S., Nadkarni, R., Poh, Z.S., et al. (2022). ZAKalpha-driven ribotoxic stress response activates the human NLRP1 inflammasome. Science *377*, 328–335. https://doi.org/10.1126/science.abl6324.

18. Jenster, L.M., Lange, K.E., Normann, S., vom Hemdt, A., Wuerth, J.D., Schiffelers, L.D.J., Tesfamariam, Y.M., Gohr, F.N., Klein, L., Kaltheuner, I.H., et al. (2023). P38 kinases mediate NLRP1 inflammasome activation after ribotoxic stress response and virus infection. J. Exp. Med. *220*, e20220837. https://doi.org/10.1084/jem.20220837.

19. Robinson, K.S., Toh, G.A., Firdaus, M.J., Tham, K.C., Rozario, P., Lim, C.K., Toh, Y.X., Lau, Z.H., Binder, S.C., Mayer, J., et al. (2023). Diphtheria toxin activates ribotoxic stress and NLRP1 inflammasome-driven pyroptosis. J. Exp. Med. *220*, e20230105. https://doi.org/10.1084/jem.20230105.

20. Gallagher, R.P., and Lee, T.K. (2006). Adverse effects of ultraviolet radiation: a brief review. Prog. Biophys. Mol. Biol. *92*, 119–131. https://doi.org/10.1016/j.pbiomolbio.2006.02.011.

21. El-Abaseri, T.B., Putta, S., and Hansen, L.A. (2006). Ultraviolet irradiation induces keratinocyte proliferation and epidermal hyperplasia through the activation of the epidermal growth factor receptor. Carcinogenesis *27*, 225–231. https://doi.org/10.1093/carcin/bgi220.

22. Lee, J.W., Ratnakumar, K., Hung, K.F., Rokunohe, D., and Kawasumi, M. (2020). Deciphering UV-induced DNA Damage Responses to Prevent and Treat Skin Cancer. Photochem. Photobiol. *96*, 478–499. https://doi.org/10.1111/php.13245.

23. Prasad, R., and Katiyar, S.K. (2017). Crosstalk Among UV-Induced Inflammatory Mediators, DNA Damage and Epigenetic Regulators Facilitates Suppression of the Immune System. Photochem. Photobiol. *93*, 930–936. https://doi.org/10.1111/php.12687.

24. Gentile, M., Latonen, L., and Laiho, M. (2003). Cell cycle arrest and apoptosis provoked by UV radiation-induced DNA damage are transcriptionally highly divergent responses. Nucleic Acids Res. *31*, 4779–4790. https://doi.org/10.1093/nar/gkg675.

25. Tang, Z., Tong, X., Huang, J., Liu, L., Wang, D., and Yang, S. (2021). Research progress of keratinocyte-programmed cell death in UV-induced Skin photodamage. Photodermatol. Photoimmunol. Photomed. *37*, 442–448. https://doi.org/10.1111/phpp.12679.

26. Lassen, N.E., Andersen, T.L., Pløen, G.G., Søe, K., Hauge, E.M., Harving, S., Eschen, G.E.T., and Delaisse, J.M. (2017). Coupling of Bone Resorption and Formation in Real Time: New Knowledge Gained From Human Haversian BMUs. J. Bone Miner. Res. *32*, 1395–1405. https://doi.org/10.1002/jbmr.3091.

27. Nordgaard, C., Vind, A.C., Stonadge, A., Kjøbsted, R., Snieckute, G., Antas, P., Blasius, M., Reinert, M.S., Del Val, A.M., Bekker-Jensen, D.B., et al. (2022). ZAKβ is activated by cellular compression and mediates contraction-induced MAP kinase signaling in skeletal muscle. EMBO J. *41*, e111650. https://doi.org/10.15252/embj.2022111650.

28. Pasparakis, M., Haase, I., and Nestle, F.O. (2014). Mechanisms regulating skin immunity and inflammation. Nat. Rev. Immunol. *14*, 289–301. https://doi.org/10.1038/nri3646.

29. Skopelja-Gardner, S., An, J., Tai, J., Tanaka, L., Sun, X., Hermanson, P., Baum, R., Kawasumi, M., Green, R., Gale, M., Jr., et al. (2020). The early local and systemic Type I interferon responses to ultraviolet B light exposure are cGAS dependent. Sci. Rep. *10*, 7908. https://doi.org/10.1038/s41598-020-64865-w.

30. Zhong, F.L., Mamaï, O., Sborgi, L., Boussofara, L., Hopkins, R., Robinson, K., Szeverényi, I., Takeichi, T., Balaji, R., Lau, A., et al. (2016). Germline NLRP1 Mutations Cause Skin Inflammatory and Cancer Susceptibility Syndromes via Inflammasome Activation. Cell *167*, 187–202.e17. https://doi.org/10.1016/j.cell.2016.09.001.

31. Gangwar, R.S., Gudjonsson, J.E., and Ward, N.L. (2022). Mouse Models of Psoriasis: A Comprehensive Review. J. Invest. Dermatol. *142*, 884–897. https://doi.org/10.1016/j.jid.2021.06.019.

32. Sakurai, K., Dainichi, T., Garcet, S., Tsuchiya, S., Yamamoto, Y., Kitoh, A., Honda, T., Nomura, T., Egawa, G., Otsuka, A., et al. (2019). Cutaneous p38 mitogen-activated protein kinase activation triggers psoriatic dermatitis. J. Allergy Clin. Immunol. *144*, 1036–1049. https://doi.org/10.1016/j.jaci.2019.06.019.

33. Sakamoto, K., and Nagao, K. (2023). Mouse Models for Atopic Dermatitis. Curr. Protoc. *3*, e709. https://doi.org/10.1002/cpz1.709.

34. Kim, Y.E., Chen, J., Chan, J.R., and Langen, R. (2010). Engineering a polarity-sensitive biosensor for time-lapse imaging of apoptotic processes and degeneration. Nat. Methods *7*, 67–73. https://doi.org/10.1038/nmeth.1405.

35. Zhu, W., Brauchle, M.A., Di Padova, F., Gram, H., New, L., Ono, K., Downey, J.S., and Han, J. (2001). Gene suppression by tristetraprolin and release by the p38 pathway. Am. J. Physiol. Lung Cell. Mol. Physiol. *281*, L499–L508. https://doi.org/10.1152/ajplung.2001.281.2.L499.

36. Marteijn, J.A., Lans, H., Vermeulen, W., and Hoeijmakers, J.H.J. (2014). Understanding nucleotide excision repair and its roles in cancer and ageing. Nat. Rev. Mol. Cell Biol. *15*, 465–481. https://doi.org/10.1038/nrm3822.

37. Vind, A.C., Genzor, A.V., and Bekker-Jensen, S. (2020). Ribosomal stress-surveillance: three pathways is a magic number. Nucleic Acids Res. *48*, 10648–10661. https://doi.org/10.1093/nar/gkaa757.

38. Johansen, V.B.I., Snieckute, G., Vind, A.C., Blasius, M., and Bekker-Jensen, S. (2023). Computational and Functional Analysis of Structural Features in the ZAKalpha Kinase. Cells *12*, 969. https://doi.org/10.3390/cells12060969.

39. Boon, N.J., Oliveira, R.A., Körner, P.R., Kochavi, A., Mertens, S., Malka, Y., Voogd, R., van der Horst, S.E.M., Huismans, M.A., Smabers, L.P., et al. (2024). DNA damage induces p53-independent apoptosis through ribosome stalling. Science *384*, 785–792. https://doi.org/10.1126/science.adh7950.

40. Zhang, P., Hu, X., Li, Z., Liu, Q., Liu, L., Jin, Y., Liu, S., Zhao, X., Wang, J., Hao, D., et al. (2024). Schlafen 11 triggers innate immune responses through its ribonuclease activity upon detection of single-stranded DNA. Sci. Immunol. *9*, eadj5465. https://doi.org/10.1126/sciimmunol.adj5465.

41. Westman, J., Grinstein, S., and Marques, P.E. (2019). Phagocytosis of Necrotic Debris at Sites of Injury and Inflammation. Front. Immunol. *10*, 3030. https://doi.org/10.3389/fimmu.2019.03030.

42. Singh, R., Letai, A., and Sarosiek, K. (2019). Regulation of apoptosis in health and disease: the balancing act of BCL-2 family proteins. Nat. Rev. Mol. Cell Biol. *20*, 175–193. https://doi.org/10.1038/s41580-018-0089-8.

43. Wang, Y., Gao, W., Shi, X., Ding, J., Liu, W., He, H., Wang, K., and Shao, F. (2017). Chemotherapy drugs induce pyroptosis through caspase-3 cleavage of a gasdermin. Nature *547*, 99–103. https://doi.org/10.1038/nature22393.

44. Yamanaka, K., Yamamoto, O., and Honda, T. (2021). Pathophysiology of psoriasis: A review. J. Dermatol. *48*, 722–731. https://doi.org/10.1111/1346-8138.15913.

45. Langan, S.M., Irvine, A.D., and Weidinger, S. (2020). Atopic dermatitis. Lancet *396*, 345–360. https://doi.org/10.1016/S0140-6736(20)31286-1.

46. Brooks, D., Burke, E., Lee, S., Eble, T.N., O'Leary, M., Osei-Owusu, I., Rehm, H.L., Dhar, S.U., Emrick, L., Bick, D., et al. (2024). Heterozygous MAP3K20 variants cause ectodermal dysplasia, craniosynostosis, sensorineural hearing loss, and limb anomalies. Hum. Genet. *143*, 279–291. https://doi.org/10.1007/s00439-024-02657-2.

47. Yang, J., Shibu, M.A., Kong, L., Luo, J., BadrealamKhan, F., Huang, Y., Tu, Z.C., Yun, C.H., Huang, C.Y., Ding, K., et al. (2020). Design, Synthesis, and Structure-Activity Relationships of 1,2,3-Triazole Benzenesulfonamides as New Selective Leucine-Zipper and Sterile-alpha Motif Kinase (ZAK) Inhibitors. J. Med. Chem. *63*, 2114–2130. https://doi.org/10.1021/acs.jmedchem.9b00664.

48. LaFontaine, E., Miller, C.M., Permaul, N., Martin, E.T., and Fuchs, G. (2020). Ribosomal protein RACK1 enhances translation of poliovirus and other viral IRESs. Virology *545*, 53–62. https://doi.org/10.1016/j.virol.2020.03.004.

STAR★METHODS

KEY RESOURCES TABLE

REAGENT or RESOURCE	SOURCE	IDENTIFIER
Antibodies		
Rabbit polyclonal anti-ZAKα	Bethyl	Cat#A301-993A; RRID: AB_1576612
Mouse monoclonal anti-phospho-p38	Cell Signaling	Cat#9216; RRID: AB_331296
Rabbit monoclonal anti-phospho-p38	Cell Signaling	Cat#4511; RRID: AB_2139682
Mouse polyclonal antibody anti-p38	Cell Signaling	Cat#9212; RRID: AB_330713
Mouse monoclonal anti-phospho-SAPK/JNK	Cell Signaling	Cat#9255; RRID: AB_2307321
Rabbit monoclonal anti-phospho-SAPK/JNK	Cell Signaling	Cat#4668; RRID:AB_823588
Rabbit monoclonal anti-SAPK/JNK	Cell Signaling	Cat#9258; RRID: AB_2141027
Rabbit polyclonal anti-ZAK	Proteintech	Cat#14945-1-AP; RRID: AB_1064269
Mouse monoclonal anti-p150	BD biosciences	Cat#610473; RRID: AB_397845
Mouse monoclonal anti-α-Tubulin	Merck	Cat#T9026; RRID: AB_477593
Mouse monoclonal anti-HA-tag	Santa Cruz Biotechnology	Cat#sc-7392 HRP; RRID: AB_2894930
Rabbit monoclonal anti-phospho-GCN2	Abcam	Cat#ab75837; RRID: AB_1310587
Rabbit monoclonal anti-phospho-eIF2alpha	Cell Signaling	Cat#3398; RRID: AB_2096481
Rabbit polyclonal anti-cleaved Caspase 3	Cell Signaling	Cat#9661; RRID:AB_2341188
Rabbit polyclonal anti-Caspase 3	Santa Cruz	Cat#sc-7148; RRID:AB_637828
Rabbit monoclonal anti-phospho-Chk2	Cell Signaling	Cat#2197; RRID:AB_2080501
Rabbit monoclonal anti-phospho-Chk1	Cell Signaling	Cat#2348; RRID:AB_331212
Mouse monoclonal anti-γ-tubulin	Merck	Cat#T5326; RRID:AB_532292
Rabbit monoclonal anti-MK2	Cell Signaling	Cat#3042; RRID: AB_2141314
Rabbit polyclonal anti-GSDMDC1	Novus Bio	Cat#NBP2-33422; RRID:AB_2687913
Rabbit polyclonal anti-ERCC2	Proteintech	Cat#10818-1-AP; RRID:AB_2231330
Rabbit monoclonal anti -IL1β p17 specific	Cell Signaling	Cat#83186; RRID:AB_2800010
Rabbit monoclonal anti-Myeloperoxidase	Abcam	#ab208670; RRID:AB_2864724
Mouse monoclonal anti-GAPDH	Proteintech	60004-1-Ig; RRID:AB_2107436
Rabbit polyclonal anti-DDB2	Abcam	#ab181136; RRID:AB_2889873
Rabbit polyclonal anti-Cleaved PARP1 (Asp 214)	Cell Signaling	#9541; RRID:AB_331426
Alexa Fluor 700 Mouse anti-NK1.1, Clone PK136	BD	#560515; RRID:AB_10612564
Alexa Fluor 700 Rat anti-CD19, Clone 1D3	BD	#557958; RRID:AB_396958
Alexa Fluor 700 Rat anti-CD45R (B220) clone RA3-6B2	Invitrogen	#56-0452-82;RRID:AB_891458
Alexa Fluor 700 Hamster anti-TCRb clone H57-597	Invitrogen	#56-5961-82; RRID:AB_2802349
Alexa Fluor700 Rat anti-CD3 clone 17A2	BioLegend	#100216; RRID:AB_493697
PE Mouse anti-CD64 clone X54-5/7.1	Invitrogen	#12-0641-82; RRID:AB_2735014
BV711 Rat anti-Siglec-F clone E50-2440	BD	#740764; RRID:AB_2740427
BV421 Rat anti-CD172a clone P84	BD	#740071; RRID:AB_2739835
BV650 Mouse anti-XCR1 clone ZET	BioLegend	#148220; RRID:AB_2566410
PE-CF594 Rat anti-F4/80 clone T45-2342	BD	#565613; RRID:AB_2734770
APC Rat anti-Ly-6C clone AL-21	BD	#560595; RRID:AB_1727554
BUV395 Rat anti-CD45 clone 30-F11	BD	#564279; RRID:AB_2651134
BB700 Rat anti-CD192 (CCR2) clone 475301	BD	#747965; RRID:AB_2872426
BV605 Rat anti-CD86 clone GL1	BD	#563055; RRID:AB_2737977
BV785 Rat anti-CD326 (Ep-CAM) clone G8.8	BioLegend	#118245; RRID:AB_2860639
Alexa Fluor 488 Rat anti-MCHII I-A/I-E clone M5/114.15.2	BioLegend	#107616; RRID:AB_493523

(Continued on next page)

CellPress
OPEN ACCESS

Continued

REAGENT or RESOURCE	SOURCE	IDENTIFIER
BV480 Rat anti-CD24 clone M1/69	BD	#746709; RRID:AB_2743976
APC-eFluor 780 Rat anti-CD11b clone M1/70	Invitrogen	#47-0112-82; RRID:AB_1603193
BUV737 Rat anti-Ly-6G clone 1A8	BD	#741813; RRID:AB_2871151
PE-Cyanine7 Hamster anti-CD11c clone N418	Invitrogen	#25-0114-81; RRID:AB_469589
BV605 Rat anti-CD4 clone GK1.5	BD	#743156; RRID:AB_2741309
BUV737 Rat anti-CD8a clone 53-6.7	BD	#612759; RRID:AB_2870090
APC-Cy™7 Mouse anti-NK-1.1 clone PK136	BD	#560618; RRID:AB_1727569
V500 Rat anti-CD44 clone IM7	BD	#560781; RRID:AB_1937328
BV786 Hamster anti-CD69 clone H1.2F3	BD	#564683; RRID:AB_2738890
PE Hamster anti-CD3e clone 145-2C11	Invitrogen	#12-0031-81; RRID:AB_465495
APC Rat anti-CD62L clone MEL-14	BD	#561919; RRID:AB_10895379
BB700 Hamster anti-CD103 clone 2E7	BD	#748240; RRID:AB_2872671
FITC Hamster anti-Vγ 3 TCR clone 536	BD	#553229; RRID:AB_394721
BV421 Hamster anti- γδ T-Cell Receptor clone GL3	BD	#562892; RRID:AB_2737871
PE-Cy7 Hamster anti-TCR β Chain clone H57-597	BD	#560729; RRID:AB_1937310
Biological samples		
Human skin sample	A gift from Katrine Baumann, RefLab	N/A
Chemicals, peptides, and recombinant proteins		
Doxycycline	Merck	D3347
Anisomycin	Merck	A9789
Puromycin	Cayman Chemical	13884
ZAK inhibitor 6p	Yang et al.[47]	N/A
p38 inhibitor (BIRB 796)	Tocris Bioscience	Cat#5989
JNK inhibitor (JNK-IN-8)	Merck	Cat#SML1246
MK2 inhibitor	Merck	# PF3644022
p38 inhibitor (Neflamapimod)	MedChemExpress	HY-10328
JNK inhibitor (Bentamapimod)	MedChemExpress	HY-14761
ATR inhibitor (Ceralasertib)	MedChemExpress	HY-19323
Q5 Hot Start High-Fidelity DNA Polymerases	NEB	M0493S
Q5 Reaction Buffer (5x)	NEB	B9027S
Imiquimod (IMQ)	Aldara®, Glenmark Pharmaceuticals Ltd	N/A
MC903	Cayman Chemical	10009599
DRAQ7	Abcam	ab109202
DRAQ7	Invitrogen	# D15105
DAPI	Merck	MBD0015-1ML
PI	Abcam	ab14083
Nucleofector P3+ Supplement	Lonza	#V4XP-3032#
Alt-R S.p. HiFi Cas9 nuclease V3	Integrated DNA technologies	N/A
Critical commercial assays		
RevertAid RT Reverse Transcription Kit	Thermo Fisher Scientific	Cat#K1691
5x HOT FIREPol EvaGreen qPCR Mix Plus (NO ROX)	Bioline	08-25-00001-5
Kinetic Apoptosis Kit (pSIVA)	Abcam	ab129817
Epredia UltraVision Quanto Detection System HRP Kit	Thermo Fisher Scientific	TL-060-QHD
3′-Diaminobenzidine	Thermo Fisher Scientific	TA-060-QHDX

(*Continued on next page*)

Continued		
REAGENT or RESOURCE	SOURCE	IDENTIFIER
RNAeasy Mini Kit	Qiagen	#74004
ApopTag Plus Peroxidase *In Situ* Apoptosis Kit	Merck	#S7101
Permanent Red Substrate-Chromogen, Liquid	Agilent	#KO64030-2
Deposited data		
RNAseq	GEO: GSE251957	N/A
Experimental models: Cell lines		
Human osteosarcoma cells (U2OS)	ATCC	HTB-96; RRID: CVCL0042
U2OS ΔZAK	Vind et al.[6]	N/A
U2OS ΔZAK + Strep-HA-ZAKα	Vind et al.[6]	N/A
U2OS ΔZAK + Strep-HA- ZAKα ΔCTD	Vind et al.[6]	N/A
U2OS ΔZAK + Strep-HA- ZAKα_R/K→A	Johansen et al.[38]	N/A
U2OS ΔZAK + Strep-HA- ZAKα_R/K→A, ΔCTD	Johansen et al.[38]	N/A
U2OS ΔZAK + Strep-HA-ZAKα_K45A	Vind et al.[6]	N/A
U2OS ΔZAK + Strep-HA-ZAKα_S657A	Vind et al.[6]	N/A
HAP1	Boon et al.[39]	N/A
HAP1 ΔZAK	Boon et al.[39]	N/A
HAP1 ΔSLFN11	Boon et al.[39]	N/A
N/TERT	Gift from James Rheinwald, (Harvard Medical School, USA)	N/A
N/TERT ZAK KO	Robinson et al.[17]	N/A
N/TERT DDB2 KO #2	https://www.biorxiv.org/content/10.1101/2022.01.24.477516v1	N/A
N/TERT DDB2 KO #3	https://www.biorxiv.org/content/10.1101/2022.01.24.477516v1	N/A
N/TERT ERCC2 KO #1	This paper	N/A
N/TERT ERCC2 KO #2	This paper	N/A
N/TERT ERCC2 KO #3	This paper	N/A
Mouse primary keratinocytes	N/A	N/A
Human primary keratinocytes	Gift from Claus Johansen (Aarhus University Hospital, Denmark)	N/A
Experimental models: Organisms/strains		
ZAK knockout mice in the C57BL/6 background	Nordgaard et al.[27]	N/A
ZAK knockout mice in C57BL/6NJ	This paper	N/A
Oligonucleotides		
DDB2 sg2: 5'-TGTAGCCCTCCTGTCAAAGG-3'	https://www.biorxiv.org/content/10.1101/2022.01.24.477516v1	N/A
DDB2 sg3: 5'-CCCAACTCACCCCAGCACCG-3'	https://www.biorxiv.org/content/10.1101/2022.01.24.477516v1	N/A
ERCC2 B: 5'- AGTCGTACGGGAAGTAGACC-3'	This paper	N/A
ERCC2 A: 5'-GGCCTCGTCGAAGACCACGA-3'	This paper	N/A
ERCC2 C: 5'- GCTTTGGGAAGGACGTCGAT'-'3'	This paper	N/A
15 probe pairs targeting nucleotides 1957-2637 of mouse *Map3k20* transcriptional variant 1 mRNA	Advanced Cell Diagnostics	#1095491-C1
13 probe-pairs targeting nucleotides 1178-1900 of mouse *Map3k20* transcriptional variant 3 mRNA	Advanced Cell Diagnostics	#1095481-C1
14 probe-pairs targeting nucleotides 1895-2579 of human MAP3K20 transcriptional variant 1 mRNA	Advanced Cell Diagnostics	#1095471-C1
10 probe-pairs targeting nucleotide 1180-1828 of human MAP3K20 transcriptional variant 2 mRNA	Advanced Cell Diagnostics	#1095461-C1

(*Continued on next page*)

CellPress
OPEN ACCESS

Continued

REAGENT or RESOURCE	SOURCE	IDENTIFIER
qPCR primers can be found in Table S2	–	N/A
Recombinant DNA		
LentiCRISPR-V2	Addgene	#52961
Software and algorithms		
Prism	GraphPad Software	https://www.graphpad.com/
QuPath	–	https://qupath.github.io/
FlowJo	FlowJo	https://www.flowjo.com
[skimage]	–	https://jmlr.csail.mit.edu/papers/v12/pedregosa11a.html
[Cellpose3]	–	https://www.cellpose.org
Fuji (Image J)	Fuji (Image J)	https://imagej.net/software/fiji/
Other		
EpiLife CF Kit	Thermo Fisher Scientific	#MEPICF500
EpiLife Defined Growth Supplement	Thermo Fisher Scientific,	#S0125
Keratinocyte Serum-Free Medium (KSF-M)	Gibco	17005042
Bovine pituitary extract	Gibco	13028-014
Human recombinant EGF	Gibco	10450-013
Keratinocyte-SFM Medium (Kit) with L-glutamine, EGF, and BPE	Gibco	#17005075
Dispase digestion buffer	Thermo Fisher Scientific	#17105041
StemPro™ Accutase™ Cell Dissociation Reagent	Thermo Fisher Scientific	A1110501
CountBright™ Absolute Counting Beads	Thermo Fisher Scientific	#C36950
FBS, CERT, USA origin, 500 ml	Thermo Fisher Scientific	#16000044
Purified Rat Anti-Mouse CD16/CD32 (Mouse BD Fc Block™)	BD	#553142

EXPERIMENTAL MODEL AND STUDY PARTICIPANT DETAILS

Cell lines and primary cells

Female human osteosarcoma cells (U2OS) cells were cultured in Dulbecco's Modified Eagle's Medium (DMEM, Biowest) supplemented with a 10% fetal bovine serum (FBS, Biowest), L-glutamine, penicillin and streptomycin. Male human near haploid cells (HAP1) were cultured in Iscove's Modified Dulbecco's Medium (IMDM) GlutaMAX™ Supplement supplemented with 10% FBS, 1% penicillin, and streptomycin. Female and male human and mouse primary keratinocytes were cultured in EpiLife CF Kit supplemented with EpiLife Defined Growth Supplement and 1% penicillin and streptomycin. Immortalized male human keratinocytes (N/TERT-1) were cultured in Keratinocyte Serum-Free Medium (KSF-M) supplemented with final concentration of 25 μg/ml of bovine pituitary extract, 294.4 pg/ml of human recombinant EGF and 300 μM $CaCl_2$. All cells were cultured at 37°C in a humidified 5-8% CO_2 cell incubator

Mice

For UV experiments, mice were housed at the animal facility of the Department of Experimental Medicine at the University of Copenhagen and the research was monitored by the Institutional Animal Care and Use Committee. All the mouse work was performed in compliance with Danish and European regulations. ZAK knockout mouse in the C57BL/6 background was a gift from Vivian S. W. Li (Crick Institute, United Kingdom), and C57BL/6 WT and ZAK KO mice were obtained by in-house breeding. For anisomycin, IMQ and MC903 experiments, mice were housed at the Animal Research Facility, Lee Kong Chian School of Medicine, NTU and the research was monitored by the Research Integrity Office, NTU Institutional Animal Care and Use Committee. *Zak* knockout mice housed in Singapore were generated by the Knockout Mouse Genotyping Program (KOMP2) at the Jackson Laboratory (MMRRC Strain # 42321-JAX). At both facilities, mice were maintained on a 12-h light:dark cycle and were allowed to eat ad libitum of commercial rodent chow and water prior to the experiment. At the time of the experiment female littermates were randomly assigned to experimental groups when they were 7-10 weeks of age.

METHOD DETAILS

Cell culture and reagents

Human osteosarcoma cells (U2OS) were cultured in Dulbecco's Modified Eagle's Medium (DMEM, Biowest) supplemented with 10% fetal bovine serum (FBS, Biowest), L-glutamine, 1% penicillin and streptomycin. Human near haploid cells (HAP1) were cultured in Iscove's Modified Dulbecco's Medium (IMDM) GlutaMAX Supplement (#31980022, Thermo Fisher Scientific) supplemented with 10% FBS, 1% penicillin and streptomycin. Primary human and mouse keratinocytes were cultured in EpiLife CF Kit (Thermo Fisher Scientific, #MEPICF500) supplemented with EpiLife Defined Growth Supplement (Thermo Fisher Scientific, #S0125) and 1% penicillin and streptomycin. Immortalized human keratinocytes (N/TERT-1) were grown in Keratinocyte Serum-Free Medium (KSF-M) (Gibco, 17005042) supplemented with final concentration of 25 μg/ml of bovine pituitary extract (Gibco, 13028-014), 294.4 pg/ml of human recombinant EGF (Gibco, 10450-013) and 300 μM $CaCl_2$. All cells were cultured at 37 °C in a humidified 5% CO_2 cell incubator. Experiments were conducted when the cells reached 70–80% confluency.

U2OS ΔZAK, HAP1 ΔZAK, HAP1 ΔSLFN11, N/TERT-1 ΔZAK, N/TERT-1 ΔNLRP1 and N/TERT-1 Δp38αβ cells were all previously described.[6,17,27,39,48] Stable cell lines expressing wildtype, truncated or mutated versions of strep-HA-tagged ZAKα were previously described.[38] Lentiviral Cas9 and guide RNA plasmid (LentiCRISPR-V2, Addgene plasmid #52961) was used to create stable deletions in N/TERT-1 keratinocytes using sgRNAs target sequences DDB2 (key resources table). KO efficiency was evaluated by immunoblot. For generating ERCC2 knockout cells, cells were nucleofected with CRISPR/Cas9 RNPs with crRNAs A, B and C (key resources table). ΔERCC2 #1 was nucleofected with crRNA B, ΔERCC2 #2 was nucleofected with cRNA A+C and ΔERCC2 #3 was nucleofected with all three cRNA. Briefly, crRNA and tracrRNA are annealed and assembled into ribonucleoprotein with Cas9 nuclease (Alt-R S.p. HiFi Cas9 nuclease V3). 400,000 cells were washed with PBS and resuspended in Nucleofector P3+ Supplement (Lonza, #V4XP-3032#) before being mixed with Cas9 RNP and electroporated using the Electroporator (Amaxa Nucleofactor). Afterwards cells were plated in a 6 cm dish. KO efficiency was evaluated a week later by immunoblot.

Primary mouse keratinocytes were derived from our WT and $ZAK^{-/-}$ mice. In brief, mouse tail skin was peeled off and rinsed with sterile PBS in a Petri dish. Subsequently, skin pieces were placed in Eppendorf tubes pre-filled with 2 ml of ice-cold dispase digestion buffer (4 mg/ml dispase, (Thermo Fisher Scientific, #17105041) in keratinocyte growth medium (Thermo Fisher Scientific, #MEPICF500)). Tubes were incubated and rotated at 4° C overnight. The following morning, isolated epidermal layers were individually transferred to 6 cm dishes, each containing 3 ml of trypsin-based digestion solution (Thermo Fisher Scientific, # 12605010). Samples were incubated at 37 °C for 20 minutes with continuous agitation. Afterwards, 3 ml of supplemented Keratinocyte Growth Medium (Thermo Fisher Scientific, #MEPICF500) was added to the dishes. The inner surface of the epidermis was then rigorously rubbed using forceps to detach and release the keratinocytes. This procedure was repeated twice, each time with an addition of 3 ml of growth medium. The medium, now containing the released cells, was sequentially passed through 100 μm and 70 μm filters. This was succeeded by centrifugation for 5 minutes at 500 x g. Pellets were then resuspended in supplemented keratinocyte growth medium and seeded at a density of 8×10^4 /cm^2 in dishes pre-coated with a collagen coating solution (Merck, #125-50). 24 hours post initial seeding, medium was replaced to eliminate any unadhered cells. Medium was refreshed every 48 hours until cells reached the targeted confluency in preparation for experiments.

Chemicals and inhibitors used in this study were: Doxycycline (Merck, D3347, 0.13 μg/ml, overnight), anisomycin (Merck, A9789), puromycin (Cayman Chemical, 13884), ZAK inhibitor 6p[47] (a gift from Xiaoyun Lu), p38 inhibitor (BIRB 796, Tocris Bioscience, Cat#5989), JNK inhibitor (JNK-IN-8, Merck, Cat#SML1246), MK2 inhibitor (Merck, # PF3644022), Neflamapimod (MCE, HY-10328), Bentamapimod (MCE, HY-14761) and ATR inhibitor (Ceralasertib, MCE, HY-19323). UVB irradiation was delivered using an Opsytec Dr. Grobel Irradiation Chamber BS-02 and Dosimeter system UV-Mat or the BIO-SUN microprocessor-controlled, cooled UV irradiation system (BIO-SUN, Vilber).

Animal experiments

Mice were housed at the animal facility of the Department of Experimental Medicine at the University of Copenhagen and the research was monitored by the Institutional Animal Care and Use Committee. All the mouse work was performed in compliance with Danish and European regulations. ZAK knockout mice in the mixed C57BL/6JN background were previously described[27] and were maintained by in-house breeding. For UVB experiments, 8-9-weeks-old females or males were shaved, and remaining hair was removed with hair removal cream 48 hours prior to UVB exposure (500 J/m^2). Mice were anesthetized for immobilization during shaving and irradiation using isoflurane. For experiments involving anisomycin, IMQ and MC903, mice were housed at the Animal Research Facility, Lee Kong Chian School of Medicine, NTU and the research was monitored by the Research Integrity Office, NTU Institutional Animal Care and Use Committee. For Anisomycin (Wako Pure Chemicals Industries, Japan), ears of mice aged 8-10 weeks were treated daily with ethanol (vehicle) or 2 mg/mL anisomycin at a total volume of 10 μl of each side of the ear for 5 days. Ear thickness was measured daily with a caliper. For IMQ, shaved dorsal skin on mice aged 7-10 weeks was treated daily with a topical dose of 62.5 mg of commercially available IMQ cream (Aldara®, Glenmark Pharmaceuticals Ltd, USA 5% IMQ) or Vaseline for 5 days. For MC903, ears of mice aged 8-10 weeks were treated daily with ethanol (vehicle) or topical dose of 1 nmol MC903 at a total volume of 10 μl on each side of the ear for 14 days.

Histology

Mouse skin was fixed in 10% formalin solution (Merck, HT501128) for 48 hours at 4 °C by placing it on Whatman paper and submerging it in a petri dish. Fixed tissues were embedded in paraffin and sections were stained by hematoxylin and eosin (H/E) for histology. Images were acquired using ZEISS Axioscan 7 (Zeiss Microscopy), with 20X magnification and a numerical aperture of 0.69. Epidermal thickness was measured by QuPath (https://qupath.github.io/) and ImageJ. For histological scoring of inflammation and cell death, H/E-stained slides were scored in a semiquantitative manner. Inflammation was annotated by scores ranging from 0-3: no visible inflammation (score 0), presence of intraluminal inflammation, mainly neutrophils in hypodermis (score 1), intraluminal inflammatory cells in hypodermis and light dermal interstitial inflammation, mainly by mononuclear cells (score 2), or moderate/brisk interstitial inflammation in both dermis and hypodermis (score 3). Cell death was annotated by scores ranging from 0-2: no visible apoptotic/necrotic cells (score 0), presence of single apoptotic/necrotic cells (score 1), or presence of small clusters (more than 2) of apoptotic cells (score 2). All histological evaluations were performed by a trained pathologist.

Immunohistochemistry

Paraffin-embedded sections were rehydrated in the following steps: 2x Xylene washes (10 min), 3x 99% ethanol (1-2 min), 2x 96% (1 min) and 1x 70% ethanol (3 min). Subsequently, slides were transferred to Easy-Dip staining jars (VWR, # 720-0791) and rinsed in deionized (DI) water. For antigen retrieval, slides were boiled for 3x 6 min in sodium citrate buffer (100 mM of citric acid monohydrate, pH 6.0, Merck, #1002440500) or Tris-EDTA (10 mM Tris base (VWR, #103156x), 1 mM EDTA solution, pH 9.0, 0.05% Tween 20 (Merck, #P1379)). After slides had cooled to room temperature, they were rinsed in DI water, washed thrice in TBS and treated for 10 min with a 3% hydrogen peroxide solution diluted in methanol (Merck, #H1009). Slides were then rinsed with TBS-T (TBS + 0.1% Tween 20) and stained using the Epredia UltraVision Quanto Detection System HRP Kit (Thermo Fisher Scientific, TL-060-QHD). Antibodies were diluted in 2% BSA in TBS and applied onto the slides for overnight incubation in a humidified chamber (Thermo Fisher Scientific, #15518996). Next, slides were washed 3x 5 min in TBS-T before staining with secondary antibody according to manufacturer's protocol. Afterwards, slides were washed twice in TBS and exposed to 3'-Diaminobenzidine (DAB, Thermo Fisher Scientific, TA-060-QHDX) solution for 5 min. Slides were then rinsed in DI water and placed in hematoxylin (Cell Signaling Technology, # CST-14166S) for 5 sec and again rinsed in DI water. The dehydration process was performed in the following steps: 2x 96% ethanol (1-2 min), 2x 99% ethanol (1 min) and 3x Xylene (4 min). Slides were mounted in Pertex (Histolab, # 00840-05).

The following antibodies were used: anti-phospho-SAPK/JNK (Cell Signaling Technology, #4668, dilution 1:50, pH=6 Sodium Citrate), anti-phospho-p38 (Abcam, #4511S, dilution 1:750, pH=6 Sodium Citrate), anti-cleaved Caspase 3 (Cell Signaling, Cat#9661, dilution 1:400, pH=6 Sodium Citrate), anti-Myeloperoxidase (Abcam, #ab208670, dilution 1:1000, pH=9 Tris-EDTA), anti-ZAKα (Bethyl, #A301-994A and A301-993A, dilution 1:500, pH=6 Sodium Citrate).

For TUNEL staining, the same rehydration and antigen retrieval steps (pH=6 Sodium Citrate) were performed, and slides were stained using ApopTag Plus Peroxidase *In Situ* Apoptosis Kit (Merck, #S7101) according to manufacturer's protocol before dehydration and mounting.

In situ hybridization

3.5-μm-thick paraffin sections of mouse and human skin were *in situ* hybridized using a previously established enhanced version of the RNAScope 2.5 HD procedure (Advanced Cell Diagnostics (ACD), #310035).[26] The sections were deparaffinized, rehydrated, blocked for endogenous peroxidase activity and pretreated using heat-treatment and pepsin treatment.[26] Hybridization was performed using 15 probe-pairs targeting nucleotides 1957-2637 of mouse *Map3k20* transcriptional variant 1 mRNA (m*Zak*α) (ACD, #1095491-C1), 13 probe-pairs targeting nucleotides 1178-1900 of mouse *Map3k20* transcriptional variant 3 mRNA (m*Zak*β) (ACD, #1095481-C1), 14 probe-pairs targeting nucleotides 1895-2579 of human MAP3K20 transcriptional variant 1 mRNA (h*Zak*α) (ACD, #1095471-C1), 10 probe-pairs targeting nucleotide 1180-1828 of human MAP3K20 transcriptional variant 2 mRNA (h*Zak*β) (ACD, #1095461-C1). Probes were detected using branching, tyramide signal amplification and visualization using liquid permanent red (Agilent, #KO64030-2) as previously published.[26] Sections were finally stained with hematoxylin and mounted with Aquatex.

Flow cytometry

6 hours after UVB exposure, dorsal skin was collected in R10 medium (RPMI containing 10% FBS, 1% penicillin and streptomycin, 5% HEPES, 0.1% β-Mercaptoethanol (β-ME)). A 2.5 cm x 2.5 cm piece of back skin was minced and digested using 1 mg/ml Collagenase IV (Merck, #C5138) and 0.1 mg/ml DNase I (Merck, #10104159001) in 7 ml R10. Digestion was performed in a 37 °C incubator with continuous stirring at 800 rpm for 1 hour. Afterwards, the digested tissue was mechanically disrupted using a 70 μm filter and collected in R10 medium. The cell suspension was centrifuged at 500 g for 5 min at 4 °C. Supernatant was removed and cells were further processed through a 40 μm filter. The cell suspension was then equally split into two parts for each staining panel and again centrifuged at 500 g for 5 min at 4 °C.

To avoid nonspecific antibody binding, the cell pellet was resuspended in 100 μl Fc block (1:100 CD16/32 antibody in FACS buffer (2 % FBS in 1x PBS)) and incubated for 15 min on ice. The cell suspension was then transferred into a 96-well plate, washed with 100 μl FACS buffer, and centrifuged at 900 g for 3 min at 4 °C. Afterwards, the cells were incubated with fluorescent labeled antibodies for 30 min on ice. An additional washing step was performed and finally the cells were transferred through a 40 μm filter

into FACS tubes and 25 μl counting beads were added. Shortly before acquisition, DAPI (1:1000) was added as a viability dye. Cells were acquired using a LSRFortessa X-20 (BD Biosciences) cytometer. Flow cytometric data analysis was performed using FlowJo 10.9 (FlowJo LLC).

Antibody staining panels

Myeloid cells

Antigen	Conjugate	Clone	Dilution	Supplier	Cat. #
NK1.1	AF700	PK136	1:100	BD	560515
CD19	AF700	1D3	1:100	BD	557958
B220	AF700	RA3-6B2	1:100	Invitrogen	56-0452-82
TCRb	AF700	H57-597	1:100	Invitrogen	56-5961-82
CD3e	AF700	17A2	1:100	BioLegend	100216
CD64	PE	X54-5/7.1	1:100	Invitrogen	12-0641-82
SiglecF	BV711	E50-2440	1:200	BD	740764
CD172a/SIRPa	BV421	P84	1:200	BD	740071
XCR1	BV650	ZET	1:200	BioLegend	148220
F4/80	PE-CF594	T45-2342	1:200	BD	565613
Ly6C	APC	AL-21	1:200	BD	560595
CD45	BUV395	30-F11	1:200	BD	564279
CCR2	BB700	475301	1:200	BD	747965
CD86	BV605	GL1	1:200	BD	563055
CD326 (Ep-CAM)	BV786	G8.8	1:200	BioLegend	118245
MHCII I-A/I-E	AF488	M5/114.15.2	1:400	BioLegend	107616
CD24	BV480	M1/69	1:400	BD	746709
CD11b	APC-eF780	M1/70	1:400	Invitrogen	47-0112-82
Ly6G	BUV737	1A8	1:400	BD	741813
CD11c	PE-Cy7	N418	1:600	Invitrogen	25-0114-81

T cells

Antigen	Conjugate	Clone	Dilution	Manufacturer	Cat. #
CD45	BUV395	30-F11	1:200	BD	564279
CD4	BV605	GK1.5	1:200	BD	743156
CD8	BUV737	53-6.7	1:200	BD	612759
NK1.1	APC-Cy7	PK136	1:200	BD	560518
CD44	V500	IM7	1:200	BD	560781
CD69	BV786	H1.2F3	1:200	BD	564683
CD3e	PE	145-2C11	1:200	Invitrogen	12-0031-81
CD62L	APC	MEL-14	1:200	BD	561919
CD103	BB700	2E7	1:200	BD	748240
Vg5	FITC	536	1:200	BD	553229
TCRgd	BV421	GL3	1:200	BD	562892
TCRb	PE-Cy7	H57-597	1:400	BD	560729

SDS-PAGE and immunoblotting

After indicated treatments, cells were lysed in EBC buffer (50 mM Tris, pH 7.5, 150 mM NaCl, 1 mM EDTA, 0.5% NP-40, protease and phosphatase inhibitors), mixed with Laemmli sample buffer and boiled for 5-10 min. Protein samples were resolved by SDS-PAGE and transferred to nitrocellulose or PVDF membranes. Membranes were blocked in PBS-T + 5% milk before incubation with primary antibody overnight at 4 °C. Next, membranes were washed in PBS-T and incubated with secondary antibody for 1 h at room temperature. Finally, membranes were washed in PBS-T and visualized by chemiluminescence (Clarity Western ECL substrate, Bio-Rad) using the Bio-Rad Chemidoc imaging system.

The following antibodies were used: Rabbit polyclonal anti-ZAKα (Bethyl, Cat#A301-993A; RRID: AB_1576612), Mouse monoclonal anti-phospho-p38 (Cell Signaling, Cat#9216; RRID: AB_331296) Rabbit monoclonal anti-phospho-p38 (Cell Signaling, Cat#4511S; RRID: AB_2139682), Mouse polyclonal antibody anti-p38 (Cell Signaling, Cat#9212; RRID: AB_330713) Mouse monoclonal anti-phospho-SAPK/JNK (Cell Signaling, Cat#9255; RRID: AB_2307321), Rabbit monoclonal anti-phospho-SAPK/JNK (Cell Signaling, Cat#4668, RRID:AB_823588), Rabbit monoclonal anti-SAPK/JNK (Cell Signaling, Cat#9258; RRID: AB_2141027), Rabbit polyclonal anti-ZAK (Proteintech, Cat#14945-1-AP; RRID: AB_1064269), Mouse monoclonal anti-p150 (BD biosciences, Cat#610473, RRID: AB_397845), Mouse monoclonal anti-α-Tubulin (Merck, Cat#T9026, RRID: AB_477593), Mouse monoclonal anti-HA-tag (Santa Cruz Biotechnology, Cat#sc-7392 HRP, RRID: AB_2894930), Rabbit monoclonal anti-phospho-GCN2 (Abcam, Cat#ab75837, RRID: AB_1310587), Rabbit monoclonal anti-phospho-eIF2alpha (Cell Signaling, Cat#3398, RRID: AB_2096481), Rabbit polyclonal anti-cleaved Caspase 3 (Cell Signaling, Cat#9661, RRID:AB_2341188), Rabbit polyclonal anti-Caspase 3 (Santa Cruz, Cat#sc-7148, RRID:AB_637828), Rabbit monoclonal anti-phospho-Chk2 (Cell Signaling, Cat#2197, RRID:AB_2080501), Rabbit monoclonal anti-phospho-Chk1 (Cell Signaling, Cat#2348, RRID:AB_331212), Mouse monoclonal anti-γ-tubulin (Merck, Cat#T5326, RRID:AB_532292), Rabbit monoclonal anti-MK2 (Cell Signaling, Cat#3042, RRID: AB_2141314), Rabbit polyclonal anti-GSDMDC1 (Novus Bio, Cat#NBP2-33422, RRID:AB_2687913), Rabbit polyclonal anti-ERCC2 (Proteintech, Cat#10818-1-AP, RRID:AB_2231330), Rabbit monoclonal anti -IL1β p17 specific (Cell Signaling, Cat#83186, RRID:AB_2800010), Rabbit polyclonal anti-DDB2, Abcam, #ab181136, RRID:AB_2889873, Rabbit polyclonal anti-Cleaved PARP1 (Asp 214), Cell Signaling, #9541, RRID:AB_331426, Mouse monoclonal anti-GAPDH, Proteintech, 60004-1-Ig, RRID:AB_2107436.

RT-qPCR

Total RNA was purified using TRIzol reagent (Thermo Fisher Scientific, 15596026) according to the manufacturer's instructions. For reverse transcription 1000 ng of purified RNA were used with random hexamer primers and RevertAid RT Transcription Kit (Thermo Fisher Scientific Cat # K1691) according to manufacturer's protocol. For qPCR reactions 5 μl of 10-fold diluted cDNA were used together with 5x HOT FIREPol EvaGreen qPCR Mix Plus (NO ROX) (Bioline) according to the manufacturer's protocol. RNA abundances were deduced from ΔCt values, normalized to actin mRNA abundance, and compared to the corresponding control sample replicate.

See Table S2 for a list of oligonucleotides.

Live cell imaging and cell death quantification

2×10^5 N/TERT-1 cells were seeded in 12-well black plates (Cellvis, P12-1.5P) 24 hours prior to irradiation. Cell culture medium was replaced to contain ZAK, p38, JNK or ATR inhibitors for 1 hour, then the medium was removed before being exposed to 100 mJ/cm^2 of UVB irradiation using a BIO-SUN microprocessor-controlled, cooled UV irradiation system (BIO-SUN, Vilber). After exposure, 1 ml of keratinocyte medium with 0.3 μM of DRAQ7 stain (Abcam, ab109202) or 0.5 μg/ml of propidium iodide (PI, Abcam ab14083) or 10μl/ml pSIVA (Abcam, ab129817), as well as the indicated concentrations of inhibitors were added and cells were incubated in a high content screening microscope (Perkin Elmer Operetta CLS imaging system, NTU Optical Bio-Imaging Centre in Nanyang Technological University, Singapore). Brightfield, green and far-red images of the UV-irradiated cells were captured every 15 minutes for 14 hours. Images were then stored and analyzed using the Harmony software (Version 6). For at least 20 fields of view per well, the ratio of DRAQ7 positive cells over live cells was calculated. The number of live cells per field was counted using digital phase contrast images, which can identify cell borders, whereas the number of DRAQ7 stained nuclei was identified through the DRAQ5 channel (536 nm / 617 nm) and pSIVA+ cells were identified via the green channel. To calculate pyroptotic and apoptotic cells, 4 images captured at the indicated time points and treatments were selected, and cells were manually identified and calculated. Cells that were stained by DRAQ7, with the cell body largely intact but showing membrane swelling were considered as pyroptotic, while cells showing membrane blebbing and shriveling were considered apoptotic.

Different strains of HAP1 cells were seeded at approximately 25% confluence in a 48-well plate 24 hours prior to irradiation. On the day of the experiment, media was aspirated just before irradiation with 500 J/m2 UVB, after which new media heated to 37° C and supplemented with 0.3 μM DRAQ7 (Invitrogen, # D15105) dye was immediately added. Cells were subsequently placed in an Incucyte S3 Live-Cell Analysis System (Sartorius) with the phase contrast and red channels enabled for image acquisition at 10X magnification every two hours. Phase contrast images were subjected to cell segmentation using Cellpose3 [refsCellpose] after parameter refinement, and red images were used for detection of DRAQ7 signal over the resulting cell masks [refSkimage] after applying Gaussian blur and Top-Hat transforming to remove background signal. Cells with a maximum red intensity above threshold were classified as DRAQ7 positive.

RNAseq sample preparation and analysis

N/TERT-1 cells were grown to 80% confluence in a 6-well plate before performing treatments. Cells were irradiated or not with UVB and harvested after 5 hours. Total RNA was isolated from each treatment using RNAeasy Mini Kit (Qiagen, cat. #74004). The quantity and quality of each RNA sample was then assayed using the NanoDrop (Thermo Fisher Scientific) but also by running the RNA on an agarose gel to check for RNA degradation. RNA sequencing was performed at Macrogen Asia using the Novaseq 6000 platform. Library construction and sequencing followed the standard sequencing protocols and were performed by Macrogen Asia. Preprocessing and analysis were also performed by Macrogen Asia. In brief, reads were mapped to a reference genome (homo sapiens,

GRCh38) with HISAT2 and transcript assembled by StringTie with aligned reads. Expression profiles were represented as read count and normalization values which were calculated as TPM (Transcripts Per Kilobase Million) for each sample, based on transcript length and depth of coverage. Using pairwise comparisons, differentially expressed transcripts were calculated using an R package (DEseq2). The DEseq2 analysis was performed on read counts of expressed genes and for each gene the P-value and fold change were calculated per comparison pair.

QUANTIFICATION AND STATISTICAL ANALYSIS

Data in bar and line graphs are presented as mean ± SEM for mice or cell death data and ± SD for gene expression data. Statistical analyses were performed in GraphPad Prism 9 applying one or two-way ANOVA corrected for multiple comparisons with the Sidak method, respectively. For live imaing death curves, two-tailed Kolmogorov–Smirnov test at 95% confidence interval was performed. To distinguish between the two types of cell death, pyroptosis statistics was calculated based on DRAQ7 or PI curves between timepoint 0-5 hrs, and for apoptosis pSIVA curves for timepoint 5-14 hrs was used. ns., non-significant; *, $p \leq 0.05$; **, $p \leq 0.01$; ***, $p<0.001$; ****, $p \leq 0.0001$.

Molecular Cell, Volume *84*

Supplemental information

The ribotoxic stress response drives acute inflammation, cell death, and epidermal thickening in UV-irradiated skin *in vivo*

Anna Constance Vind, Zhenzhen Wu, Muhammad Jasrie Firdaus, Goda Snieckute, Gee Ann Toh, Malin Jessen, José Francisco Martínez, Peter Haahr, Thomas Levin Andersen, Melanie Blasius, Li Fang Koh, Nina Loeth Maartensson, John E.A. Common, Mads Gyrd-Hansen, Franklin L. Zhong, and Simon Bekker-Jensen

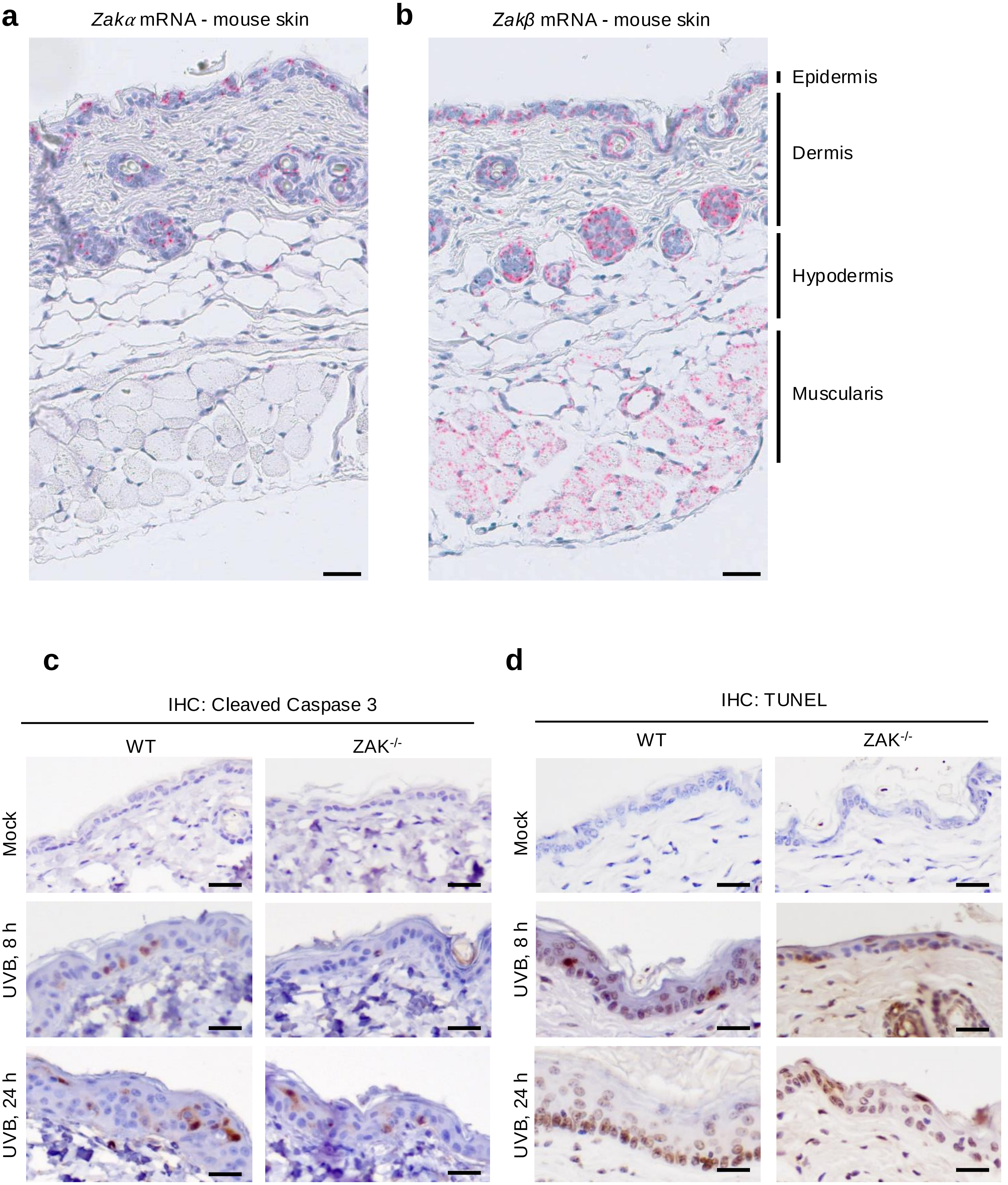
a
Zakα mRNA - mouse skin
b
Zakβ mRNA - mouse skin
Epidermis
Dermis
Hypodermis
Muscularis
c
IHC: Cleaved Caspase 3
WT
ZAK-/-
Mock
UVB, 8 h
UVB, 24 h
d
IHC: TUNEL
WT
ZAK-/-
Mock
UVB, 8 h
UVB, 24 h

Figure S1.

Zakα **and** ***Zakβ*** **expression in mouse skin (related to Fig. 1).**

a) Paraffin-embedded mouse skin was stained for *Zakα* mRNA (pink dots) by *in situ* hybridization with a modified RNAScope protocol. **b)** As in (a), except that samples were stained for *Zakβ* mRNA. Notice the specific expression of the β isoform in muscle fibers (muscularis layer). **c)** Skins of mice from Fig. 1c were harvested 8 and 24 hours after irradiation and immunostained with an antibody recognizing cleaved caspase-3. **d)** As in (c), except that skin sections were stained with TUNEL kit for detection of dead and dying cells. All scale bars = 50 µm.

Vind et al, Figure S2

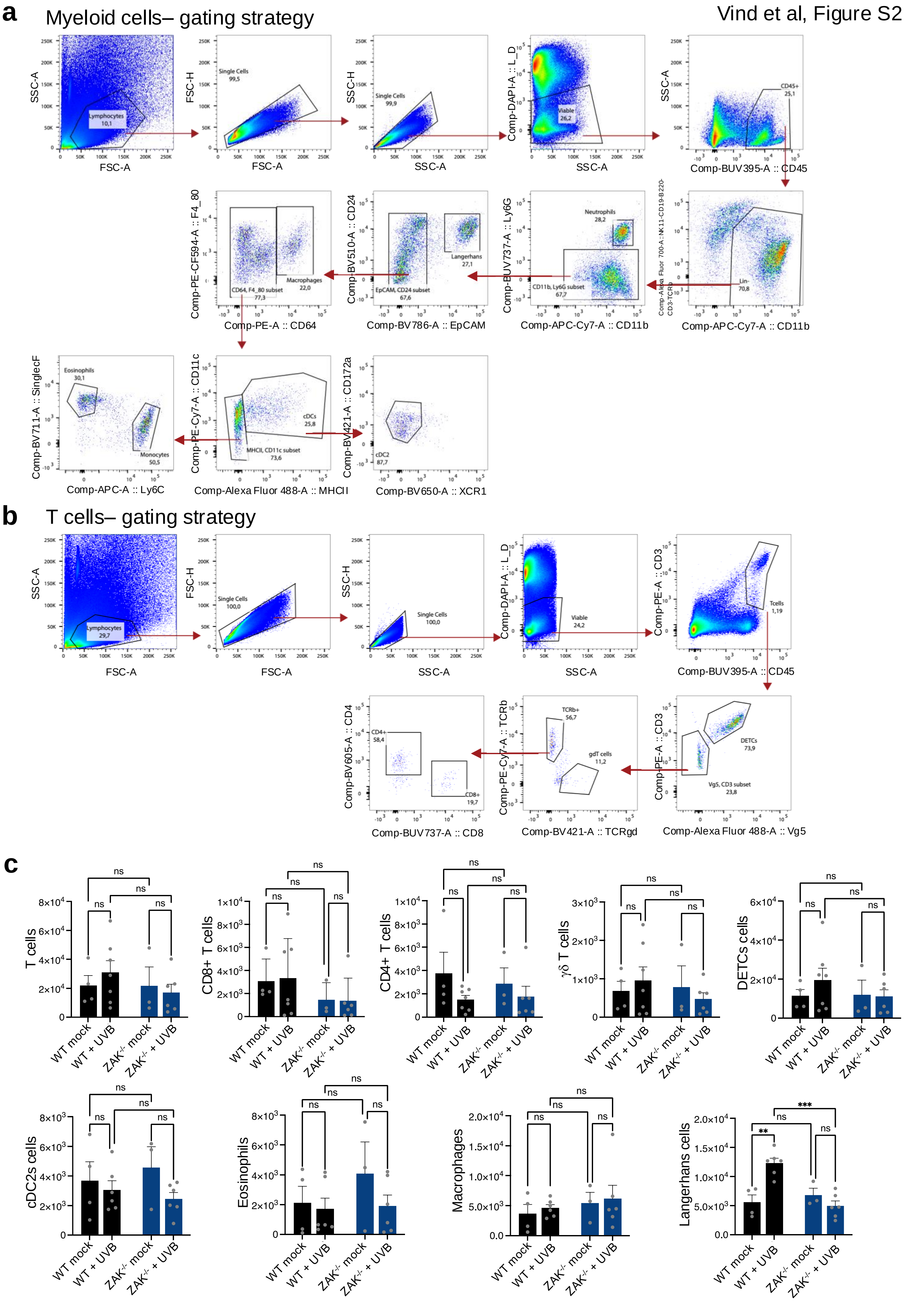

Figure S2.

Gating strategy for FACS-based analysis of dermal immune cell content (related to Fig. 2).

a-b) Schematic presentation of the gating strategy for flow cytometry analysis of **a**) myeloid and **b**) T cells in skin samples (Lineage: NK1.1, CD19, B220, CD3, TCRβ). **c**) Mouse skin samples were collected from control or UVB-exposed mice and immune infiltrates were analyzed by flow cytometry. Graphs represent the mean number of total T cells ($CD45^+$ $CD3^+$), $CD4^+$ T cells ($TCR\beta^+$ $CD4^+$), $CD8^+$ T cells ($TCR\beta^+$ $CD8^+$), γδ T cells ($CD3^+$, $TCR\gamma\delta^+$), dendritic epidermal T cells (DETCs; $CD3^+$, $V\gamma5^+$), cDC2s ($CD11c^+$ $MHCII^+$, $CD172\alpha^+$), eosinophils ($CD11b^+$ $SiglecF^+$), macrophages ($CD11b^+$, $F4/80^+$, $CD64^+$), Langerhans cells ($CD24^+$, $EpCAM^+$,). The data were obtained from two independent experiments with 3-7 mice per group. Data are presented as the mean, with error bars denoting the standard error of the mean (SEM). ns., non-significant; *, $p \leq 0.05$; **, $p \leq 0.01$; ***, $p < 0.001$; ****, $p \leq 0.0001$ in two-way ANOVA with the Sidak method.

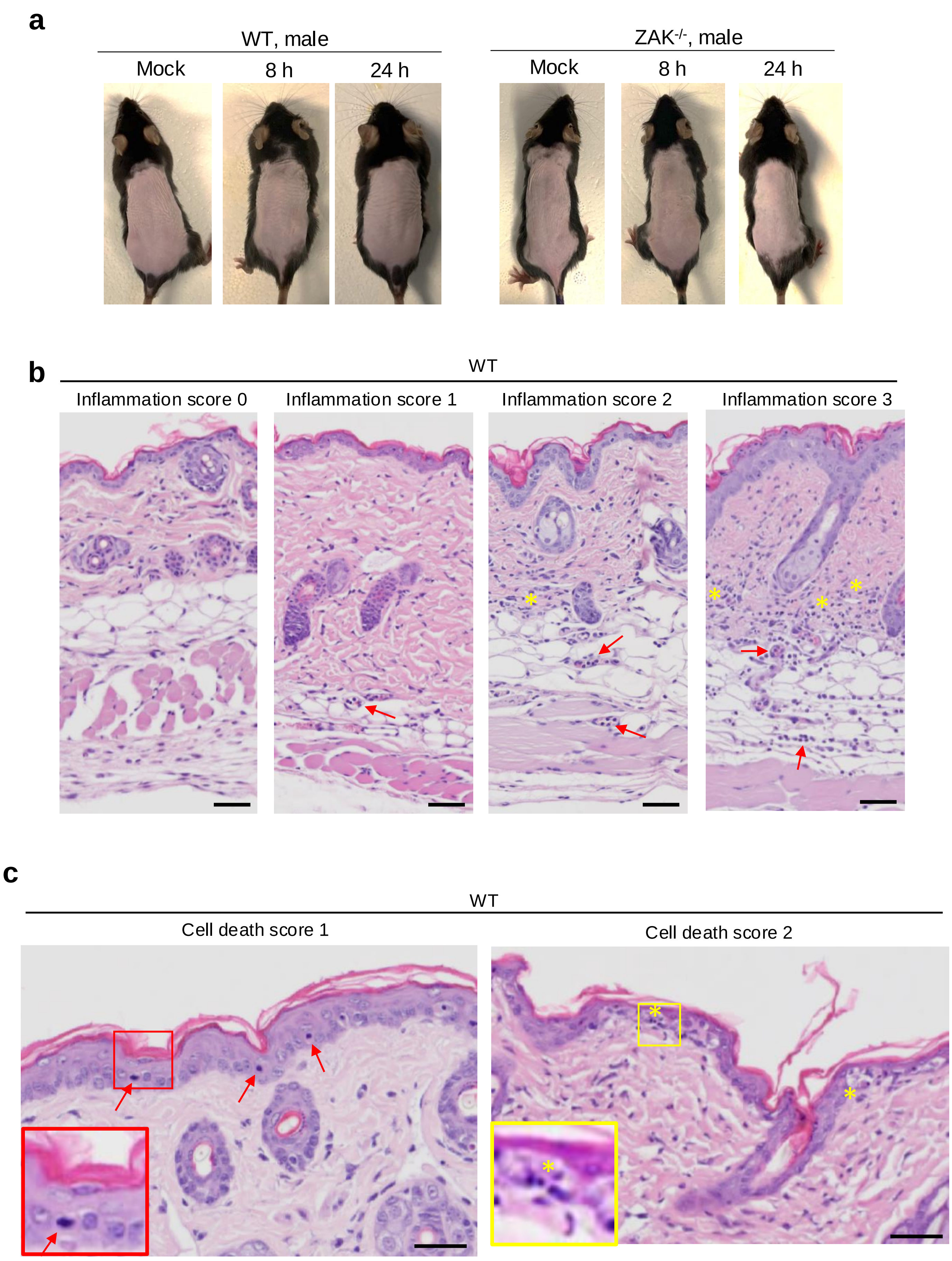
a
WT, male
Mock
8 h
24 h
ZAK-/-, male
Mock
8 h
24 h
b
WT
Inflammation score 0
Inflammation score 1
Inflammation score 2
Inflammation score 3
c
WT
Cell death score 1
Cell death score 2

Figure S3.

Scoring system for histological hallmarks of skin inflammation and cell death (related to Fig. 2).

a) Representative images of male mice 24 h and 48 h after UVB-irradiation (500 J/m^2). **b)** Hematoxylin and eosin (H/E)-stained mouse skin sections representing inflammatory scores 0-3. Red arrows indicate intraluminal infiltration and yellow asterisks indicate dermal infiltration of immune cells. **c)** Examples of discrete (upper panel, yellow arrows) and confluent (lower panel, yellow asterisks) cell death in UVB-irradiated and hematoxylin and eosin (H/E)-stained mouse skin. All scale bars = 50 µm.

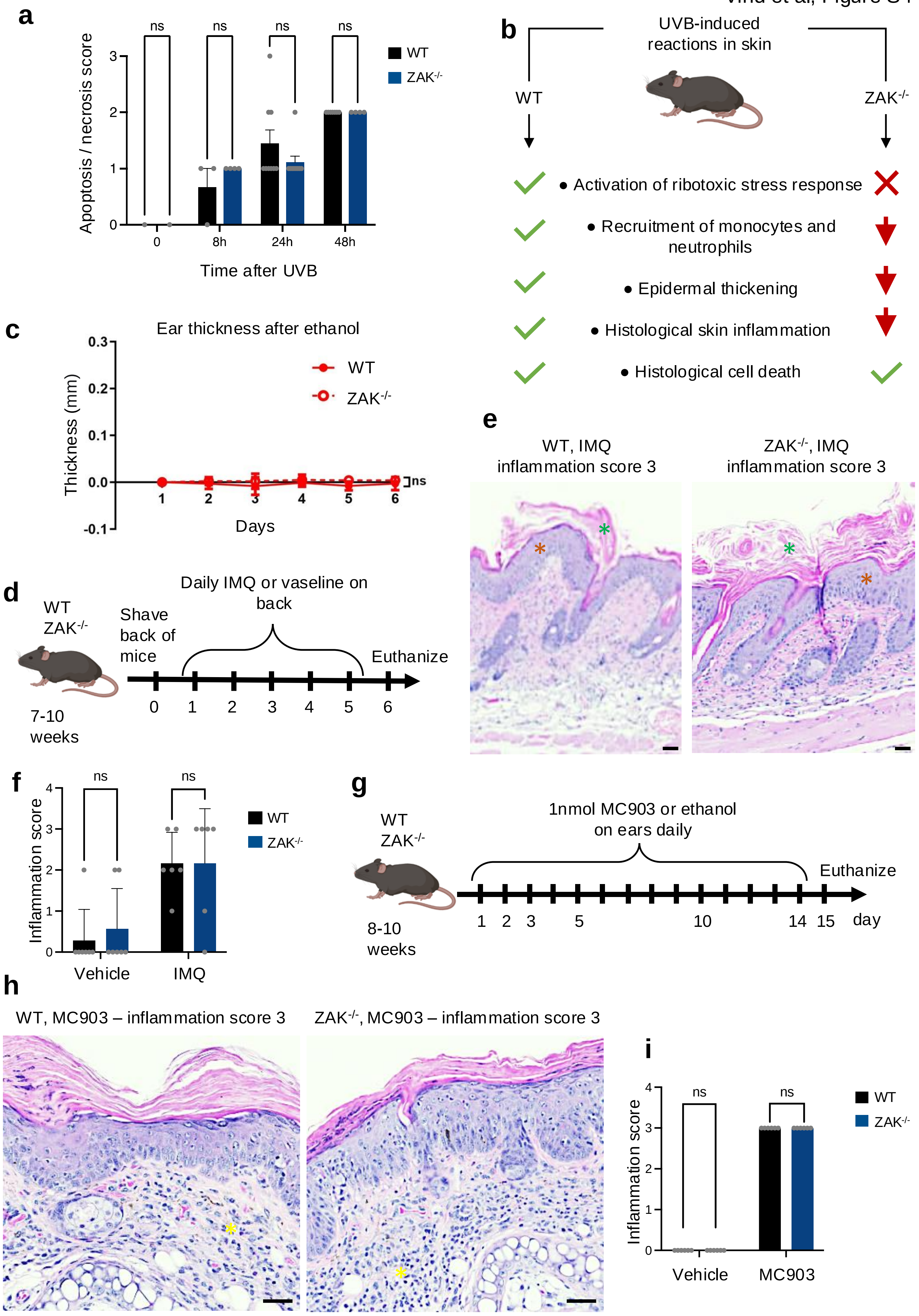
a
ns
Apoptosis / necrosis score
WT
ZAK-/-
0
8h
24h
48h
Time after UVB
b
UVB-induced reactions in skin
WT
ZAK-/-
• Activation of ribotoxic stress response
• Recruitment of monocytes and neutrophils
• Epidermal thickening
• Histological skin inflammation
• Histological cell death
c
Ear thickness after ethanol
Thickness (mm)
Days
ns
d
WT
ZAK-/-
7-10 weeks
Shave back of mice
Daily IMQ or vaseline on back
Euthanize
e
WT, IMQ
inflammation score 3
ZAK-/-, IMQ
inflammation score 3
f
Inflammation score
Vehicle
IMQ
g
WT
ZAK-/-
8-10 weeks
1nmol MC903 or ethanol on ears daily
Euthanize
day
h
WT, MC903 – inflammation score 3
ZAK-/-, MC903 – inflammation score 3
i
Inflammation score
Vehicle
MC903

Figure S4.

ZAK$^{-/-}$ mice are not protected against inflammation and epidermal thickening in standard models of inflammatory skin disease (related to Fig. 2).

a) Extent of cell death in 9-week-old female WT and ZAK$^{-/-}$ mice at 8, 24 and 48 hours after UVB-irradiation (500 J/m^2) was assessed histologically based on the following scoring system. Normal: 0. Discrete apoptotic and necrotic cells: 1. Presence of areas of confluent apoptotic and necrotic cells: 2. Representative images showed in Fig. S3c. **b)** The ribotoxic stress response is activated in mouse skin upon UVB-irradiation and mediates several of the well-known adaptive responses. UVB-irradiated ZAK$^{-/-}$ mouse skin is refractory to early inflammation and displays attenuated and delayed epidermal thickening. Keratinocyte cell death is equally pronounced in UVB-irradiated WT and ZAK$^{-/-}$ mouse skin. **c)** Growth in ear thickness of mice from Fig. 3c were measured with a caliper. Only data from vehicle-treated ears are shown. **d)** Schematic of *in vivo* mouse imiquimod (IMQ) exposure experiment. IMQ was dissolved in Vaseline (vehicle) and applied to the shaved backs of 7-10-week-old female WT and ZAK$^{-/-}$ mice for 5 consecutive days. Mice were euthanized on day 6. **e)** Representative H/E-stained images of back skin from (d). Green asterisks indicate hyperparakeratosis. Brown asterisks indicate epidermal hyperplasia. **f)** Severity of inflammation in back skins from (d) was assessed histologically based on infiltrating immune cells according to the scoring system described in the legend of Fig. 2g. **g)** Schematic of *in vivo* mouse MC903 exposure experiment. MC903 was dissolved in ethanol (vehicle) and applied to one ear of 8-10-week-old female WT and ZAK$^{-/-}$ mice for 14 consecutive days. Pure ethanol was similarly applied to the other ear and mice were euthanized on day 15. **h)** Representative H/E-stained images of ears from (c). Yellow asterisks indicate dermal infiltration of immune cells. **i)**. Ear skin inflammation of mice from (g) was scored as in (f). Data are plotted as mean and all error bars represent the standard error

of the mean (SEM) (n=3-9 biological replicates). ns., non-significant in two-way ANOVA with the Sidak method. All scale bars = 50 μm.

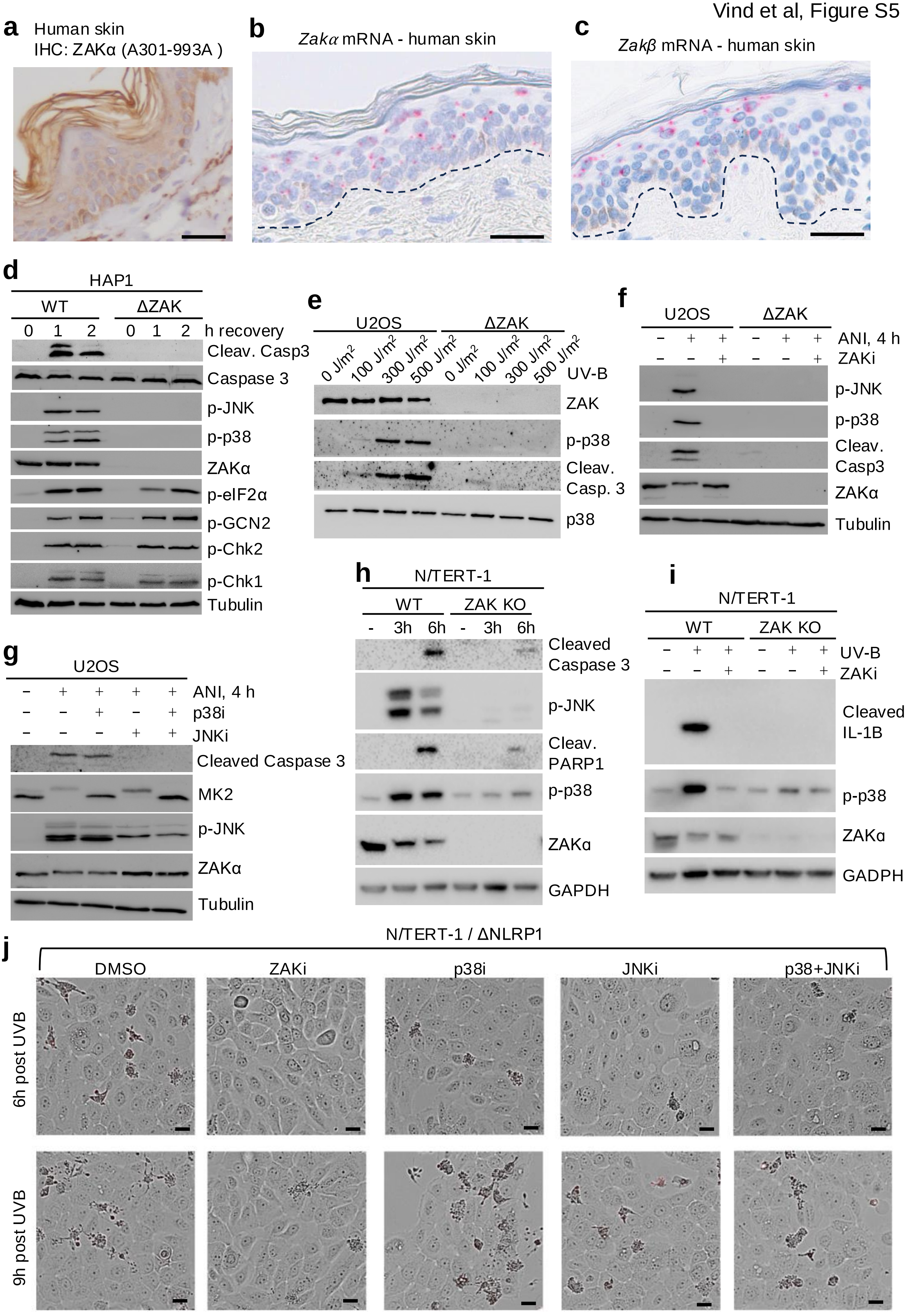
a
Human skin
IHC: ZAKα (A301-993A)
b
Zakα mRNA - human skin
c
Zakβ mRNA - human skin
d
HAP1
WT
ΔZAK
0 1 2 0 1 2 h recovery
Cleav. Casp3
Caspase 3
p-JNK
p-p38
ZAKα
p-eIF2α
p-GCN2
p-Chk2
p-Chk1
Tubulin
e
U2OS
ΔZAK
0 J/m2 100 J/m2 300 J/m2 500 J/m2 0 J/m2 100 J/m2 300 J/m2 500 J/m2
UV-B
ZAK
p-p38
Cleav. Casp. 3
p38
f
U2OS
ΔZAK
ANI, 4 h
ZAKi
p-JNK
p-p38
Cleav. Casp3
ZAKα
Tubulin
g
U2OS
ANI, 4 h
p38i
JNKi
Cleaved Caspase 3
MK2
p-JNK
ZAKα
Tubulin
h
N/TERT-1
WT
ZAK KO
- 3h 6h - 3h 6h
Cleaved Caspase 3
p-JNK
Cleav. PARP1
p-p38
ZAKα
GAPDH
i
N/TERT-1
WT
ZAK KO
UV-B
ZAKi
Cleaved IL-1B
p-p38
ZAKα
GADPH
j
N/TERT-1 / ΔNLRP1
DMSO
ZAKi
p38i
JNKi
p38+JNKi
6h post UVB
9h post UVB

Figure S5.

The ribotoxic stress response mediates UVB-induced and JNK-dependent apoptotic cell death (related to Fig. 4).

a) Paraffin-embedded human skin was analyzed for ZAKα expression (#A301-993A) by immunohistochemistry. Scale bars = 50 μm. **b)** Paraffin-embedded human skin was stained for *Zakα* mRNA (pink dots) by *in situ* hybridization with a modified RNAScope protocol. Dashed line marks the separation between epidermis and dermis. Scale bar = 10 μm. **c)** As in (b), except that *Zakβ* mRNA was detected. **d)** HAP1 WT and ΔZAK cells were UVB irradiated (500 J/m^2) and allowed to recover for the indicated times. Lysates were analyzed by immunoblotting with the indicated antibodies. **e)** U2OS WT and ΔZAK cells were irradiated with the indicated doses of UVB (8 hours recovery). Lysates were analyzed as in (d). **f)** Cells from (e) were treated with anisomycin (ani, 1 μg/ml - 4 hours) in the presence of ZAK inhibitor (i, 1 μM) as indicated. Lysates were analyzed as in (d). **g)** U2OS cells were treated with ani (1 μg/ml - 4 hours) in the presence of inhibitors (i) of p38 and JNK (2 μM). Lysates were analyzed as in (d). **h)** WT and ΔZAK N/TERT-1 cells were irradiated with UVB (100 J/m^2) and lysed at the indicated timepoints. Lysates were analyzed as in (d). **i)** As in (h), except that cells were pre-treated with ZAK inhibitor (i, 1 μM) and lysed 8 h after UVB irradiation. **j)** Representative bright-field microscopy images of NLRP1 KO N/TERT-1 cells in combination with the indicated inhibitor 6 hours and 9 hours post UVB to show apoptotic/necrotic cell death. Scale bars = 10 μm.

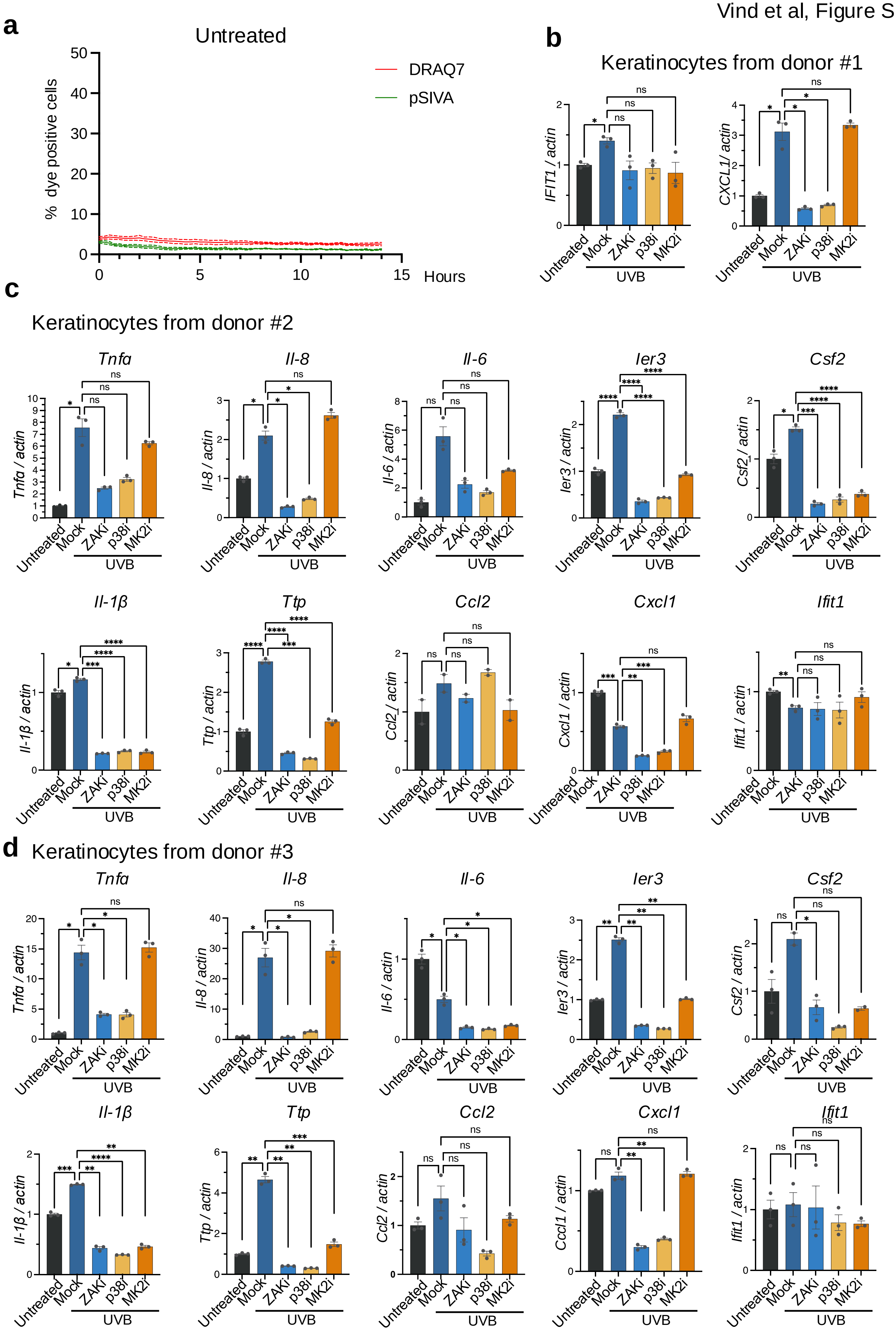
a
Untreated
% dye positive cells
DRAQ7
pSIVA
Hours
b
Keratinocytes from donor #1
IFIT1 / actin
CXCL1/ actin
Untreated
Mock
ZAKi
p38i
MK2i
UVB
c
Keratinocytes from donor #2
Tnfα
Il-8
Il-6
Ier3
Csf2
Il-1β
Ttp
Ccl2
Cxcl1
Ifit1
d
Keratinocytes from donor #3
Tnfα
Il-8
Il-6
Ier3
Csf2
Il-1β
Ttp
Ccl2
Cxcl1
Ifit1
Ccl1 / actin

Figure S6.

Upregulation of inflammatory transcripts in UVB-irradiated primary human keratinocytes (related to Fig. 5).

a) N/TERT-1 cells were grown in the presence of the live cell staining dyes DRAQ7 (permeabilized cells) and pSIVA (apoptosis marker) and subjected to fluorescence time-lapse microscopy. Percentage of dye-including cells are plotted as mean and error bars represent the standard error of the mean (SEM). Error bars are from 3 technical replicates that are representative of 2 independent repeats. **b)** Primary human keratinocytes from donor in Fig. 5f were UVB irradiated (500 J/m^2 – 6 hours) in the presence of inhibitors (i, 2 μM) against the kinases ZAK, p38 and JNK as indicated. qPCR analysis of isolated RNA was performed using primers against the indicated transcripts. Values were normalized to actin expression and transformed into fold changes over the values from untreated cells. **c)** and **d)** as in (b), but with primary keratinocytes from two independent human donors. Fold-change data are plotted as mean and all error bars represent the standard deviation (SD) (n=3 technical replicates). ns., non-significant; *, $p \leq 0.05$; **, $p \leq 0.01$; ***, $p < 0.001$; ****, $p \leq 0.0001$ in two-way ANOVA with the Sidak method.

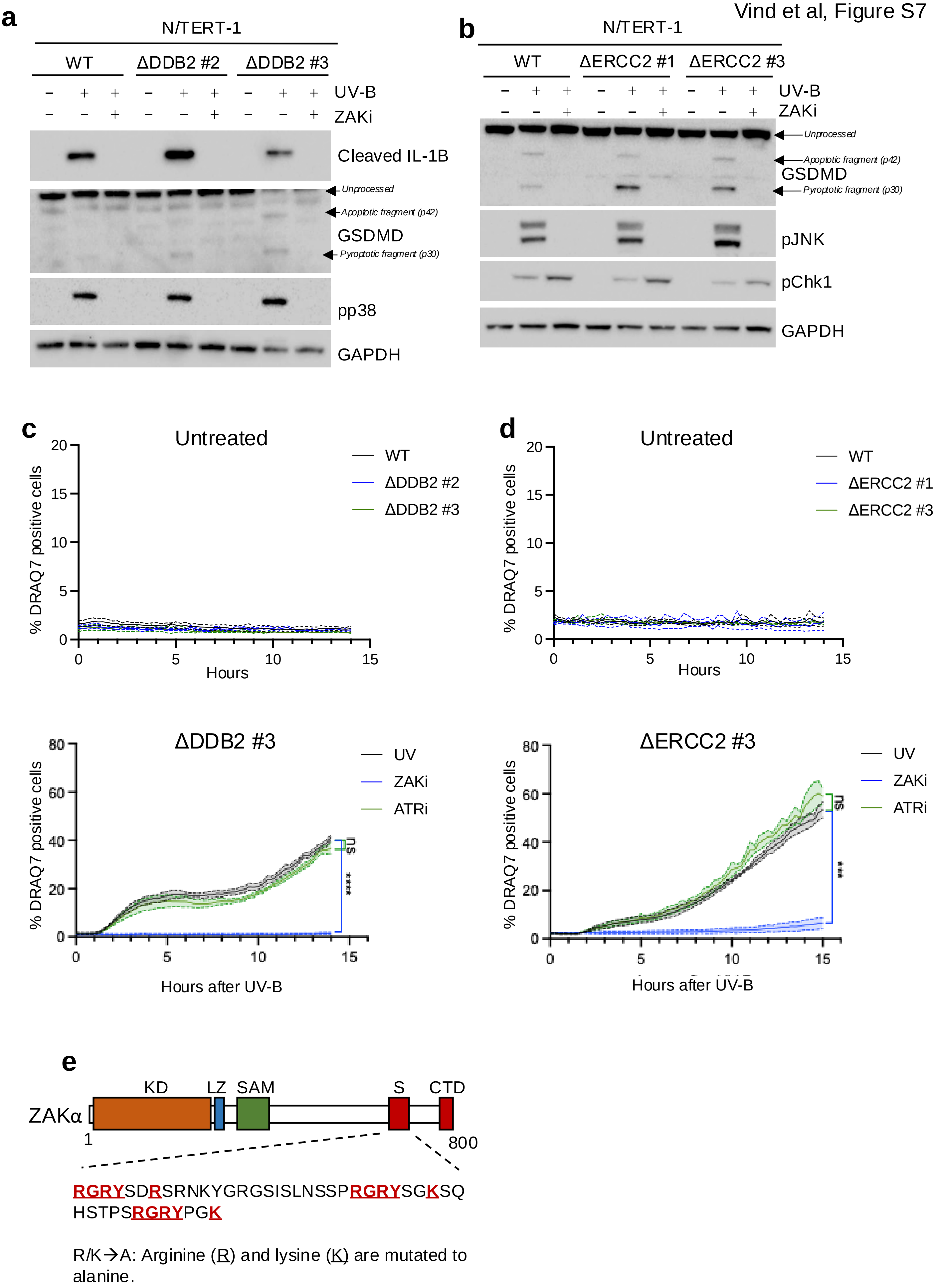
a
N/TERT-1
WT
ΔDDB2 #2
ΔDDB2 #3
UV-B
ZAKi
Cleaved IL-1B
Unprocessed
Apoptotic fragment (p42)
GSDMD
Pyroptotic fragment (p30)
pp38
GAPDH
b
N/TERT-1
WT
ΔERCC2 #1
ΔERCC2 #3
UV-B
ZAKi
Unprocessed
Apoptotic fragment (p42)
GSDMD
Pyroptotic fragment (p30)
pJNK
pChk1
GAPDH
c
Untreated
% DRAQ7 positive cells
Hours
WT
ΔDDB2 #2
ΔDDB2 #3
d
Untreated
% DRAQ7 positive cells
Hours
WT
ΔERCC2 #1
ΔERCC2 #3
ΔDDB2 #3
% DRAQ7 positive cells
Hours after UV-B
UV
ZAKi
ATRi
ns
****
ΔERCC2 #3
% DRAQ7 positive cells
Hours after UV-B
UV
ZAKi
ATRi
ns
***
e
KD
LZ
SAM
S
CTD
ZAKα
1
800
RGRYSDRSRNKYGRGSISLNSSPRGRYSGKSQ
HSTPSRGRYPGK
R/K→A: Arginine (R) and lysine (K) are mutated to alanine.

Figure S7.

The DNA damage response is dispensable for induction of early markers of UVB-induced pyroptosis and apoptosis (related to Fig. 6).

a) N/TERT-1 cells deleted for DDB2 (ΔDDB2) were treated with ZAK inhibitor (1 μM) and UVB-irradiated (100 J/m^2 – 8 h recovery). Lysates were analyzed by immunoblotting with the indicated antibodies. **b)** As in (a), except that cells were deleted for ERCC2 (ΔERCC2). **c)** WT and ΔDDB2 cells from (a) were grown in the presence of the live cell staining dye DRAQ7 and subjected to fluorescence time-lapse microscopy (top) or treated with indicated inhibitors (i, 1 μM), irradiated with UVB (100 J/m^2) and subjected to fluorescence time-lapse microscopy (bottom). **d)** As in (c), except that WT cells were compared to ΔERCC2 cells from (b)**.** Statistical significance for pyroptosis was calculated by two-tailed Kolmogorov–Smirnov test at 95% confidence interval between 0-5 hrs. ns., non-significant; ***, $p<0.001$; ****, $p\leq0.0001$. **e)** Schematic of the ZAKα protein highlighting the three RGRYXXR/K motifs in the sensor (S) domain. These motifs are mutated to AGAYXXA in the R/K->A ZAKα mutants used in Fig. 6j. KD, Kinase Domain; LZ, Leucine Zipper; SAM, Sterile Alpha Motif; CTD, C-Terminal Domain. Percentage of dye-including cells are plotted as mean and error bars represent the standard error of the mean (SEM) for three technical replicates.
